# Isolation and characterization of antiplasmodial and antimicrobial compounds from *Tetracera alnifolia* using a bioassay-guided approach

**DOI:** 10.1039/d5ra09498d

**Published:** 2026-02-23

**Authors:** Mamadou Aliou Baldé, Mohamed Sahar Traoré, Mamadou Saliou Telly Diallo, An Matheeussen, Camara Aïssata, Paul Cos, Louis Maes, Alpha Oumar Baldé, Mohamed Kerfala Camara, Aliou Mamadou Balde, Emmy Tuenter, Kenn Foubert

**Affiliations:** a Department of Pharmaceutical and Biological Sciences, University Gamal Abdel Nasser of Conakry Guinea baldem@ufl.edu; b Natural Products & Food Research and Analysis – Pharmaceutical Technology (NatuRAPT), Department of Pharmaceutical Sciences, University of Antwerp Universiteitsplein 1 B-2610 Antwerp Belgium; c Laboratory for Microbiology, Parasitology and Hygiene (LMPH), Faculty of Pharmaceutical, Biomedical and Veterinary Sciences, University of Antwerp Universiteitsplein 1 B-2610 Antwerp Belgium; d Research Institute of Applied Biology of Guinea (IRBAG) Kindia Guinea

## Abstract

A preliminary biological screening demonstrated that leaf extracts from *Tetracera alnifolia* exhibit promising activity against *Plasmodium falciparum* and *Candida albicans*. To identify the constituents responsible for these effects, a bioassay-guided fractionation strategy was subsequently employed. Fractions were purified using flash chromatography, followed by semi-preparative HPLC-DAD-MS and LC-SPE-NMR. Structural elucidation of the isolated compounds was accomplished through comprehensive 1D and 2D NMR analyses in combination with HR-ESI-MS. The purified metabolites were subsequently evaluated for their bioactivity against *Plasmodium falciparum* and *Candida albicans*, and their cytotoxicity was assessed in MRC-5_SV2_ human foetal lung fibroblast cells. In total, nineteen compounds (1–19) were isolated from *T. alnifolia* leaves. Among these, the strongest antiplasmodial activities were observed for pheophorbide-*b* methyl ester (1.0 ± 0.7 µM), (1,2)-bis-nor-phytone (2.0 µM), isophytol (4.0 µM), pheophorbide-*a* methyl ester (2.8 ± 1.2 µM), epicatechin-3-galloyl ester (5.5 ± 2.1 µM), and phytol (6.9 ± 2.4 µM). Other compounds, including myricetin-3-*O*-rhamnopyranoside, α-tocopherol and cycloart-24-en-3β-yl α-linolenate, exhibited lower activity, with IC_50_ values ranging from 13.5 to 25 µM. None of the evaluated compounds showed antifungal activity against *C. albicans*. Notably, high cytotoxicity was observed for pheophorbide-*b* methyl ester, pheophorbide-*a* methyl ester, and phytol. These findings provide insight into the constituents that underlie the antiplasmodial activity of *T. alnifolia* leaf extracts.

## Abstract 2

Bioassay-guided fractionation workflow of *T. alnifolia* leaf extracts leading to the isolation of active compounds against *Plasmodium falciparum* and *Candida albicans*.

## Graphical abstract

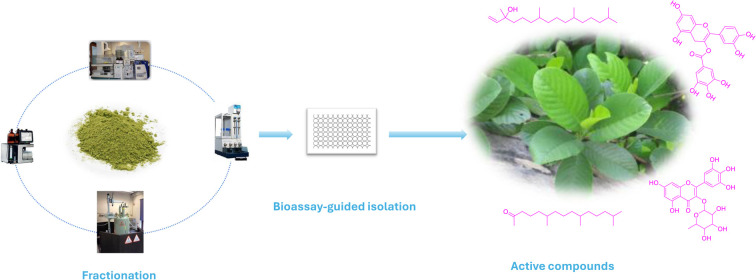

## 1 Introduction

The discovery of new bioactive natural products as leads for therapeutic development can be inspired by ethnopharmacological knowledge or achieved by screening a collection of extracts for bioactivity, using several bioassays.^1,2^ The traditional workflow for studying complex plant preparations is bioactivity-guided fractionation in which bioactive extracts and subsequent fractions are chromatographically separated and retested for bioactivity until active compounds have been isolated.^3,4^ In the last decade, substantial developments have been made in improving extraction and separation efficiency and facilitating the isolation of minor constituents that may contribute to activity.^5^ Also, nowadays, the bioassay-guided fractionation workflow is still used by scientists for the discovery of new therapeutic leads from natural sources, including plant species.^1^

The Dilleniaceae family comprises 10–14 genera and *ca.* 500 species, with a pantropical and subtropical distribution. Around 102 species distributed across six genera *viz. Curatella*, *Davilla*, *Doliocarpus*, *Neodillenia*, *Pinzona*, and *Tetracera* are mainly found in the Neotropical region. The Dilleniaceae family is divided into four subfamilies and the genus *Tetracera* belongs to the Delimoideae subfamily.^6,7^ The genus *Tetracera* contains about 50 species with a pantropical distribution, of which 20 occur in the Neotropical area. In traditional folk medicine, some species have been used for the management of various diseases and infections.^8,9^ Species of this genus have been reported to exhibit a broad range of pharmacological activities, including antifungal, antiplasmodial, analgesic, anti-inflammatory, antioxidant, antipyrexic, antimycobacterial, anti-HIV, anti-ulcerogenic and hepatoprotective.^8,10^ Several *Tetracera* species have previously been investigated for their phytochemical constituents, which resulted in the identification of a broad range of compounds, including flavonoids and terpenoids.^10,11^ Within the Guinean flora, approximately four species of the genus *Tetracera* have been identified, among which *T. alnifolia* is distributed across all regions of Guinea. *T. alnifolia*, a forest liana up to 15 m high, is widely used in Guinean traditional medicine for the management of several diseases including skin diseases, oral diseases, infectious diseases and malaria.^12–15^ This plant species is also well known in other African countries for its traditional uses in the management of inflammation, pain, cough, sexually transmitted infections and leprosy.^9,16,17^ Promising *in vitro* activities against *P. falciparum* and *C. albicans* were previously obtained in our laboratory for both methanolic and dichloromethane extracts of the leaves of *T. alnifolia*.^18^ This study aimed to isolate metabolites potentially responsible for the observed biological activities, which may serve as promising precursors for the development of novel antimicrobial and/or antiplasmodial agents. In this context, a bioguided fractionation of leaf extracts from *T. alnifolia* was conducted.

## 2 Materials and methods

### 2.1. Solvent and reagents

HPLC-grade acetone, acetonitrile (including far-UV grade), dichloromethane, ethanol, ethyl acetate, methanol, *n*-butanol, and *n*-hexane were sourced from Fisher Scientific (Leicestershire, UK). Formic acid, sulfuric acid and vanillin were supplied by Acros Organics (Geel, Belgium) or Sigma-Aldrich (St. Louis, MO, USA). For UPLC-MS analyses, acetonitrile and formic acid were purchased from Biosolve Chimie (Dieuze, France). Ultrapure water was produced using a Milli-Q purification system (Millipore, Bedford, MA, USA). Deuterated solvents for NMR spectroscopy (acetonitrile-*d*_3_, CDCl_3_, D_2_O, DMSO-*d*_6_, methanol-*d*_4_, and pyridine-*d*_5_) were purchased from Sigma-Aldrich.

### 2.2. General experimental methods

Extracts and derived fractions were separated using Diaion HP-20 open-column chromatography in combination with a Reveleris X2 flash chromatography system (BUCHI Labortechnik, Breda, Netherlands) equipped with ELSD, DAD, and automated fraction collection. Chromatographic profiles of fractions collected throughout the isolation workflow were analyzed using an Agilent HPLC-DAD system (1200 Series) and/or thin-layer chromatography (TLC) on NP-F254 plates (20 cm × 20 cm; Merck, Darmstadt, Germany). TLC plates were examined under UV light at 254 and 366 nm, as well as under visible light after derivatization with vanillin sulfuric acid reagent (prepared by dissolving 5 g vanillin in 475 mL ethanol and adding 25 mL sulfuric acid). Pure compounds were isolated using a semi-preparative HPLC platform equipped with DAD and ESI-MS detection. The system comprised a 2767 sample manager, injector, and collector, a 2545 quaternary gradient module, a System Fluidics Organizer, a 515 HPLC pump, a 2998 DAD, and a Micromass Quattro TQD mass spectrometer (Waters, Milford, MA, USA). Data were processed using MassLynx version 4.1.

Low yield subfractions (<100 mg) were purified using HPLC-SPE-NMR. Analyses were performed on an Agilent 1200 Series HPLC system (Agilent Technologies) equipped with a degasser, quaternary pump, autosampler, and photodiode-array (PDA) detector. Target analytes were cumulatively trapped on SPE cartridges by UV-threshold-triggered collection. Downstream of the detector, water was introduced at 3 mL min^−1^*via* a make-up pump (Knauer K-120, Berlin, Germany) to enhance retention on the SPE cartridges. Trapping was carried out on a Bruker/Spark SPE platform fitted with HySphere Resin General Phase (GP) cartridges (poly(divinylbenzene), 5–15 µm). After the final trapping, each cartridge was dried with pressurized nitrogen gas for 40 min and eluted with 60 µL of deuterated solvent (acetonitrile-*d*_3_ or methanol-*d*_4_) into 3 mm NMR tubes with a Gilson Liquid Handler 125 (The Hague, Netherlands). Chromatographic separation and analyte trapping on SPE cartridges were controlled using Hystar v. 3.2 software (Bruker Daltonik, Bremen, Germany), whilst the elution process was controlled by Prep Gilson ST ver. 1.2 software (Bruker Biospin, Karlsruhe, Germany). Nuclear magnetic resonance (NMR) experiments, including both one and two dimensional measurements, were carried out using a Bruker DRX-400 spectrometer (Bruker BioSpin, Rheinstetten, Germany). The instrument was fitted with either a 3 mm inverse broadband (BBI) probe or a 5 mm dual ^1^H/^13^C probe, and experiments were conducted using routine Bruker pulse sequences. Data were collected at operating frequencies of 400 MHz for proton (^1^H) and 100 MHz for carbon (^13^C). All spectra were processed and interpreted using TopSpin software (version 4.0.6, Bruker BioSpin).

High-resolution mass spectrometric measurements were obtained using a Xevo G2-XS QTof instrument (Waters, Milford, MA, USA) coupled to an ACQUITY UPLC chromatographic platform and operated *via* MassLynx software (v4.1). Chromatographic separation was performed on a Waters Acquity UHPLC BEH Shield RP18 column (2.1 × 100 mm, 1.7 µm particle size). The elution system consisted of water supplemented with 0.1% formic acid (solvent A) and acetonitrile supplemented with 0.1% formic acid (solvent B), with a constant flow rate of 0.40 mL min^−1^. The gradient profile was set as follows: initial 2% B (0–1 min), increased to 100% B over 1–5 min, maintained at 100% B for 5–7 min, returned to 2% B during 7–8 min, and equilibrated at 2% B from 8–10 min. Mass detection was carried out using electrospray ionization operated in both positive and negative modes, with data acquired over an *m*/*z* interval of 50–1500 under sensitivity mode conditions (≈22 000 FWHM). The capillary voltage was adjusted to +1.5 kV for positive ionization and −1.0 kV for negative ionization. Cone gas and desolvation gas flow rates were set to 50 and 1000 L h^−1^, respectively. The ion source temperature was maintained at 120 °C, while the desolvation temperature was set to 550 °C. Continuous mass accuracy was ensured through lock-mass correction using leucine enkephalin.

### 2.3. Plant material

*T. alnifolia* leaves were harvested in June 2017 in Telimélimé, Republic of Guinea. Species authentication was performed by botanists at the Research and Valorization Center on Medicinal Plants (Dubréka, Guinea), where a voucher specimen (D42HK2) has been deposited. The collected material was air-dried at room temperature and subsequently ground into a fine powder.

### 2.4. Extraction, bioguided fractionation and isolation

The air-dried and ground leaves of *T. alnifolia* (780 g) were initially defatted with *n*-hexane (5 × 2.5 L) and subsequently extracted with 80% methanol (5 × 2.5 L) at room temperature under constant magnetic stirring. For each solvent, the plant material was macerated for 24 h, with the solvent replaced daily over a total period of five days. Pooled filtrates were concentrated under reduced pressure and freeze-dried to obtain the hexane-soluble fraction (*T*_E_) (12 g) and the crude methanolic fraction (*T*_M_) (207 g) which was redissolved in water and partitioned with CH_2_Cl_2_ to give a dichloromethane soluble fraction (*T*_D_) (10 g). Based on the biological activity results, the active fractions (hexane, dichloromethane and methanol) were selected and further purified by open column chromatography, flash chromatography semi preparative HPLC-DAD-MS and LC-SPE-NMR.

#### 2.4.1. Purification of the hexane fraction

The hexane fraction (*T*_E_, 9.3 g) was purified by flash chromatography on a silica gel column (GraceResolv, 120 g) using *n*-hexane (A), dichloromethane (B), and ethyl acetate (C) as eluents at a flow rate of 40 mL min^−1^. The elution profile consisted of 100% A for 0–15 min, followed by a linear increase of B in A from 15–65 min to reach 50% B, which was held for 5 min (65–70 min). The proportion of B was then further increased from 70–90 min to reach 100% B. Subsequently, C was introduced and increased to 100% from 90–105 min, and this final composition was maintained for an additional 10 min (105–115 min). Collected eluates were combined into 6 fractions (*T*_E1_–*T*_E6_) based on their TLC profiles (mobile phase: *n*-hexane/dichloromethane, 1 : 9, v/v; detection at 254 nm). Fraction *T*_E2_ (2.3 g) was further purified by flash chromatography on a silica gel column (GraceResolv, 40 g) using *n*-hexane (A) and dichloromethane (B) as eluents at a flow rate of 40 mL min^−1^, affording compounds 1 (16.0 mg) and 2 (56.0 mg). The elution gradient consisted of 100% A from 0–5 min; a gradual increase of B from 5–55 min to reach 20% B; a further increase to 35% B from 55–65 min; and finally, a rise to 100% B from 65–70 min. The column was held at 100% B for an additional 5 min (70–75 min). Fractions *T*_E3_ and *T*_E4_ were purified by preparative TLC using dichloromethane/*n*-hexane (1 : 1, v/v) and toluene (100%) as mobile phases, respectively, with detection at 254 nm. *T*_E3_ afforded compound 3 (7.5 mg), whereas *T*_E4_ yielded compounds 4 (3.2 mg) and 5 (8.2 mg).

#### 2.4.2. Purification of the dichloromethane fraction

The dichloromethane fraction (*T*_D_, 5 g) was purified by flash chromatography on a silica gel column (GraceResolv, 80 g) using *n*-hexane (A), dichloromethane (B), ethyl acetate (C), and methanol (D) as eluents at a flaw rate of 60 mL min^−1^. The gradient elution was programmed as follows: 100% A (0–8 min), followed by an increasing concentration of B in A to 100% B (8–48 min); linear increase of C in B to 100% C (48–75 min); then increase of D in C to 50% D (75–100 min). The final composition was held for 10 min (total gradient run time: 110 min). Eluates were combined into eight fractions (*T*_D1_–*T*_D8_) based on TLC profiles (dichloromethane/ethyl acetate, 8 : 2, v/v). Preparative TLC of *T*_D1_ (0.3 g) with dichloromethane/*n*-hexane (9 : 1, v/v) afforded compound 6 (13.5 mg), and *T*_D2_ yielded compounds 7 (4.2 mg) and 8 (3.7 mg). Fraction *T*_D4_ (0.77 g) was further purified by flash chromatography over a silica gel column (GraceResolv, 40 g), employing dichloromethane (A), ethyl acetate (B), and methanol (C) as mobile phases at a flow rate of 40 mL min^−1^. This separation yielded compounds 9 (14.6 mg), 10 (15.3 mg), and 11 (9.4 mg). The gradient was programmed as follows: 100% A (0–10 min); a linear increase of B to 100% (10–48 min); followed by the increasing amount of C to reach 20% (48–78 min). The final composition was held for 10 min (78–88 min). Compound 12 (4.5 mg) was isolated from fraction *T*_D5_ (0.3 g) by semi-preparative HPLC-MS on a C18 Kinetex column (250 × 10 mm, 5 µm; Phenomenex, Utrecht, The Netherlands), using a gradient of H_2_O + 0.1% formic acid (A) and far-UV acetonitrile (B) at 3 mL min^−1^. The gradient program was: (0–5 min) 20%, (20 min) 70% B, (35 min) 95% B, (41 min) 20% B, (41–46 min) 20% B. DAD spectra were acquired over the range 200–450 nm. Mass spectra were recorded in ESI (+) and ESI (−) modes under the following conditions: scan range *m*/*z* 100–1000; capillary voltage 3.00 kV; cone voltage 50 V; extractor voltage 3 V; source temperature 135 °C; desolvation temperature 400 °C; desolvation gas flow 750 L h^−1^; and cone gas flow 50 L h^−1^. The injection volume was 300 µL. Purification of fraction *T*_D7_ by LC-SPE-NMR allowed the isolation of compound 13 (1.3 mg). An Agilent Zorbax SB-phenyl column (4.6 × 250 mm, 5 µm) was used with a mobile phase consisting of water + 0.1% formic acid (A) and acetonitrile (B), and a flow rate of 1 mL min^−1^. The following gradient was used: (0–5 min) 20% B, (15 min) 60% B, (30 min) 96% B, (30–35 min) 96% B, (36 min) 20% B, (36–41 min) 20% B.

#### 2.4.3. Purification of the methanol fraction

A total of 30 g of the methanolic fraction (*T*_M_) was dissolved in 3% methanol (v/v) and subjected to open-column chromatography using Diaion HP-20 resin (450 g). Elution was performed using a stepwise gradient of methanol in water (5, 10, 15, 20, 30, 40, 50, 60, 70, 80, 90 and 100%), with 1 L of eluent applied per step. Twelve fractions (*T*_M1_–*T*_M12_) were collected, concentrated under reduced pressure, and subsequently lyophilized. The antibacterial, antifungal and antiplasmodial activities of all fractions were assessed and the most active fractions were further purified. The purification of fraction (*T*_M3_) (305 mg) by reverse phase flash chromatography using a GraceResolv (12 g) RP18 column eluted with a linear gradient of water (A) and acetonitrile (B) at a flow rate of 3.0 mL min^−1^ yielded three fractions. The gradient was set as follows: (0–30 min) 4% B, (50 min) 50% B, (90 min) 100% B and this condition was maintained for 10 min (90–100 min).

The purification of fraction *T*_M3–2_ by LC-SPE-NMR yielded compound 14 (2.0 mg). The mobile phase consisted of water + 0.1% formic acid (A) and acetonitrile (B) pumped at a flow rate of 1 mL min^−1^. The gradient was set as follows: (0–5 min) 20% B, (15 min) 60% B, (30 min) 96% B, (30–35 min) 96% B, (36 min) 20% B, (36–41 min) 20% B. The peak detected at 280 nm was repeatedly collected on SPE cartridges during 11 consecutive runs. In turn the purification of the fraction *T*_M9_ (0.35 g) by semi-preparative HPLC-MS using a C18 Kinetex column (250 cm × 10.0 mm, particle size, 5 µm) and eluted with a mixture of water (A) and acetonitrile (B) yielded compounds 15 (8.0 mg) and 16 (7.6 mg). The gradient was set as follows: (0–5 min) 20% B, (40 min) 25% B, (50 min) 95% B, (50–55 min) 95% B, with a flow rate of 4.75 mL min^−1^.

Fractions *T*_M10_ and *T*_M12_ were further subjected to semi-preparative HPLC-MS analysis. Indeed, fraction *T*_M10_ (0.2 g) was purified using a gradient of 16% B (0–5 min), 30% B (40 min), and 96% B (50–55 min) at a flow rate of 4.75 mL min^−1^, yielding compounds 17 (5.0 mg) and 18 (24.0 mg). Fraction *T*_M12_ (0.25 g) was purified with a gradient of 20% B (0–5 min), 50% B (60 min), and 95% B (70–75 min) at a flow rate of 3.0 mL min^−1^, affording compound 19 (3.5 mg). All fractions were prepared at a concentration of 20 mg mL^−1^, with injection volumes of 300 µL for *T*_M9_ and *T*_M10_, and 400 µL for *T*_M12_.

### 2.5. LC-ESI-MS

LC-MS analysis was conducted under previously described metabolite profiling conditions, with minor modifications.^19^ The extract was initially dissolved in 80% MeOH (v/v) to 1 mg mL^−1^ and subsequently diluted with water to achieve a final concentration of 0.1 mg mL^−1^. Fraction samples were further diluted to a working concentration of 0.05 mg mL^−1^, whereas purified metabolites were analyzed at 0.01 mg mL^−1^.

### 2.6. Biological evaluation

#### 2.6.1. Antibacterial and antifungal activity

The antimicrobial activity of all fractions and purified metabolites was assessed following the protocols described by Cos *et al.*^20^ and Baldé *et al.*^12^ All samples were tested in triplicate against the following microorganisms: *Staphylococcus aureus* ATCC 6538 (Gram-positive) and *Candida albicans* ATCC 59630 (yeast). Flucytosine and doxycycline were employed as positive controls for *C. albicans* and *S. aureus*, respectively. The half-maximal inhibitory concentrations (IC_50_) of both compounds were determined. These reference compounds are routinely included in the screening platform, and their observed activities were consistent with previously reported values.^12,19,20^

#### 2.6.2. Antiplasmodial and cytotoxicity assays

Antiplasmodial and cytotoxic activities were determined following established procedures described by Cos *et al.*^20^ and Tuenter *et al.*^21^ Fractions and purified metabolites were screened against the chloroquine-resistant strain *Plasmodium falciparum* K1 strain using the lactate dehydrogenase assay. Compounds exhibiting the highest activity were analyzed in triplicate, and data are reported as mean ± standard deviation (SD). Cytotoxicity was evaluated in MRC-5_SV2_ human foetal lung fibroblasts cells (ECACC 84100401) with tamoxifen as the positive control, whereas chloroquine served as the reference antiplasmodial agent. Both standards are part of the routine quality controls for the screening platform, and their potencies fell within the expected performance ranges documented previously.^12,19,20^

#### 2.6.3. Physicochemical and spectral data of isolated compounds

The NMR data of all isolated compounds are provided in the SI.

## 3 Results and discussion

Preliminary biological studies have shown that extracts from the leaves of *T. alnifolia* were active against *P. falciparum* and *C. albicans*. In this study, a classical activity-guided fractionation approach was used in order to characterize the active metabolites of *T. alnifolia* leaves.

### 3.1. Purification and identification of isolated compounds

The fractions exhibiting the strongest activities (*n*-hexane, dichloromethane, and methanol) against *P. falciparum* and *C. albicans* (Table 1) were subjected to a multistep purification workflow comprising normal and reversed-phase flash chromatography, semi-preparative HPLC-MS, and LC-SPE-NMR. This process yielded a total of 19 distinct metabolites. Of these, five were isolated from the *n*-hexane fraction, eight from the dichloromethane fraction, and six from the methanol fraction. Structural characterization of all isolated metabolites was performed using HR-ESI-MS in conjunction with detailed NMR spectroscopy, employing both 1D (^1^H, ^13^C, DEPT-135, DEPT-90) and 2D (COSY, HSQC, HMBC) experiments. The spectroscopic data obtained were in full agreement with previously published assignments, allowing the identification of squalene (1),^22,23^ cycloart-24-en-3β-yl α-linolenate (2),^24^ α-tocopherol (3),^25,26^*trans*-pentamethyl-icosa-tetraene (4),^27^ 3-β-hydroxy-olean-12-ene-heptadecanoate (5), phytol (6),^28^ isophytol (7),^29^ (1, 2)-bis-nor-phytone (8),^30^ pheophorbide-*a* methyl ester (9),^31,32^ pheophorbide-*b* methyl ester (10),^33^ stigma-5-en-3-*O*-β-glucoside (11),^34^ vanillic acid (12),^35^ abscisic acid (13),^36,37^ gallic acid (14),^38^ myricetin-3-*O*-rhamnopyranoside (15),^39^ quercetin-3-*O*-rhamnopyranoside (16),^40^ myricetin-3′,5′-dimethylether-3-*O*-galactopyranoside (17),^41^ quercetin-3-*O*-β-d-galactopyranoside (18)^42^ and epicatechin-3-galloylester (19).^43^ Apart from gallic acid (14), all compounds isolated in this study are reported for the first time in *T. alnifolia*.

**Table 1:** *In vitro* antimicrobial, antiplasmodial and cytotoxic activity of extracts and fractions from *Tetracera alnifolia* leaves

Plant name	Antimicrobial activity			Antiplasmodial activity (IC_50_, µg mL^−1^)	Cytotoxicity (CC_50_, µg mL^−1^)	Selectivity index
	*S. aureus*	*E. coli*	*C. albicans*	*Pf-K1*	*MRC-5*_SV2_	*MRC-5*_SV2_/*PfK1*
*T*_E_	>64.0	>64.0	>64.0	**2.2**	32.7	14.8
*T*_D_	>64.0	>64.0	6.8	1.81	3.6	2.0
*T*_M_	>64.0	>64.0	18.3	46.4	>64.0	>1.3
*T*_M1_	>64.0	>64.0	>64.0	34.5	>64.0	>1.8
*T*_M2_	>64.0	>64.0	>64.0	7.1	>64.0	8.92
*T*_M3_	>64.0	>64.0	>64.0	1.3	31.0	23.4
*T*_M4_	>64.0	>64.0	>64.0	8.8	30.4	3.4
*T*_M5_	>64.0	>64.0	>64.0	19.6	>64.0	>3.25
*T*_M6_	>64.0	>64.0	>64.0	25.2	30.1	1.2
*T*_M7_	>64.0	>64.0	3.6	23.8	28.5	1.19
*T*_M8_	>64.0	>64.0	1.7	16.0	26.3	1.6
*T*_M9_	>64.0	>64.0	1.6	18.8	29.4	1.5
*T*_M10_	>64.0	>64.0	5.9 ± 4.7	4.3 ± 0.8	>64.0	>13.0
*T*_M11_	>64.0	>64.0	25.2	18.66	>64.0	>3.4
*T*_M12_	>64.0	>64.0	3.6 ± 3.0	37.07	>64.0	>1.7
Chloroquine				0.2 ± 0.1 µM		
Doxycycline	0.3 ± 0.2 µM	0.6 ± 0.3 µM				
Flucytosine			0.70 ± 0.01 µM			
Tamoxifen					10.0 ± 1.5 µM	

### 3.2. Antiplasmodial activity of fractions and isolated compounds

The antiplasmodial activity of most of these compounds has been evaluated and the highest activity was obtained for pheophorbide-*b* methyl ester (1.0 ± 0.7 µM) (10), (1,2)-bis-nor-phytone (2.0 µM) (8), isophytol (4.0 µM) (7), pheophorbide-*a* methyl ester (2.8 ± 1.2 µM) (9), epicatechin-3-galloylester (5.5 ± 2.1) (19) and phytol (6.9 ± 2.4 µM) (6) (Table 1). Apart from myricetin-3-*O*-rhamnopyranoside (15) (14.3 µM), α-tocopherol (3) (13.5 µM), cycloart-24-en-3β-yl α-linolenate (2) (25.0 µM), which showed weak antiplasmodial activity, the other tested compounds displayed no activity.

Pheophorbide derivatives are chlorophyll degradation products formed through the removal of the phytol side chain and the central magnesium ion from the tetrapyrrole macrocycle of chlorophyll *a*.^44,45^ A qualitative assessment of the structure–activity relationships within this class of chlorophyll-derived metabolites highlights the influence of functional group modifications and oxidative transformations on antiplasmodial activity. Pheophorbide-*a* methyl ester (9) exhibited moderate activity against *P. falciparum* (IC_50_ = 2.8 ± 1.2 µM), consistent with previously reported data.^46^ Its potency was slightly greater than that of pheophorbide-*a* (IC_50_ = 6.7 µg mL^−1^) and the C-13 hydroxylated analogue (IC_50_ = 5.1 µg mL^−1^), previously isolated from *Mezoneuron benthamianum*.^44^ Taken together, these data suggest that hydroxylation at C-13, in combination with esterification of the C-17 carboxyl group, enhance the antiplasmodial activity of pheophorbide-*a*. Furthermore, the presence of an aldehyde moiety at C-7, as observed in pheophorbide-*b* methyl ester (10), resulted in a marked improvement in antiplasmodial activity (IC_50_ = 1.4 ± 0.4 µM). However, this structural modification was accompanied by a significant increase in cytotoxicity (CC_50_ = 0.7 µM), indicating that the aldehyde moiety may also augment nonspecific reactivity toward mammalian cells. Although pheophorbide-*b* methyl ester (10) and pheophorbide-*a* methyl ester (9) have previously been isolated from various plant species, including *Piper penangense*, *Clerodendrum* spp., and *Garuga pinnata*, this study constitutes the first report of their presence in *T. alnifolia*.^33,47^ Pheophorbide derivatives have attracted considerable interest due to their application in malaria vector control and their ability to eliminate *P. falciparum* through photosensitization.^48,49^ In particular, synthetic lipophilic pheophorbide analogues have demonstrated potent antiplasmodial activity upon exposure to red light, highlighting the therapeutic potential of photodynamic activation. Moreover, earlier studies have proposed pheophorbide-mediated photosensitization as a promising strategy for the inactivation of transfusion-transmissible parasites and viruses in blood products, further underscoring the biomedical relevance of this compound class.^50^

Phytol (6) and two of its derivatives (isophytol and (1,2)-bis-nor-phytone) were among the most active compounds identified in this study. Phytol, a diterpenoid alcohol that forms the phytyl side chain of chlorophyll, is ubiquitous across photosynthetic organisms.^51^ Its antiplasmodial activity has been previously reported against the chloroquine-sensitive *P. falciparum* D10 strain (IC_50_ = 5.60 ± 0.18 µg mL^−1^), and bioassay-guided fractionation study has likewise identified phytol as a potent constituent against both chloroquine-resistant *P. falciparum* PoW (IC_50_ = 8.5 µM) and chloroquine-sensitive Dd2 (IC_50_ = 11.5 µM) strains.^52^ Although phytol and its derivatives isophytol (7) and (1,2)-bis-nor-phytone (8) have been reported in other plant species such as *Calotropis procera*,^53^*Glycine hispida*,^54^*Pellia epiphylla*, *Microglossa pyrifolia*,^55^*Brassica oleracea*^56^ and *Strobilanthes crispus*,^57^ to the best of our knowledge, this is the first report of their occurrence in *T. alnifolia*, and that antiplasmodial activity was reported for isophytol (7) and (1,2)-bis-nor-phytone (8). Isophytol is generally considered as a relatively short lived intermediate in the degradation of the chlorophyll phytyl side chain and has been reported in the essential oil of several terrestrial plants.^58^

A clear structure–activity trend is evident within this series: phytol displayed moderate activity (IC_50_ = 6.9 ± 2.4 µM), whereas isophytol, differing only in the position of the double bond, demonstrated improved potency (IC_50_ = 4.0 µM). Further oxidative modification to generate (1,2)-bis-nor-phytone yielded the most active compound in this subset (IC_50_ = 2.0 µM). These results indicate that both the location of unsaturation and the degree of oxidative transformation along the diterpenoid chain play critical roles in modulating biological activity. In particular, progressive oxidation and carbon-chain truncation may enhance the antiplasmodial effects. Collectively, these results suggest that electrophilic modifications on the porphyrin core, together with oxidative tailoring of the phytol side chain are key drivers of antiplasmodial activity within this chlorophyll-derived compound class.

In order to determine the specificity of the compounds' antiplasmodial activity, their cytotoxicity on MRC-5_SV2_ cells was evaluated. Apart from pheophorbide-*a* methyl ester (SI 1.7), pheophorbide-*b* methyl ester (SI 1.6) and phytol (SI 1.3), all other compounds did not show any cytotoxicity up to the highest concentration tested (64 µM) (Table 2). Several studies have previously reported the cytotoxicity of pheophorbide derivatives. Indeed, several of the photosensitizers that were investigated for clinical photodynamic therapy (PDT) of cancer are constituents from plants. Some examples including degradation derivatives of chlorophylls, such as pheophorbides, which are present in green leaves and are well-studied photosensitizers in photodynamic cancer therapy.^47^ Moreover, most of the photosensitizers clinically approved or in clinical trials are derived from naturally occurring structures based on cyclic tetrapyrroles, such as chlorophyll-based compounds from higher plants.^59^

**Table 2:** *In vitro* antimicrobial, antiplasmodial and cytotoxic activity of isolated compounds from *Tetracera alnifolia* leaves

Compound names	Structures	Molecular formula	Exact mass	Antimicrobial activity		Antiplasmodial activity (IC_50_, µM)	Cytotoxicity (CC_50_, µM)	Selectivity index
				*S. aureus*	*C. albicans*	*Pf-K1*	*MRC-5*_SV2_	*MRC-5*_SV2_/*PfK1*
Squalene (1)	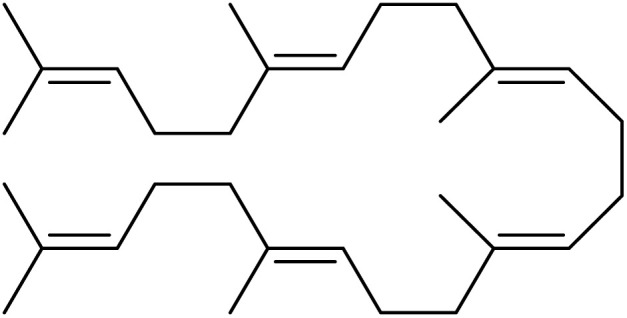	C_30_H_50_	410.3912	>64.0	>64.0	Nd	20.6	Nd
Cycloart-24-en-3β-yl α-linolenate (2)	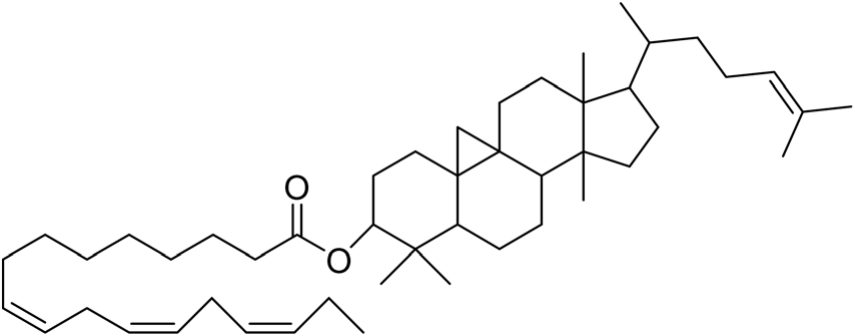	C_48_H_78_O_2_	686.6001	>64.0	>64.0	25.0	>64.0	>2.4
α-Tocopherol (3)	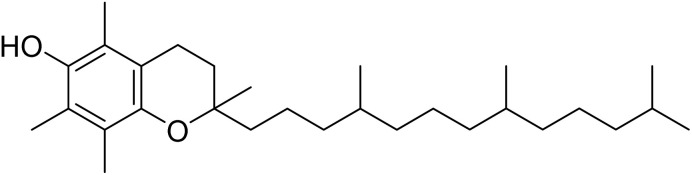	C_29_H_50_O_2_	430.3810	>64.0	>64.0	13.5	>64.0	>4.4
*trans*-Pentamethyl-icosa-tetraene (4)	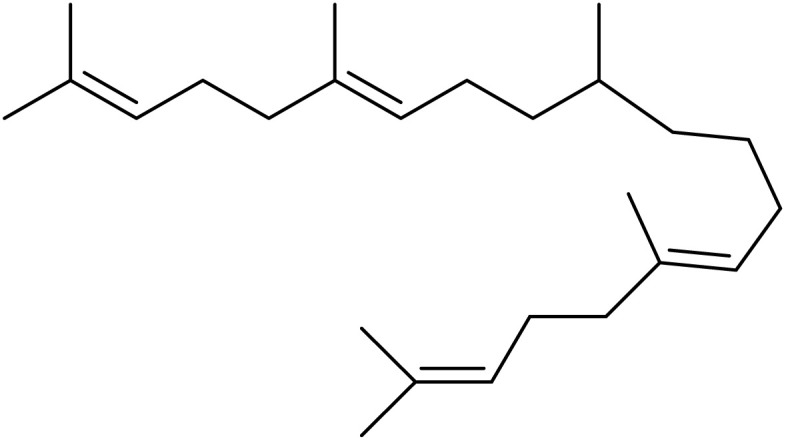	C_25_H_44_	344.3443	Nd	Nd	Nd	Nd	Nd
3-β-Hydroxy-olean-12-ene-heptadecanoate (5)	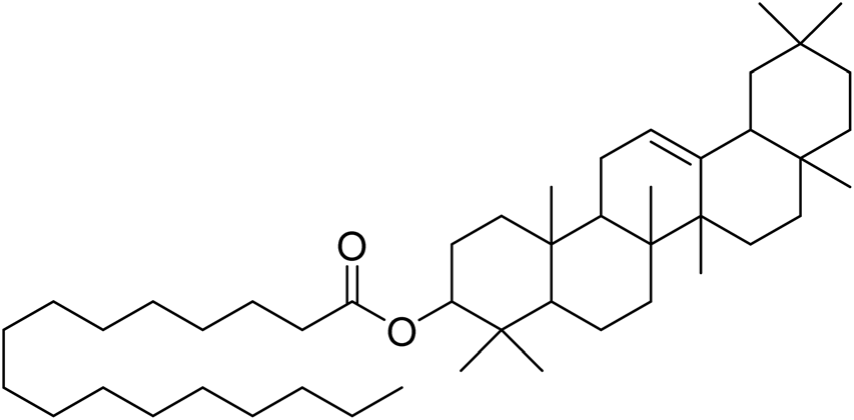	C_47_H_82_O_2_	678.6314	>64.0	>64.0	>64.0	>64.0	>64.0
Phytol (6)	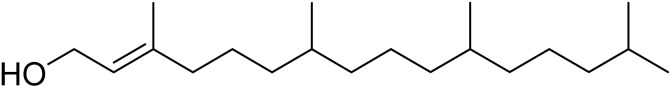	C_20_H_40_O	296.3079	>64.0	>64.0	6.9 ± 2.4	8.0	1.3
Isophytol (7)	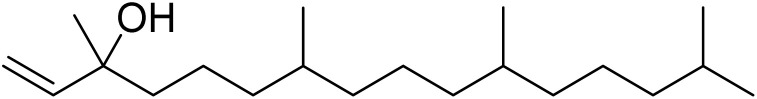	C_20_H_40_O	296.3079	>64.0	>64.0	4.0	>64.0	>64.0
(1,2)-Bis-nor-phytone (8)	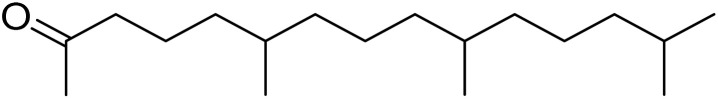	C_18_H_36_O	268.2766	>64.0	>64.0	2.0	>64.0	>64.0
Pheophorbide-*a* methyl ester (9)	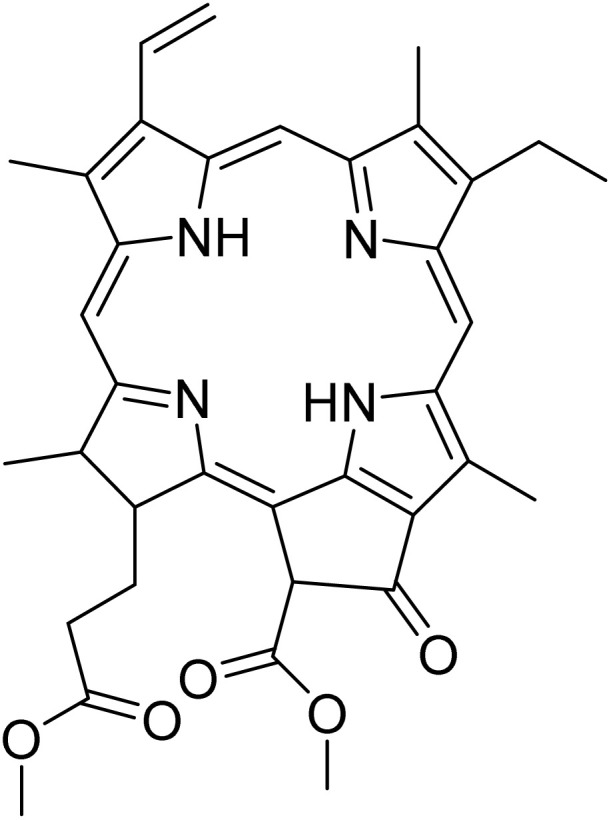	C_36_H_38_N_4_O_5_	606.2842	64.00	>64.0	2.8 ± 1.2	7.5	1.7
Pheophorbide-*b* methyl ester (10)	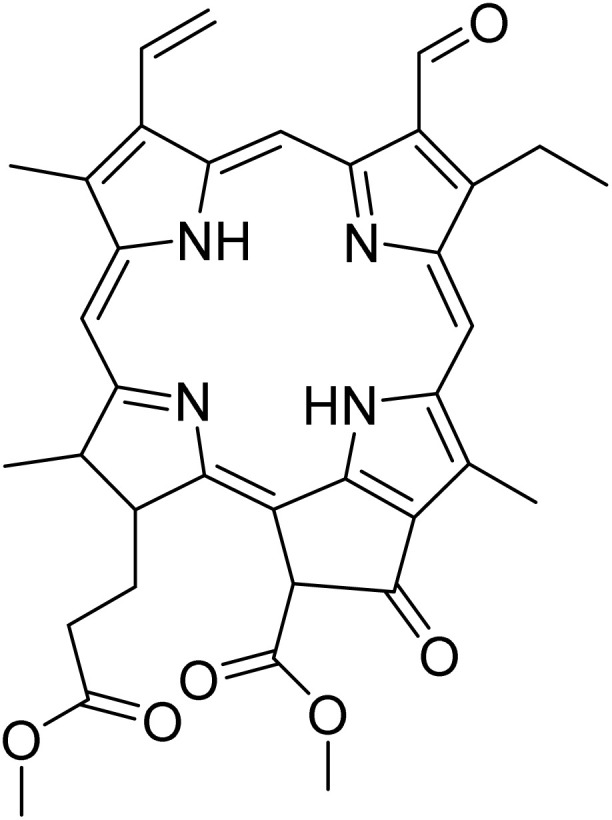	C_36_H_36_N_4_O_6_	620.2635	>64.0	>64.0	1.4 ± 0.4	0.7	0.5
Stigma-5-en-3-*O*-β-glucoside (11)	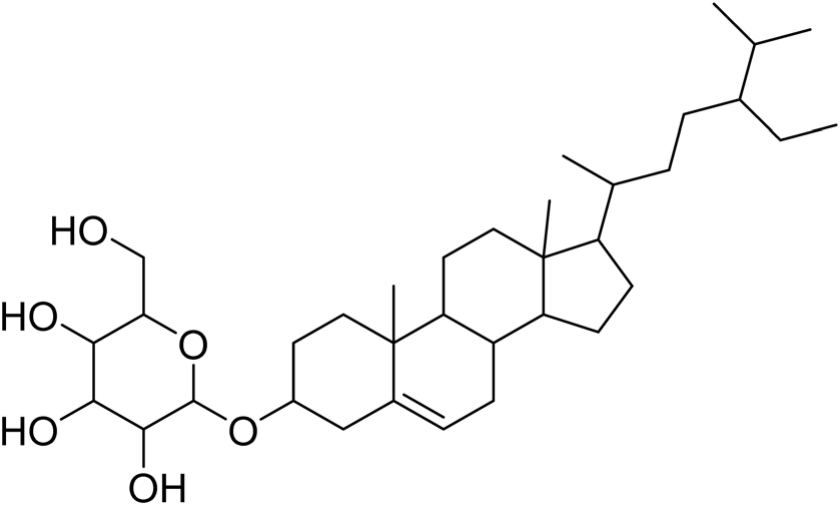	C_35_H_60_O_6_	576.4390	>64.0	>64.0	>64.0	>64.0	Nd
Vanillic acid (12)	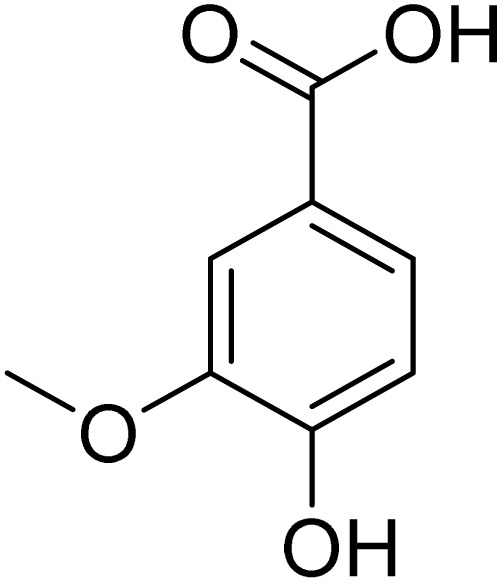	C_8_H_8_O_4_	168.0423	>64.0	>64.0	>64.0	>64.0	Nd
Abscisic acid (13)	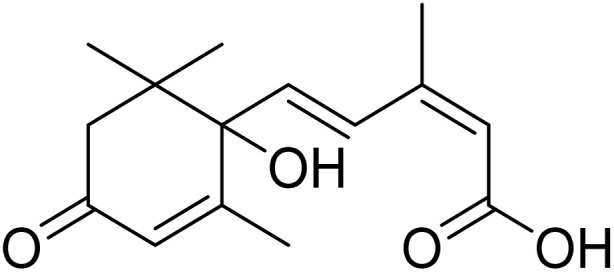	C_15_H_20_O_4_	264.1362	>64.0	>64.0	>64.0	>64.0	>64.0
Gallic acid (14)	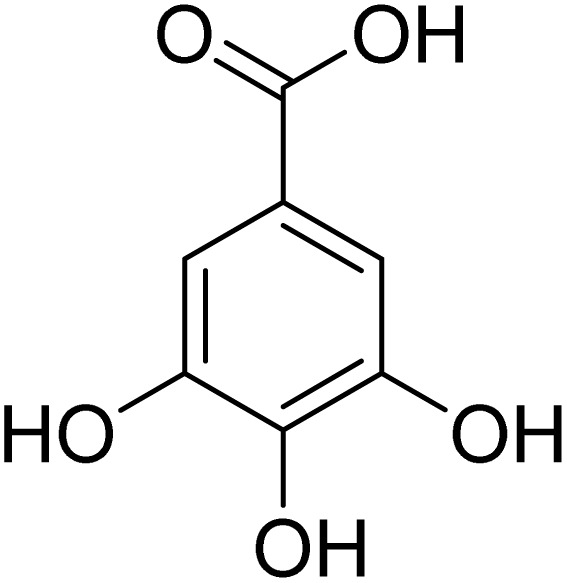	C_7_H_6_O_5_	170.0215	32.46	>64.0	Nd	8.06	Nd
Myricetin-3-*O*-rhamnopyranoside (15)	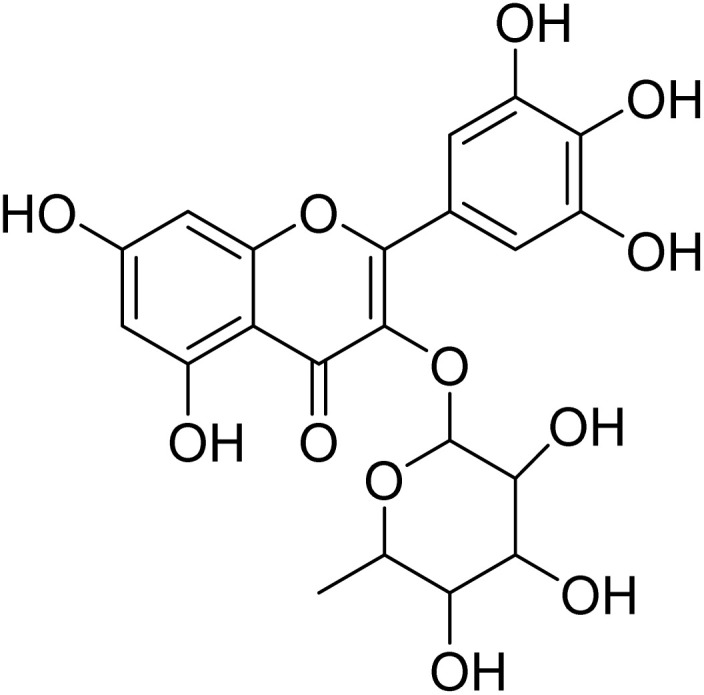	C_21_H_20_O_12_	464.3790	>64.0	>64.0	14.3	>64.0	Nd
Quercetin-3-*O*-rhamnopyranoside (16)	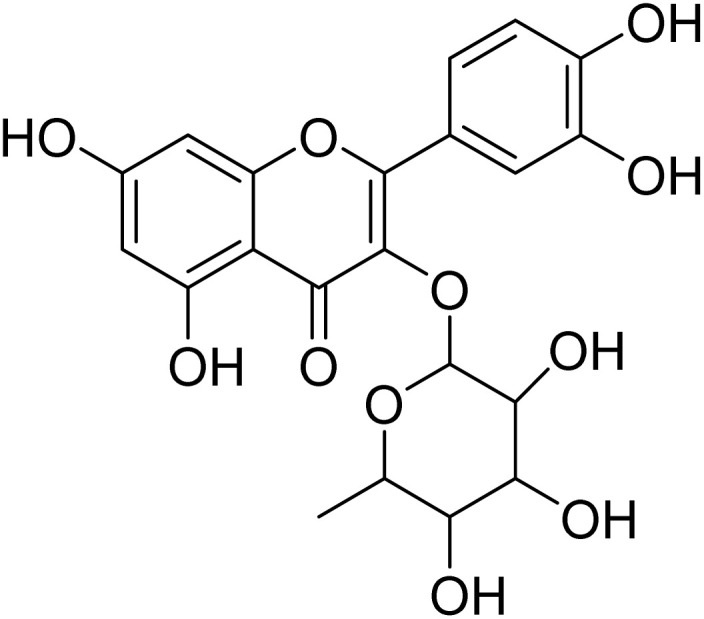	C_21_H_20_O_11_	448.1006	>64.0	>64.0	>64.0	>64.0	Nd
Myricetin-3′,5′-dimethylether-3-*O*-galactopyranoside (17)	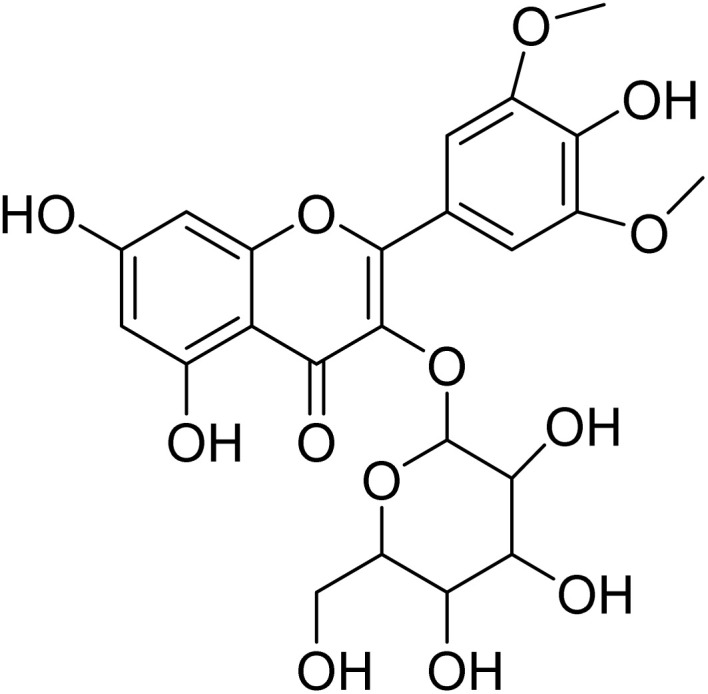	C_23_H_24_O_13_	508.1217	>64.0	>64.0	>64.0	>64.0	Nd
Quercetin-3-*O*-galactopyranoside (18)	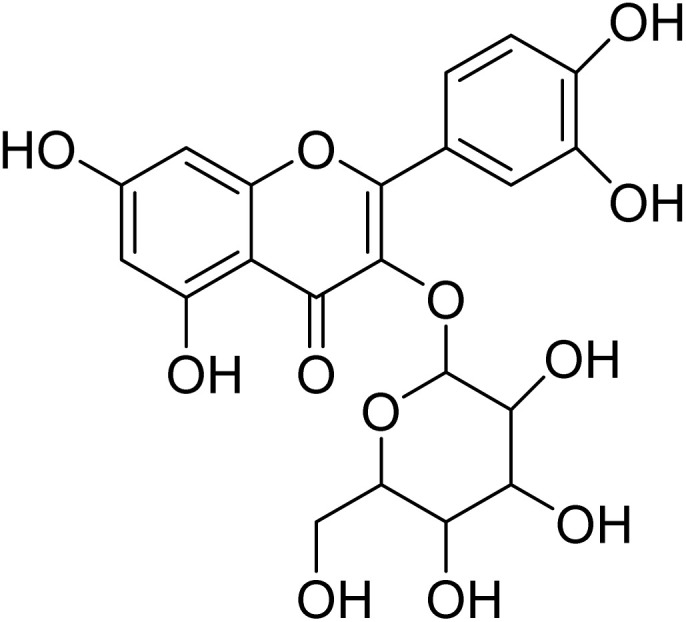	C_21_H_20_O_12_	464.0955	>64	>64	>64	>64	Nd
Epicatechin-3-galloylester (19)	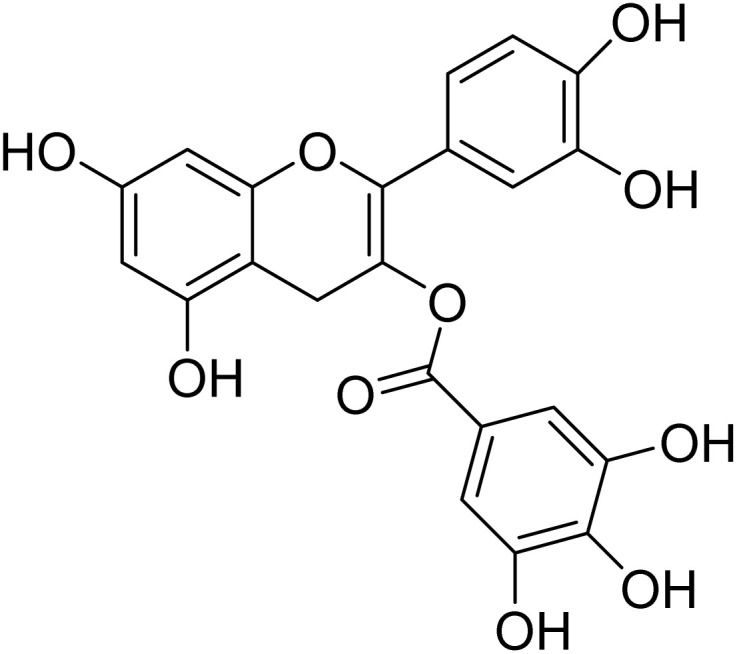	C_22_H_16_O_10_	440.0743	>64	>64	5.5 ± 2.1	>64	>11.6
Chloroquine						0.2 ± 0.1 µM		
Doxycycline				0.3 ± 0.2 µM				
Flucytosine					0.70 ± 0.01 µM			
Tamoxifen							10.0 ± 1.5 µM	

Among the isolated phenolic compounds, epicatechin-3-galloyl ester (19) (IC_50_ = 5.5 ± 2.1 µM) and myricetin-3-*O*-rhamnopyranoside (4.5 µM) were the most active. Activity across this series appears to be governed by the number of free hydroxyl groups on the B-ring and the presence of a galloyl moiety. It is noteworthy that epicatechin-3-galloyl ester (19) has previously been isolated from *Camellia sinensis*^60^ and *Sclerocarya birrea*.^43^ Its antiplasmodial activity has also been documented, with IC_50_ values of 10.8 µM against the chloroquine-sensitive *P. falciparum* 3D7 strain and 7.2 µM against the chloroquine-resistant FCR-1/FVO strain.^60^

Myricetin-3-*O*-rhamnopyranoside (15) has shown moderate antiplasmodial activity (IC_50_ 14.3 µM), while quercetin-3-*O*-rhamnopyranoside (16) was completely inactive at the highest concentration tested (IC_50_ 64.0 µM). These two compounds are structurally related. However, their difference is located in position C-5′ of their aglycone moiety where myricetin bears an additional substitution with a hydroxyl group. It appears that addition of the hydroxyl group in position C-5′, as in myricetin-3-*O*-rhamnoside, could have a strong influence on the antiplasmodial activity. In contrast to our result, a previous study carried out by Ganesh *et al.*^61^ revealed that quercetin-3-*O*-rhamnoside demonstrated weak activity against fresh *P. falciparum* field isolates from Bangladesh (IC_50_ 20.4 ± 14.5 µM), *P. falciparum* chloroquine-sensitive 3D7 (IC_50_ 35.4 ± 6.4) and *P. falciparum* chloroquine-resistant K1 (IC_50_ 12 ± 27). Quercetin and myricetin can be found both in their free form (aglycon) and in their glycosylated form (heteroside) in several plant organs. Previous studies revealed that the antiplasmodial activity found for myricetin-3-*O*-rhamnopyranoside and quercetin-3-*O*-rhamnopyranoside were much lower than the activity of their corresponding aglycons myricetin (IC_50_ 8.9 µM) and quercetin (IC_50_ 12.9 µM).^61,62^ To date, quercetin and myricetin have been found to exhibit strong inhibitory activity against three important enzymes (FabG, FabZ, and FabI) involved in the fatty acid biosynthesis of *P. falciparum* and these flavonoids showed *in vitro* activity against chloroquine-sensitive (NF54) and chloroquine-resistant (K1) *P. falciparum* strains. Moreover, myricetin and quercetin inhibited the intraerythrocytic growth of the chloroquine-sensitive (3D7) and chloroquine-resistant (7G8) strains of *P. falciparum*.^63^

### 3.3. Antibacterial and antifungal activities of fractions and isolated compounds

The fractions and isolated compounds were also tested against *S. aureus* and *C. albicans*. The methanol fractions (*T*_M_) and its subfractions *T*_M7_ to *T*_M12_ were active against *C. albicans* (Table 1). Apart from gallic acid (14), which showed weak activity against *S. aureus*, none of the other compounds showed antimicrobial activity. The antibacterial activity of gallic acid against *S. aureus* in suspension (MIC 2 mg mL^−1^) and in biofilms (MIC 4 mg mL^−1^) has previously been reported.^64^ Furthermore, a study carried out by Chanwitheesuk *et al.*^65^ described the effectiveness of gallic acid against *S. aureus* (MIC 1250 µg mL^−1^). In contrast to our results, a previous study carried out by Motlhatlego *et al.*^66^ showed that myricetin-3-*O*-rhamnoside isolated from *Newtonia buchananii* demonstrated antibacterial activity against *S. aureus* (MIC 62.5 µg mL^−1^). Previous studies have suggested that phytol exhibits broad-spectrum antimicrobial effects.^51^ In particular, Inoue *et al.*^67^ reported strong antimicrobial activity of this compound against *S. aureus* (MIC 0.15 µg mL^−1^).

## 4 Conclusion

This study provide experimental support for the traditional application of *T. alnifolia* in treating infectious diseases, particularly malaria. Guided by bioassay results, we isolated and characterized active constituents from the leaves, thereby substantiating its ethnopharmacological basis. The *n*-hexane, dichloromethane, and methanol extracts were active against *P. falciparum*, and subsequent fractionation afforded 19 metabolites, the majority of which are reported from this species for the first time. The most potent antiplasmodial activity was obtained for pheophorbide-*b* methyl ester (10), (1,2)-bis-nor-phytone (8), isophytol (7), pheophorbide-*a* methyl ester (9) and phytol (6). In contrast, compounds such as myricetin-3-*O*-rhamnopyranoside (15), α-tocopherol (3) and cycloart-24-en-3β-yl α-linolenate (2) showed comparatively lower activity. Notably, pheophorbide-*b* methyl ester (10), pheophorbide-*a* methyl ester (9) and phytol (6) also demonstrated significant cytotoxicity. These findings may contribute to a better understanding of the antiplasmodial properties observed in the leaf extract of *T. alnifolia*.

## Author contributions

M. A. Baldé: conceptualization, investigation, formal analysis, data analysis, writing original draft. M. S. Traoré: data analysis, sample identification, writing review & editing. M. S. T. Diallo: sample collection, data analysis. An Matheeussen: methodology, data curation. C. Aïssata: methodology, data curation, data visualization. P. Cos: methodology, antimicrobial assay, data validation. L. Maes: methodology, antiplasmodial assay, data validation. A. O. Baldé: sample collection and preparation of raw extract. M. K. Camara: data curation and data analysis. A. M. Balde: funding acquisition, supervision, writing review and editing. E. Tuenter: data analysis and visualization, writing review and editing. K. Foubert: conceptualization, supervision, writing review and editing.

## Conflicts of interest

There are no conflicts to declare.

## Supplementary Material

RA-016-D5RA09498D-s001

## Data Availability

The data supporting this article are included within the supplementary information (SI). Supplementary information: NMR data. See DOI: https://doi.org/10.1039/d5ra09498d.
