# Supplementary material for: Isolation and characterization of antiplasmodial and antimicrobial compounds from Tetracera alnifolia using a bioassay-guided approach
Source: RSC Adv. 2026 Feb 23;16(12):10635–47. doi: 10.1039/d5ra09498d (PMC12926867; doi:10.1039/d5ra09498d)
Supplement: RA-016-D5RA09498D-s001 [file RA-016-D5RA09498D-s001.pdf]

Supplementary Information (SI) for RSC Advances.
This journal is © The Royal Society of Chemistry 2026

Supplementary information

Isolation and Characterization of Antiplasmodial and Antimicrobial Compounds from *Tetracera alnifolia* Using a Bioassay-Guided Approach

Mamadou Aliou Baldé[a*], Mohamed Sahar Traoré[a,d], Mamadou Saliou Telly Diallo[a], An Matheeussen[c], Camara Aïssata[a], Paul Cos[c], Louis Maes[c], Alpha Oumar Baldé[a], Mohamed Kerfala Camara[a], Aliou Mamadou Balde[a], Emmy Tuenter[b], Kenn Foubert[b]

[a]*Department of Pharmaceutical and Biological Sciences, University Gamal Abdel Nasser of Conakry, Guinea;*
[b]*Natural Products & Food Research and Analysis - Pharmaceutical Technology (NatuRAPT), Department of Pharmaceutical Sciences, University of Antwerp, Universiteitsplein 1, B-2610 Antwerp, Belgium*
[c]*Laboratory for Microbiology, Parasitology and Hygiene (LMPH), Faculty of Pharmaceutical, Biomedical and Veterinary Sciences, University of Antwerp, Universiteitsplein 1, B-2610 Antwerp, Belgium*
[d]*Research Institute of Applied Biology of Guinea (IRBAG), Kindia, Guinea*

***Corresponding Author:** Mamadou Aliou Baldé (email: *baldem@ufl.edu*)

This file contains additional figures, NMR spectra of isolated compouds supporting the main manuscript.

1.*Physicochemical and spectral data of isolated compounds*........3
Figure S.1. ^{1}H NMR spectrum ($CDCl_3$, 400 MHz) of squalene **(1)**........9
Figure S.2. ^{13}C NMR spectrum ($CDCl_3$, 100 MHz) of squalene **(1)**........9
Figure S.3. ^{1}H NMR spectrum ($CDCl_3$, 400 MHz) of cycloart-24-en-3β-yl α-linolenate **(2)**........10
Figure S.4. ^{13}C NMR spectrum ($CDCl_3$, 100 MHz) of cycloart-24-en-3β-yl α-linolenate **(2)**........10
Figure 6.5. COSY spectrum ($CDCl_3$) of Cycloart-24-en-3β-yl α-linolenate **(2)**........11
Figure S.6. COSY spectrum ($CDCl_3$) of Cycloart-24-en-3β-yl α-linolenate **(2)**........11
Figure S.7. COSY spectrum ($CDCl_3$) of Cycloart-24-en-3β-yl α-linolenate **(2)**........12
Figure S.8. ^{1}H NMR spectrum (DMSO, 400 MHz) of α-Tocopherol **(3)**........12
Figure S.9. ^{13}C NMR spectrum (DMSO, 100 MHz) of α-Tocopherol **(3)**........13
Figure S.10. COSY spectrum (CDCl3) of α-Tocopherol **(3)**........13
Figure S.11. HSQC spectrum (CDCl3) of α-Tocopherol **(3)**........14
Figure S.12. HMBC spectrum (CDCl3) of α-Tocopherol **(3)**........14
Figure S.13. ^{1}H NMR spectrum ($CDCl_3$, 400 MHz) of Trans-pentamethyl-Icosa-tetraene **(4)**........15

Figure S.14. ^{13}C NMR spectrum ($CDCl_3$, 100 MHz) of Trans-pentamethyl-Icosa-tetraene **(4)**............................15

Figure S.15. ^{1}H NMR spectrum ($CDCl_3$, 400 MHz) of 3-β-hydroxy-olean-12-ene-heptadecanoate **(5)**................16

Figure S.16. ^{13}C NMR spectrum ($CDCl_3$, 100 MHz) of 3-β-hydroxy-olean-12-ene-heptadecanoate **(5)**................16

Figure S.17. COSY spectrum ($CDCl_3$) of 3-β-hydroxy-olean-12-ene-heptadecanoate **(5)**......................................17

Figure S.18. HSQC spectrum ($CDCl_3$) of 3-β-hydroxy-olean-12-ene-heptadecanoate **(5)**......................................17

Figure S.19. HMBC spectrum ($CDCl_3$) of 3-β-hydroxy-olean-12-ene-heptadecanoate **(5)**....................................18

Figure S.20. ^{13}C NMR spectrum ($CDCl_3$, 100 MHz) of Phytol **(6)**...18

Figure S.21. ^{1}H NMR spectrum ($CDCl_3$, 400 MHz) of Isophytol **(7)** 19

Figure S.22. ^{13}C NMR spectrum ($CDCl_3$, 100 MHz) of Isophytol **(7).** 19

Figure S.23. ^{1}H NMR spectrum ($CDCl_3$, 400 MHz) of (1,2)-Bis-nor-phytone **(8)** 20

Figure S.24. ^{13}C NMR spectrum ($CDCl_3$, 100 MHz) of (1,2)-Bis-nor-phytone **(8)**...20

Figure S.25. ^{1}H NMR spectrum ($CDCl_3$, 400 MHz) of Pheophorbide-A methyl ester **(9)..** 21

Figure S.26. ^{13}C NMR spectrum ($CDCl_3$, 100 MHz) of Pheophorbide-A methyl ester **(9)** 21

Figure S.27. COSY spectrum (CDCl3) of Pheophorbide-A methyl ester **(9)** 22

Figure S.28. HSQC spectrum (CDCl3) of Pheophorbide-A methyl ester **(9)** 22

Figure S.29. HMBC spectrum ($CDCl_3$) of Pheophorbide-A methyl ester **(9)** 23

Figure S.30. ^{1}H NMR spectrum ($CDCl_3$, 400 MHz) of Pheophorbide-B methyl ester **(10)** 23

Figure S.31. ^{13}C NMR spectrum ($CDCl_3$, 100 MHz) of Pheophorbide-B methyl ester **(10)** 24

Figure S.32. COSY spectrum ($CDCl_3$) of Pheophorbide-B methyl ester **(10)** 24

Figure S.33. HSQC spectrum ($CDCl_3$) of Pheophorbide-B methyl ester **(10)** 25

Figure S.34. HMBC spectrum ($CDCl_3$) of Pheophorbide-B methyl ester **(10)** 25

Figure S.35. 1H NMR spectrum (DMSO, 400 MHz) of Stigma-5-en-3-O-β-glucoside **(11)** 26

Figure S.36. ^{13}C NMR spectrum (DMSO, 100 MHz) of Stigma-5-en-3-*O*-β-glucoside **(11)** 26

Figure S.37. ^{1}H NMR spectrum (DMSO, 400 MHz) of Vannilic acid **(12)** 27

Figure S.38. ^{13}C NMR spectrum (DMSO, 100 MHz) of Vannilic acid **(12)** 27

Figure S.39. ^{1}H NMR spectrum (CD_3CN, 400 MHz) of Abscisic acid **(13)** 28

Figure S.40. COSY spectrum (CD_3CN) of Abscisic acid **(13)** 28

Figure S.41. HSQC spectrum (CD_3CN) of Abscisic acid **(13)** 29

Figure S.42. HMBC spectrum (CD_3CN) of Abscisic acid **(13)** 29

Figure S.43. ^{1}H NMR spectrum (CD_3OD, 400 MHz) of Gallic acid **(14)** 30

Figure S.44. ^{13}C NMR spectrum (CD_3OD, 100 MHz) of Gallic acid **(14)** 30

Figure S.45. ^{1}H NMR spectrum (DMSO, 400 MHz) of Myricetin-3-*O*- rhamnoside **(15)** 31

Figure S.46. ^{13}C NMR spectrum (DMSO, 100 MHz) of Myricetin-3-*O*- rhamnoside **(15)** 31
Figure S.47. ^{1}H NMR spectrum (DMSO, 400 MHz) of Quercetin-3-*O*- rhamnoside **(16)** 32
Figure S.48. ^{13}C NMR spectrum (DMSO, 100 MHz) of Quercetin-3-*O*- rhamnoside **(16)** 32
Figure S.49. ^{1}H NMR spectrum (DMSO, 400 MHz) Myricetin-3',5'dimethylether-3*O*-Galactopyranoside **(17)** 33
Figure S.50. COSY spectrum (DMSO) Myricetin-3',5'dimethylether-3-*O*-Galactopyranoside **(17)** 33
Figure S.51. HSQC spectrum (DMSO) Myricetin-3',5'dimethyl-ether-3-*O*-Galactopyranoside **(17)** 34
Figure S.52. HMBC spectrum (DMSO) Myricetin-3',5'dimethyl-ether-3-*O*-Galactopyranoside **(17)** 34
Figure S.53. ^{1}H NMR spectrum (DMSO, 400 MHz) of Quercetin-3-*O*-galactopyranoside **(18)** 35
Figure S.54. COSY spectrum (DMSO) of Quercetin-3-*O*-galactopyranoside **(18)** 35
Figure S.55. HSQC spectrum (DMSO) of Quercetin-3-*O*-galactopyranoside **(18)** 36
Figure S.56. HMBC spectrum (DMSO) of Quercetin-3-*O*-galactopyranoside **(18)** 36
Figure S.57. ^{1}H NMR spectrum (DMSO, 400 MHz) of Epicatechin-3-galloylester **(19)** 37
Figure S.58. COSY spectrum (DMSO) of Epicatechin-3-galloylester **(19)** 37
Figure S.59. HSQC spectrum (DMSO) of Epicatechin-3-galloylester **(19)** 38
Figure S.60. HMBC spectrum (DMSO) of Epicatechin-3-galloylester **(19)** 38

1-.Physicochemical and spectral data of isolated compounds

Squalene **(1).** Colorless oil; ^{1}H NMR (CDCI$_3$ 400 MHz): δ_H 1.59 (s, Me-25, Me-26, Me-27, Me-28, Me-29 and Me-30), 1.66 (s, Me-1 and Me-24), 5.06 (m, H-3 and H-20), 5.08 (m, H-7 and H-18), 5.13 (m, H-13 and H-14). ^{13}C NMR (CDCl$_3$, 100 MHz): δ_C 25.9 (C-1), 131.4 (C-2), 124.4 (C-3), 26.9 (C-4), 40.0 (C-5), 135.1 (C-6), 124.5 (C-7), 26.9 (C-8), 39.9 (C-9), 135.2 (C-10), 124.6 (C-11), 28.5 (C-12), 28.5 (C-13), 124.6 (C-14), 135.2 (C-15), 39.9 (C-16), 26.9 (C-17), 124.5 (C-18), 135.1 (C-19), 40.0 (C-20), 26.8 (C-21), 124.4 (C-22), 131.4 (C-23), 25.9 (C-24), 17.8 (C-25),), 16.2 (C-26), 16.2 (C-27), 16.2 (C-28), 16.2 (C-29), 17.8 (C-30).

Cycloart-24-en-3β-yl α-linolenate **(2).** Colorless gum; ^{1}H-NMR (CDCl$_3$): δ_H 4.54 (dd, *J*=4.6; 11.69 Hz, H-3), 0.54 and 0.31 (d, *J*=4.1 Hz, 2H-19), 1.38 and 1.35 (d, *J*=4.3 Hz, H-5), 1.50 and 1.47 (d, *J*=4.9 Hz, 1H-8), 5.07 (m, H-24). ^{13}C-NMR (CDCl$_3$): δ_C 31.6 (C-1), 26.8 (C-2), 80.2 (C-3), 39.5 (C-4), 47.2 (C-5), 20.9 (C-6), 28.1 (C-7), 47.8 (C-8), 20.1 (C-9), 25.9 (C-10), 25.8 (C-11), 35.6 (C-12), 45.2 (C-13), 48.8 (C-14), 32.8 (C-15), 26.5 (C-16), 52.3 (C-17), 18.0 (C-18), 29.8 (C-19), 35.9 (C-20), 18.3 (C-21), 36.4 (C-22), 24.9 (C-23), 125.3 (C-24),

130.7 (C-25), 25.7 (C-26), 17.6 (C-27), 173.4 (C-1'), 34.8 (C-2'), 25.1 (C-3'), 29.2 (C-4'), 29.1 (C-5'), 29.2 (C-6'), 29.6 (C-7'), 27.2 (C-8'), 130.2 (C-9'), 128.2 (C-10'), 25.6 (C-11') 127.7 (C-12'), 128.2 (C-13'), 25.5 (C-14'), 127.7 (C-15'), 131.9 (C-16'), 20.5 (C-17'), 14.3 (C-18').

α-Tocopherol **(3).** Colorless oil; ^{1}H-NMR ($CDCl_3$): δ_H 2.15 (3H, s, Me-8a), 2.10 (3H, s, Me-5a), 2.10 (3H, s, Me-7a), 1.22 (3H, s, Me-2a), 0.87 (3H, s, Me-22a), 0.87 (3H, s, Me-22b), 0.85 (3H, s, Me-14a), 0.85 (3H, s, Me-18a), 1.52 (1H, m, H-22), 1.39 (2H, m, H-14 and H-118). ^{13}C-NMR ($CDCl_3$): δ_C 74.5 (s, C-2), 31.5 (d, C-3), 20.7 (d, C-4), 121.0 (s, C-5), 11.3 (q, C-5a), 144.5 (s, C-6), 117.3 (s, C-7), 11.8 (q, C-7a), 118.5 (s, C-8), 12.2 (q, C-8a), 122.6 (s, C-9), 145.5 (s, C-10), 39.8 (t, C-11), 21.0 (t, C-12), 37.5 (t, C-13), 32.8 (d, C-14), 19.6 (q, C-14a), 37.3 (t, C-15), 24.4 (t, C-16), 37.5 (t, C-17), 32.8 (d, C-18), 19.7 (q, C-18a), 37.3 (t, C-19), 24.8 (t, C-20), 39.4 (t, C-21), 27.9 (d, C-22a), 22.6 (q, C-22), 22.7 (q, C-22b).

Trans-pentamethyl-icosa-tetraene **(4).** Colorless oil; ^{1}H NMR ($CDCI_{3,}$ 400 MHz): δ_H 1.57 (s, Me-25, Me-26, Me-27, Me-28, Me-29 and Me-30), 1.65 (s, Me-1 and Me-24), 5.05 (m, H-3 and H-20), 5.07 (m, H-7 and H-18), 5.09 (m, H-13 and H-14). ^{13}C NMR ($CDCl_3$, 100 MHz): δ_C 25.9 (C-1), 131.4 (C-2), 124.4 (C-3), 26.9 (C-4), 40.0 (C-5), 135.1 (C-6), 124.5 (C-7), 26.9 (C-8), 39.9 (C-9), 135.2 (C-10), 124.6 (C-11), 28.5 (C-12), 28.5 (C-13), 124.6 (C-14), 135.2 (C-15), 39.9 (C-16), 26.9 (C-17), 124.5 (C-18), 135.1 (C-19), 40.0 (C-20), 26.8 (C-21), 124.4 (C-22), 131.4 (C-23), 25.9 (C-24), 17.8 (C-25),), 16.2 (C-26), 16.2 (C-27), 16.2 (C-28), 16.2 (C-29), 17.8 (C-30).

3-β-Hydroxy-olean-12-ene-heptadecanoate **(5).** Pale yellow oil; ^{1}H-NMR ($CDCl_{3,}$ 400 MHz): δ_H 0.65 (3H, s, Me-26), 0.78 (3H, s, Me-23), 0.78 (3H, s, Me-24), 0.78 (3H, s, Me-28), 0.80 (3H, s, Me-17'), 0.82 (3H, s, Me-30), 0.85 (3H, s, Me-25), 0.85 (3H, s, Me-29), 1.05 (3H, s, Me-27), 5.21 (1H, brs, H-12), 4.43 (1H, m, H-3); ^{13}C-NMR ($CDCl_{3,}$ 100 MHz): δ_C 38.3 (C-1), 23.3 (C-2), 80.8 (C-3), 37.9 (C-4), 55.5 (C-5), 18.4 (C-6), 32.8 (C-7), 39.4 (C-8), 47.7 (C-9), 37.1 (C-10), 23.6 (C-11), 122.5 (C-12), 144.0 (C-13), 48.1 (C-14), 27.9 (C-15), (C-16), 39.7 (C-17), 41.5 (C-18), 46.0 (C-19), 30.8 (C-20), 35.1 (C-21), 38.3 (C-22), 16.98 (C-23), 28.3 (C-24), 15.6 (C-25), 17.0 (C-26), 26.1 (C-27), 28.3 (C-28), 23.8 (C-29), 33.3 (C-30), 173.9 (C-1'), 35.9 (C-2'), 25.4 (C-3'), 29.7 (C-4'), 29.9 (C-5'), 29.9 (C-6'), 29.9 (C-7'), 29.8 (C-8'), 29.8 (C-9'), 29.7 (C-10'), 29.7 (C-11'), 29.7 (C-12'), 29.6 (C-13'), 29.5 (C-14') 29.4 (C-15'), 25.4 (C-16'), 14.4 (C-17').

Phytol **(6)**. Colorless oil. ^{1}H NMR (400 MHz, $CDCl_3$): δ_H 5.36 (1H, td, *J* = 6.9, 1.2 Hz, H-2), 4.11 (2H, d, *J* = 6.9 Hz, H-1), 1.95 (2H, m, H-4), 1.63 (3H, bs, H-20); ^{13}C NMR ($CDCl_3$, 100

MHz): δ_C 59.3 (CH_2, C-1), 123.1 (CH, C-2), 140.1 (C, C-3), 16.1 (CH3, C-5), 39.8 (CH_2, C-6), 25.1 (CH_2, C-7), 36.6 (CH_2, C-8), 32.6 (CH, C-9), 19.7 (CH3, C-10), 37.4 (CH_2, C-11), 24.4 (CH3, C-12), 37.3 (CH_2, C-13), 32.7 (CH, C-14), 19.7 (CH3, C-15), 37.2 (CH_2, C-16), 24.8 (CH_2, C-17), 39.3 (CH_2, C-18), 27.9 (CH, C-19), 22.7 (CH2, C-21), 22.6 (CH3, C-20).

Isophytol **(7).** Colorless oil. ^{1}H-NMR ($CDCl_3$, 400 MHz) δ_H 5.90 (1H, dd, *J* = 10.7, 17.4 Hz, H-2), 5.15–5.20 (1H, dd, *J* = 1.3, 17.4 Hz, H-1a), 5.0–5.03 (1H, dd, *J* = 1.3, 10.7 Hz, H-1b), 1.25 (3H, br-s, H-20); The position of the other saturated methylene protons (total 16H); 0.85 (6H, s, H-16,17), 0.83 (3H, s, H-19), 0.83 (3H, s, H-18). ^{13}C-NMR ($CDCl_3$, 100 MHz) δ_C 145.3 (C-2), 111.5 (C-1), 73.3 (C-3), 42.7 (C-4), 39.4 (C-14), 37.4 (C-12), 37.4 (C-10), 37.4 (C-8), 37.3 (C-6), 32.8 (C-7), 29.7 (C-15), 27.9 (C-20), 24.8 (C-13), 24.5 (C-9), 22.7 (C-16), 22.6 (C-17), 21.3 (C-5), 19.7 (C-18), 19.6 (C-19).

(1, 2) Bis-nor-phytone **(8)**. Colorless oil. ^{1}H NMR ($CDCl_3$, 400 MHz): δ_H 2.38 (2H, br t, *J*=7.68 Hz, H-2), 2.11 (3H, s, H-18), 1.50 (1H, m, H-13), 0.85 (3H, *J*=6.6 Hz), 0.84 (3H, *J*=6.5 Hz), 0.83 (3H, *J*=6.6 Hz), 0.80 (3H, *J*=6.6 Hz). ^{13}C NMR ($CDCl_3$, 100 MHz): δ_C 209.5 (C-1), 44.2 (C-2), 21.4 (C-3), 36.5 (C-4), 32.8 (C-5), 37.3 (C-6), 24.4 (C-7), 37.4 (C-8), 32.7 (C-9), 37.2 (C-10), 24.8 (C-11), 39.4 (C-12), 27.8 (C-13), 22.6 (C-14), 22.7 (C-15), 19.6 (C-16), 19.8 (C-17), 29.9 (C-18).

Pheophorbide-a methyl ester **(9).** Black-green powder. ^{1}H NMR ($CDCl_3$, 400 MHz) δ_H 9.29 (1H, s, H-5), 9.47 (1H, s, H-10), 8.61 (1H, s, H-20), 7.90 (1H, dd, *J*=11.5; 17,8 Hz, H-3^1), 6.28 (1H, s, H-13^2), 6.25 (1H, dd, *J*=17.6 Hz, H-3^{2a}), 6.15 (1H, dd, *J*=11.6 Hz, H-3^{2b}), 4.49 (1H, q, *J*=7.5 Hz, H-18), 4.23 (1H, br d, *J*=8.5 Hz H-17), 3.89 (3H, s, C-13^3 ,-OMe), 3.59 (3H, s, C-17^3 ,-OMe), 3.66 (3H, s, H3-12^1), 3.59 (2H, q, *J*=7.5 Hz, H-8^1), 3.36 (3H, s, H3-2^1), 3.12 (3H, s, H3-7^1), 2.66 (1H, m, H-17^{1a}), 2.55 (1H, m, H-17^{2a}), 2.32 (1H, m, H-17^{1b}), 2.26 (1H, m, H-17^{2b}), 1.84 (3H, d, *J*=7.3 Hz, H3-18^1), 1.63 (3H, t, *J*=7.5 Hz, H3-8^2). ^{13}C NMR ($CDCl_3$, 100 MHz): δ_C 189.6 (C13^1), 173.4 (C-17^3), 172.4 (C-19), 169.6 (C-13^3), 161.7 (C-16), 154.4 (C-6), 151.0 (s, C-9), 149.9 (C14), 145.0 (C-8), 142.2 (C-1), 137.9 (C-11), 136.3 (C-4), 135.9 (C-7), 136.6 (C-3), 132.1 (C-2), 129.0 (C-12), 128.9 (C-3^1), 128.9 (C-13), 122.9 (CH_2, C-3^2), 105.3 (C-15), 104.4 (CH, C-10), 97.5 (CH, C-5), 93.5 (CH, C-20), 64.8 (d, C-13^2), 52.9 (CH_3, C-13^3 ,-OMe), 51.8 (CH_3, C-17^3 ,-OMe), 51.2 (CH, C-17), 50.2 (CH, C-18), 31.3 (CH_2, C17^2), 29.8 (CH_2, C-17^1), 23.2 (CH_3, C-18^1), 19.4 (CH_2, C-8^1), 17.4 (CH_3, C-8^2), 12.1 (CH_3, C-2^1), 12.2 (CH_3, C-12^1), 11.2 (CH_3, C-7^1).

Pheophorbide-b methyl ester **(10).** Brown powder. ^{1}H NMR ($CDCl_3$) 10.92 (1H, s, H-7^{1}), 10.13 (1H, s, H-5), 9.38 (1H, s, H-10), 8.53 (1H, s, H-20), 7.89 (1H, dd, *J*=11.6; 17.8 Hz, H-3^{1}), 6.21 (1H, s, H-13^{2}), 6.33 (1H, d, *J*=17.8 Hz, H-3^{2a}), 6.19 (1H, d, *J*=11.7 Hz, H-3^{2b}), 4.44 (1H, m, H-18), 4.17 (1H, m, H-17), 3.9 (3H, s, C-13^{2} ,-OMe), 3.59 (3H, s, C-17^{3} ,-OMe), 3.50 (3H, s, H3-12^{1}), 3.78 (2H, q, *J*=7.2 Hz, H-8^{1}), 3.33 (3H, s, H3-2^{1}), 2.56 (1H, m, H-17^{1a}), 2.66 (1H, m, H-17^{2a}), 2.28 (1H, m, H-17^{1b}), 2.33 (1H, m, H-17^{2b}), 1.83 (3H, d, *J*=7.3 Hz, H3-18^{1}), 1.68 (3H, t, *J*=7.5 Hz, H3-8^{2}). ^{13}C NMR ($CDCl_3$): δ_C 189.6 (C13^{1}), 173.4 (C-17^{3}), 174.2 (C-19), 169.4 (C-13^{3}), 164.4 (C-16), 150.7 (C-6), 146.5 (s, C-9), 150.9 (C14), 159.0 (C-8), 143.8 (C-1), 138.0 (C-11), 137.9 (C-4), 132.5 (C-7), 137.3 (C-3), 132.5 (C-2), 132.8 (C-12), 128.7 (C-3^{1}), 129.8 (C-13), 123.8 (CH_2, C-3^{2}), 105.2 (C-15), 106.9 (CH, C-10), 101.5 (CH, C-5), 93.7 (CH, C-20), 64.7 (d, C-13^{2}), 53.2 (CH_3, C-13^{2} ,-OMe), 51.9 (CH_3, C-17^{3} ,-OMe), 51.5 (CH, C-17), 50.3 (CH, C-18), 29.2 (CH_2, C17^{2}), 31.3 (CH_2, C-17^{1}), 23.3 (CH_3, C-18^{1}), 19.5 (CH_2, C-8^{1}), 19.5 (CH_3, C-8^{2}), 12.3 (CH_3, C-2^{1}), 12.4 (CH_3, C-12^{1}), 187.6 (C, C-7^{1}).

Stigma-5-en-3-O-β-glucoside **(11).** White, amorphous powder. ^{1}H-NMR (DMSO-d_6): 0.64 (s, 3H-18), 0.95 (s, 3H-19), 0.89 (d, *J*=6.5 Hz, 3H-21), 0.79 (d, *J*=7.1, 3H-26), 0.80 (d, *J*=6.8, 3H-27), 0.81 (d, *J*=6.8, 3H-29), 3.44 (m, H-3), 5.31 (d, J=4.8 Hz, H-6), 1.37 (d, *J*=4.5 Hz, H-8), 0.89 (d, *J*=6.5 Hz, H-9), 0.95 (s, H-14), 1.07 (d, *J* =9.9Hz, H-17), 1.34 (m, H-20), 0.90 (d, *J* =6.5 Hz H-24), 1.63 (m, H-25), 4.22 (d, *J* =7.8 Hz H-1'), 2.89 (dt, *J* =4.6; 8.2 H-2'), 3.12 (m, H-3'), 3.01 (m, H-4'), 3.06 (dd, *J* =2.0; 5.9 Hz, H-5'). ^{13}C-NMR (DMSO-d_6): δ_C 36.9 (C-1), 29.3 (C-2), 76.8 (C-3), 38.4 (C-4), 140.5 (C-5), 121.4 (C-6), 31.4 (C-7), 31.5 (C-8), 49.6 (C-9), 36.3 (C-10), 20.6 (C-11), 39.17 overlaping with solvent (C-12), 40.8 (C-13), 56.2 (C-14), 23.9 (C-15), 27.9 (C-16), 55.4 (C-17), 11.7 (C-18), 19.0 (C-19), 35.5 (C-20), 18.7 (C-21), 33.4 (C-22), 25.45 (C-23), 45.2 (C-24), 28.7 (C-25), 18.55 (C-26), 19.83 (C-27), 28.7 (C-27), 22.6 (C-28), 11.7 (C-29), 108 (C-1'), 73.5 (C-2'), 77.0 (C-3'), 70.2 (C-4'), 76.8 (C-5').

Vanillic acid **(12).** Colorless needles. ^{1}H-NMR (CD_3OD): δ 6.84 (d, *J*= 8.8 Hz, H-5), 7.56 (d, *J*=8.8 Hz, H-2), 7.55 (dd, *J*=1.9; 8.8 Hz, H-6), 3.88 (s, OCH_3). ^{13}C-NMR (CD_3OD): δ_C 123.4 (C-1), 113.7 (C-2), 148.7 (C-3), 152.7 (C-4), 115.8 (C-5), 125.3 (C-6), 170.0 (C-7), 56.4 (-OMe).

Abscisic acid **(13).** White oil. ^{1}H NMR (CD_3CN, 400 HMz): δ_H 5.71 (1H, s, H-2), 7.78 (1H, d, *J*=16.0 Hz, H-4), 6.24 (1H, d, *J*= 16.2 Hz, H-5), 5.82 (1H, s, H-3'), 3.26 (2H, brs, H-5'), 1.99 (3H, s, H-6), 1.83 (3H, s, H-7'), 1.01 (3H, s, H-8'), 0.95 (3H, s, H-9'). ^{13}C NMR (CD_3CN, 100 MHz). δ_C 170.6 (C-1), 119.2 (C-2), 164.2 (C-3), 129.2 (C-4), 138.6 (C-5), 21.9 (C-6), 80.8 (C-1'), 151.5 (C-2'), 128.1 (C-3'), 198.9 (C-4'), 50.9 (C-5'), 42.8 (C-6'), 19.8 (C-7'), 24.1 (C-8'), 25.2 (C-9').

Gallic acid **(14).** White, needles. ^{1}H-NMR (CD_3OD): δ_H 7.07 (s, 2 H). ^{13}C-NMR (CD_3OD): δ_C 110.3 (C-2 and C-6), 121.9 (C-1), 139.5 (C-4); 146.3 (C-3 and C-5), 169.8 (C-7).

Myricetin-3-O-rhamnoside **(15)**. Pale yellow powder. HR-ESI-MS *m/z* 463.0883 [M-H]$^-$ (calculated for $C_{21}H_{19}O_{12}$, 463.0877, Δ -1.29 ppm), ^{1}H NMR (DMSO-d_6): δ_H 0.83 (3H, d, *J*=6.2 Hz), 5.19 (1H, d, *J*=1.2 Hz), 6.19 (1H, d, *J*=1.9 Hz), 6.36 (1H, d, *J*=1.9 Hz), 6.88 (2H, s), 3.97 (1H, dd, *J*=1.6; 3.0 Hz), 3.56 (1H, dd, *J*=3.3; 9.4 Hz), 3.15 (1H, t, *J*= 9.4), 3.36 (1H, m). ^{13}C NMR (DMSO-d_6): δ_C 157.5 (C-2), 134.3 (C-3), 177.8 (C-4), 161.3 (C-5), 98.7 (C-6), 164.4 (C-7), 93.6 (C-8), 156.4 (C-9), 104.0 (C-10), 119.6 (C-1'), 107.9 (C-2'), 145.8 (C-3'), 136.4 (C-4'), 145.8 (C-5'), 107.9 (C-6'); 3-*O*-rhamnose: 101.9 (C-1''), 70.0 (C-2''), 70.3 (C-3''), 71.2 (C-4''), 70.6 (C-5''), 17.6 (C-6'').

Quercetin-3-O-rhamnoside **(16).** Pale yellow powder. HR-ESI-MS *m/z* 447.0930 [M-H]$^-$ (calculated for $C_{21}H_{19}O_{11}$, 447.0927, Δ -0.67 ppm), ^{1}H NMR (DMSO-d$_6$) δ: 0.82 (3H, d, *J*=6.0 Hz), 5.22 (1H, d, *J*=1. 3 Hz), 6.19 (1H, d, *J*=2.0 Hz), 6.38 (1H, d, *J*=2.0 Hz), 6.87 (1H, d, *J*=8.3 Hz), 7.26 (1H, dd, *J*=2.1; 8.3 Hz), 7.30 (1H, d, *J*=2.1 Hz). ^{13}C NMR (DMSO-d$_6$): δ_C 157.1 (C-2), 134.1 (C-3), 177.7 (C-4), 161.3 (C-5), 98.8 (C-6), 164.8 (C-7), 93.7 (C-8), 156.5 (C-9), 103.8 (C-10), 120.6 (C-1'), 115.4 (C-2'), 145.3 (C-3'), 148.6 (C-4'), 115.6 (C-5'), 121.05 (C-6'); 3-*O*-rhamnose: 101.8 (C-1''), 70.3 (C-2''), 70.5 (C-3''), 71.2 (C-4''), 70.0 (C-5''), 11.5 (C-6'').

Myricetin-3',5'dimethylether-3-O-Galactopyranoside **(17).** Yellow powder. ^{1}H NMR (DMSO-d_6) δ_H 6.16 (1H, s,), 6.34 (1H, s), 7.19 (1H, s), 3.45 (1H, m), 3.28 (1H, m). ^{13}C NMR (DMSO-d_6): δ_C 156.0 (C-2), 133.8 (C-3), 177.3 (C-4), 161.1 (C-5), 98.4 (C-6), 164.4 (C-7), 94.1 (C-8), 156.0 (C-9), 103.6 (C-10), 119.7 (C-1'), 108.5 (C-2'), 145.3 (C-3'), 136.6 (C-4'), 145.3 (C-5'), 108.5 (C-6'); 3-O-glucose: 101.7 (C-1''), 73.1 (C-2''), 71.1 (C-3''), 68.7 (C-4''), 75.7 (C-5''), 59.8 (C-6'').

Quercetin-3-O-galactopyranoside **(18).** Yellow powder. HR-ESI-MS *m/z* 463.0882 [M-H]$^-$ (calculated for $C_{21}H_{19}O_{12}$, 463.0877, Δ 1.07 ppm), ^{1}H NMR (DMSO-d_6) δ_H 6.16 (1H, s,), 6.37 (1H, s), 6.79 (1H, d, *J*=2.0 Hz), 7.51 (1H, d, *J*=2.1 Hz), 7.65 (1H, dd, *J*=2.1, 8.5 Hz), 3.44 (1H, m), 3.29 (1H, m). ^{13}C NMR (DMSO-d_6): δ_C 156.5 (C-2), 133.8 (C-3), 165.6 (C-4), 156.7 (C-5), 99.3 (C-6), 161.1 (C-7), 94.1 (C-8), 156.8 (C-9), 104.1 (C-10), 121.5 (C-1'), 122.4 (C-2'), 116.0 (C-3'), 145.3 (C-4'), 149.0 (C-5'), 115.6 (C-6'); 3-*O*-glucose: 102.3 (C-1''), 71.6 (C-2''), 73.5 (C-3''), 68.4 (C-4''), 76.2 (C-5''), 60.6 (C-6'').

Epicatechin-3-galloylester **(19)**. Yellow powder. HR-ESI-MS *m/z* 441.0824 [M-H]$^-$ (calculated for $C_{22}H_{17}O_{10}$, 441.0822, Δ 0.45 ppm) ^{1}H NMR (DMSO-d_6) δ_H 6.85 (1H, s,), 6.81

(1H, s), 6.73 (1H, s), 5.93 (1H, s), 5.83 (1H, s), 5.33 (1H, s), 5.24 (1H, s), 5.06 (1H, s), 5.02 (1H, s), 5.24 (1H, s). ^{13}C NMR (DMSO-d_6): δ_C 77.2 (C-2), 68.2 (C-3), 25.6 (C-4), 156.4 (C-5), 94.4 (C-6), 155.5 (C-7), 95.4 (C-8), 156.4 (C-9), 97.3 (C-10), 129.3 (C-1'), 117.7 (C-2'), 115.0 (C-3'), 144.9 (C-4'), 144.9 (C-5'), 114.2 (C-6'); 3-*O*-galloyl ester: 165.3 (C-1''), 108.7 (C-2''), 145.5 (C-3''), 138.5 (C-4''), 145.5 (C-5''), 108.7 (C-6'').

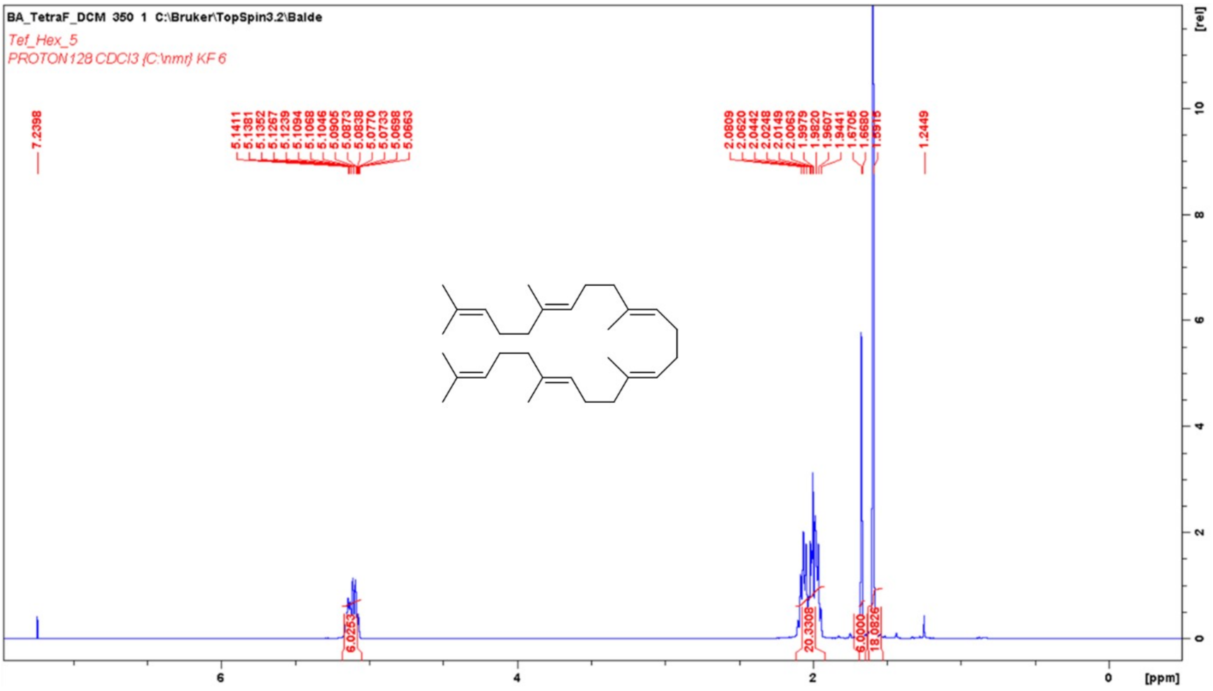

Figure S.1. ^{1}H NMR spectrum ($CDCl_3$, 400 MHz) of squalene **(1).**

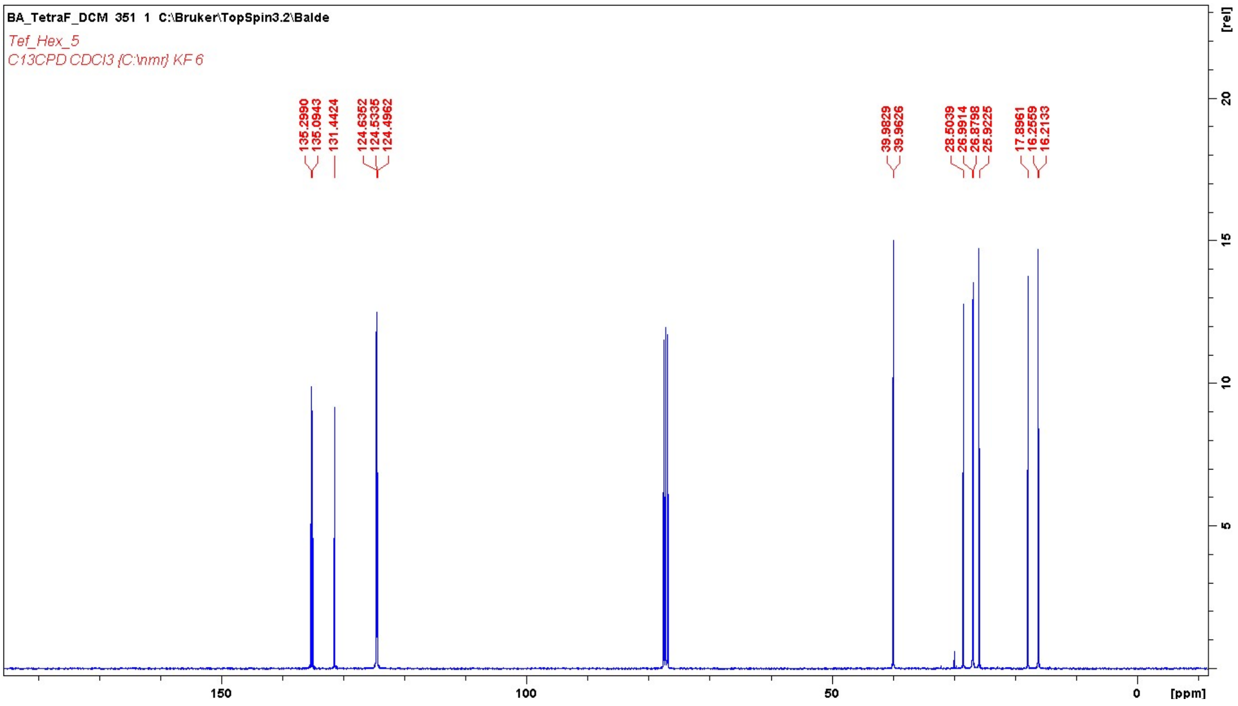

Figure S.2. ^{13}C NMR spectrum ($CDCl_3$, 100 MHz) of squalene **(1).**

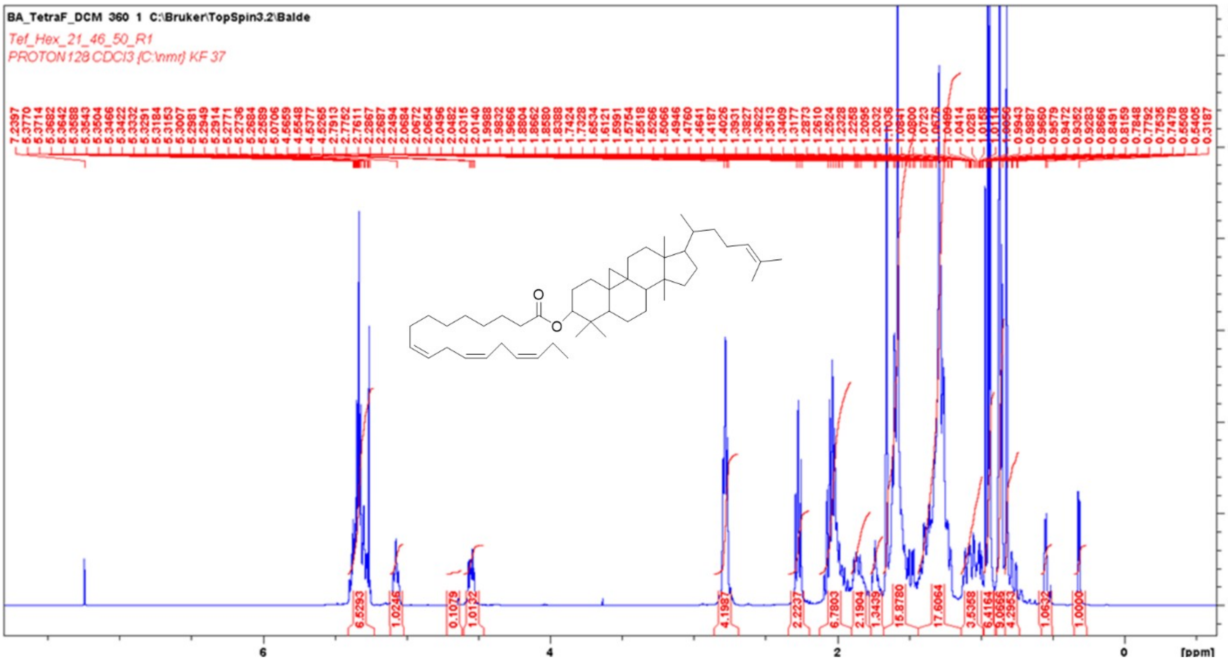

Figure S.3. ^{1}H NMR spectrum ($CDCl_3$, 400 MHz) of cycloart-24-en-3β-yl α-linolenate **(2)**

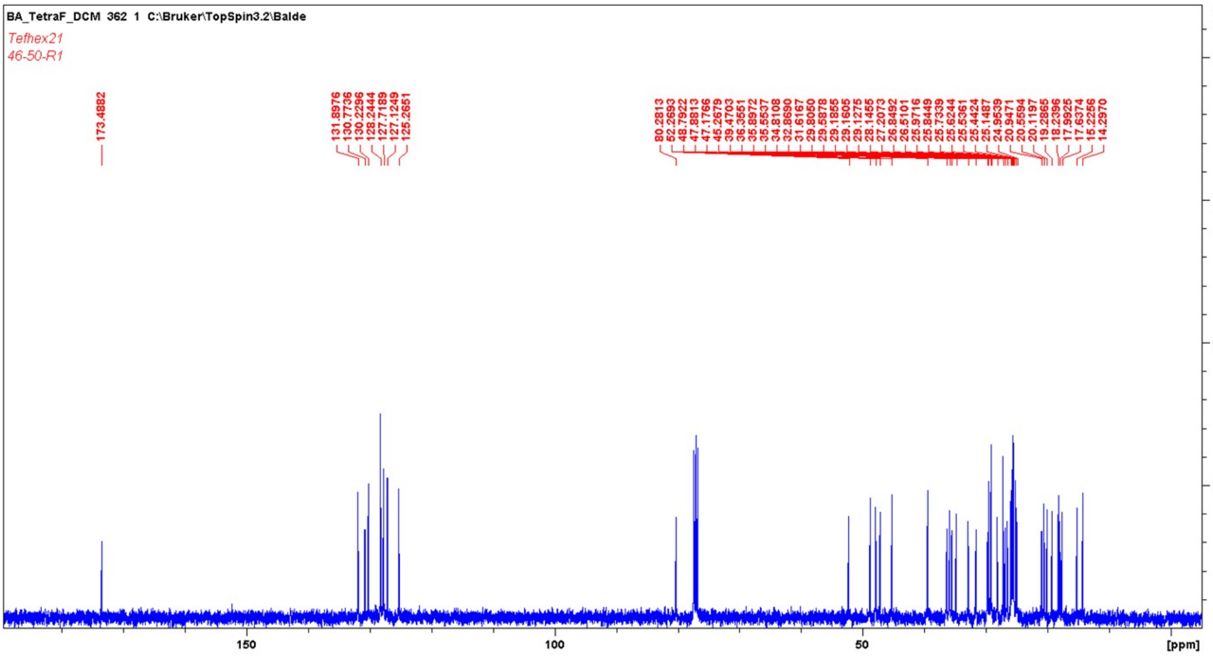

Figure S.4. ^{13}C NMR spectrum ($CDCl_3$, 100 MHz) of cycloart-24-en-3β-yl α-linolenate (2)

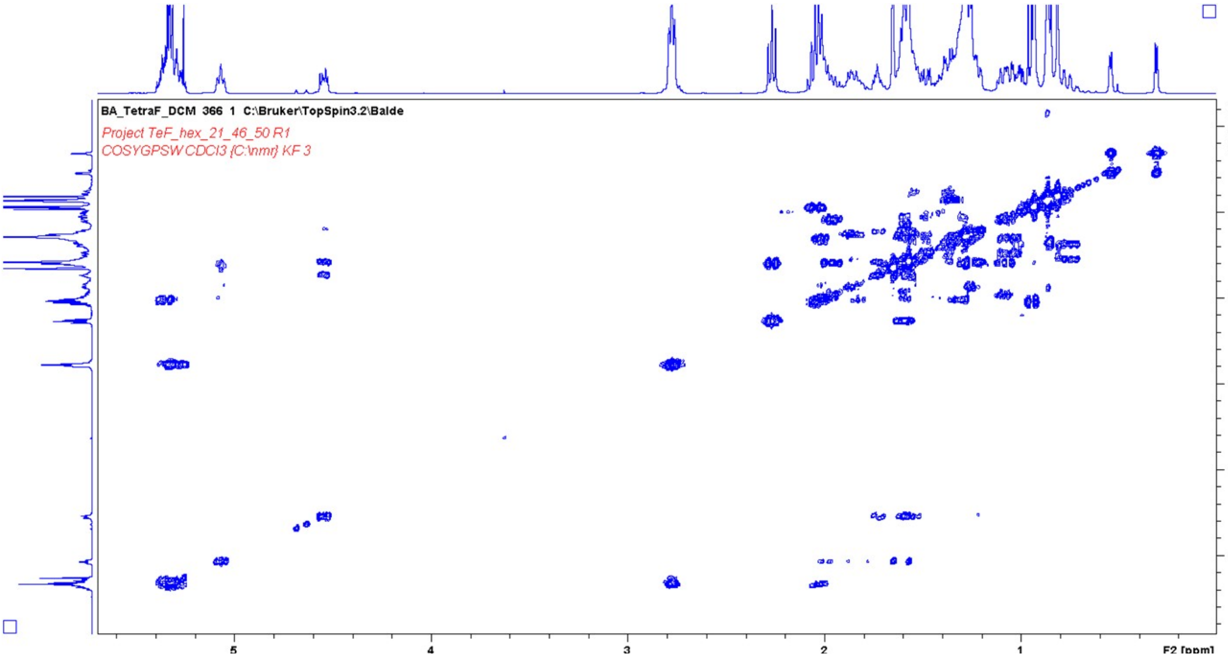

Figure S.5. COSY spectrum ($CDCl_3$) of Cycloart-24-en-3β-yl α-linolenate **(2)**

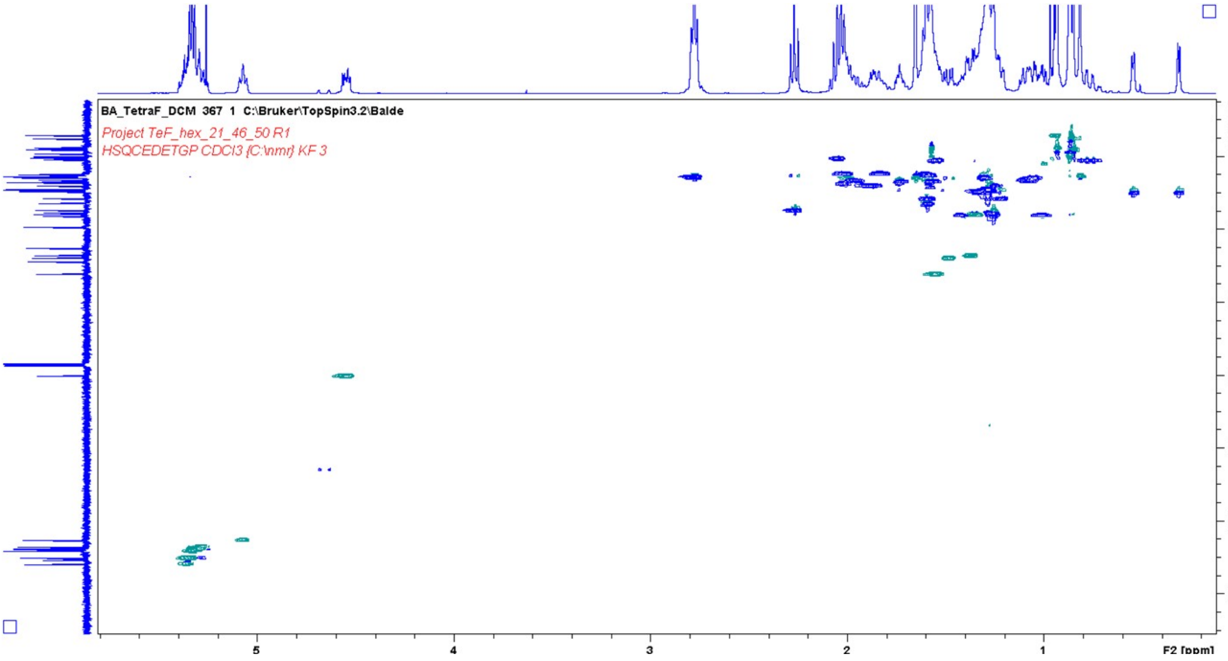

Figure S.6. COSY spectrum ($CDCl_3$) of Cycloart-24-en-3β-yl α-linolenate **(2)**

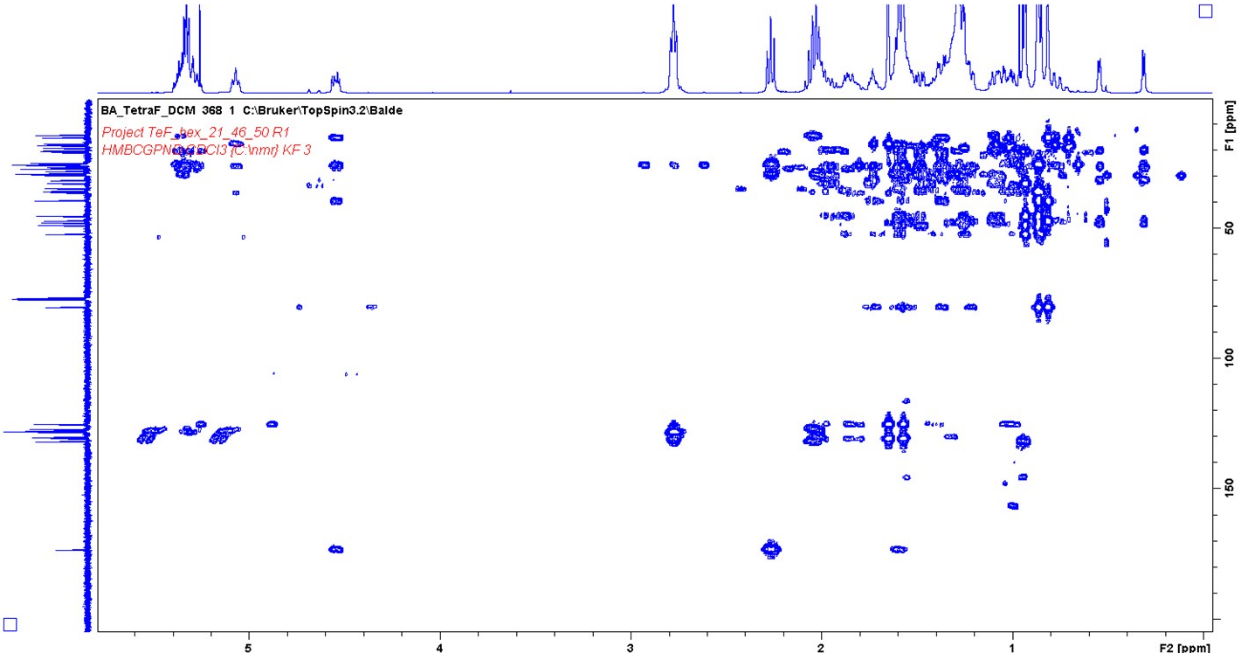

Figure S.7. COSY spectrum ($CDCl_3$) of Cycloart-24-en-3β-yl α-linolenate **(2)**

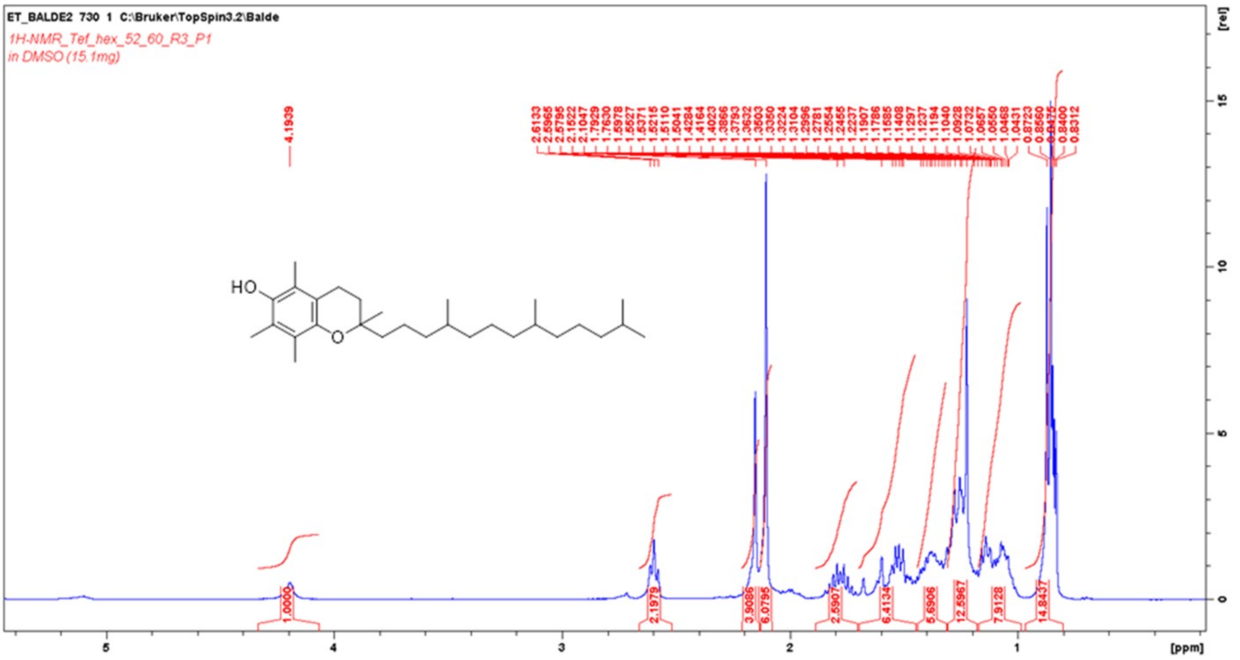

Figure S.8. ^{1}H NMR spectrum (DMSO-*d*6, 400 MHz) of α-Tocopherol **(3)**

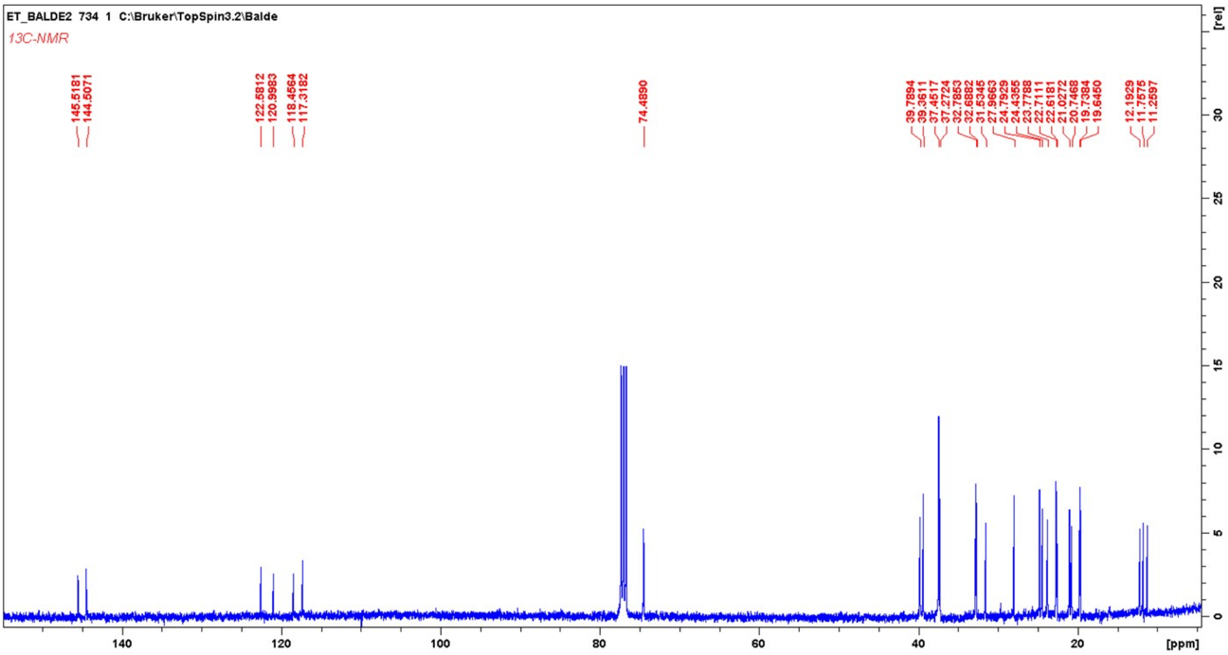

Figure S.9. ^{13}C NMR spectrum (DMSO-*d6*, 100 MHz) of α-Tocopherol **(3)**

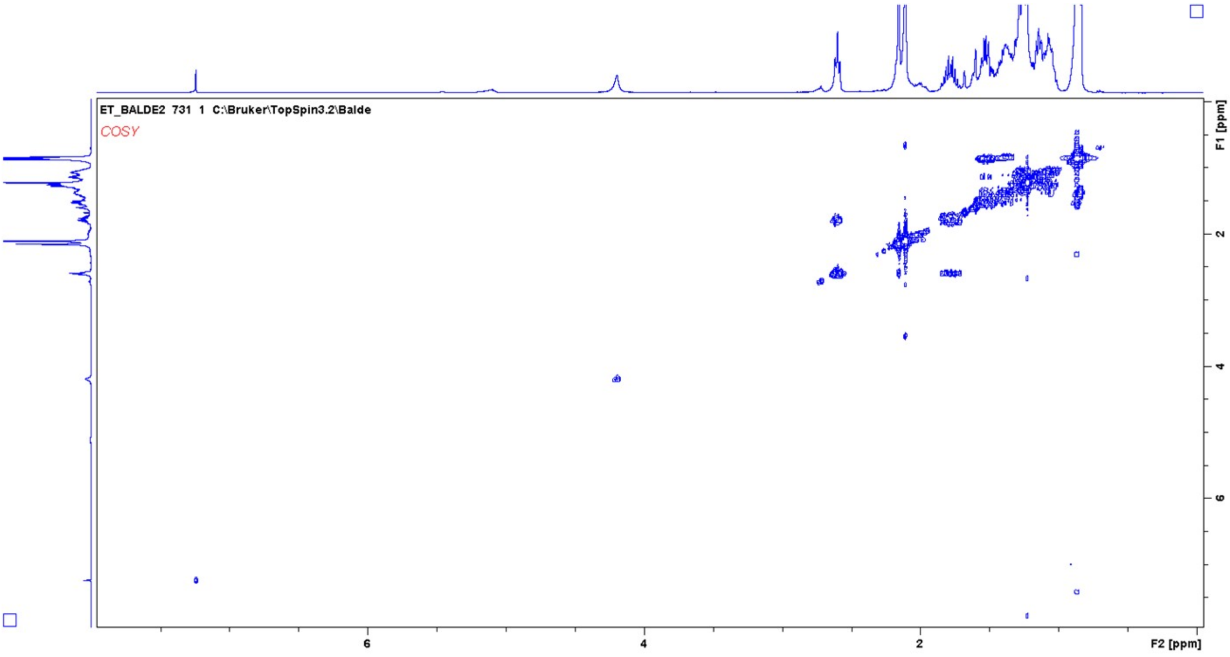

Figure S.10. COSY spectrum (DMSO-*d6*) of α-Tocopherol **(3)**

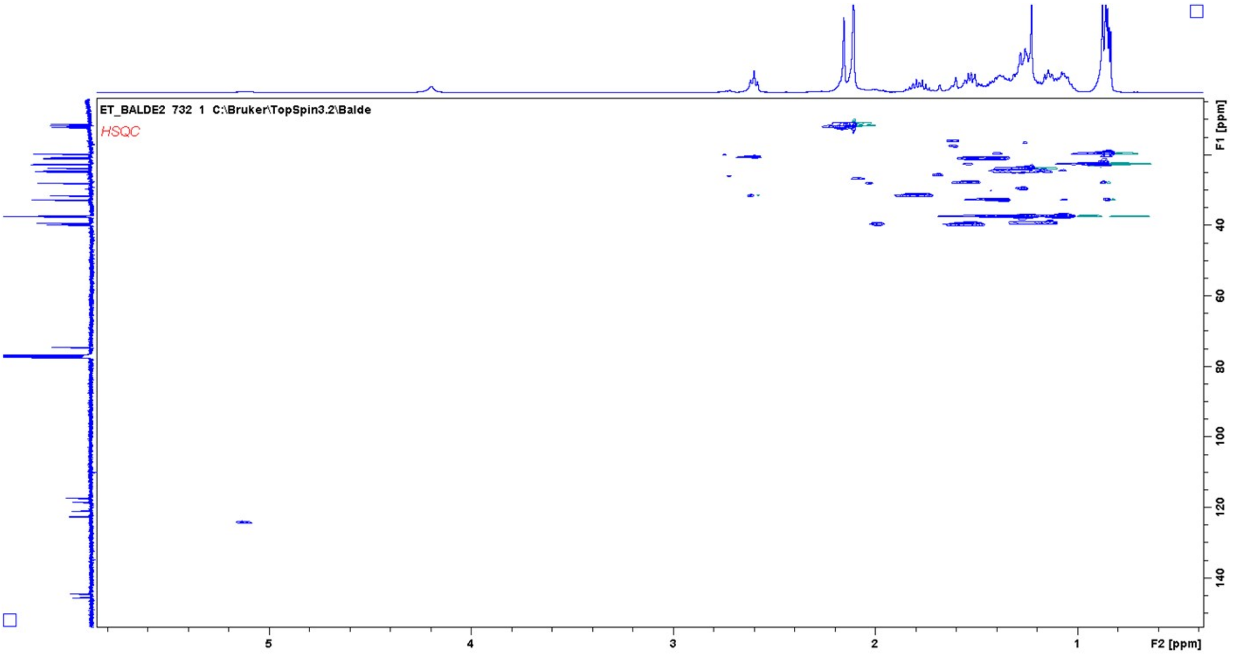

Figure S.11. HSQC spectrum (DMSO-*d6*) of α-Tocopherol **(3).**

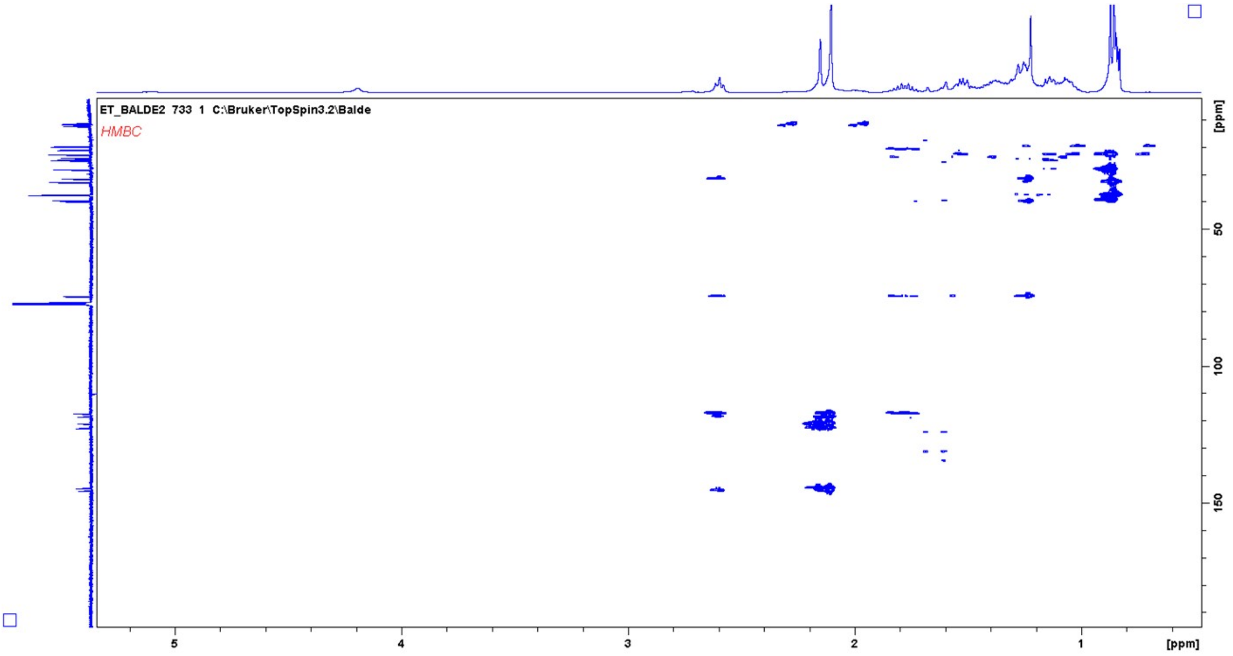

Figure S.12. HMBC spectrum (DMSO-*d6*) of α-Tocopherol **(3).**

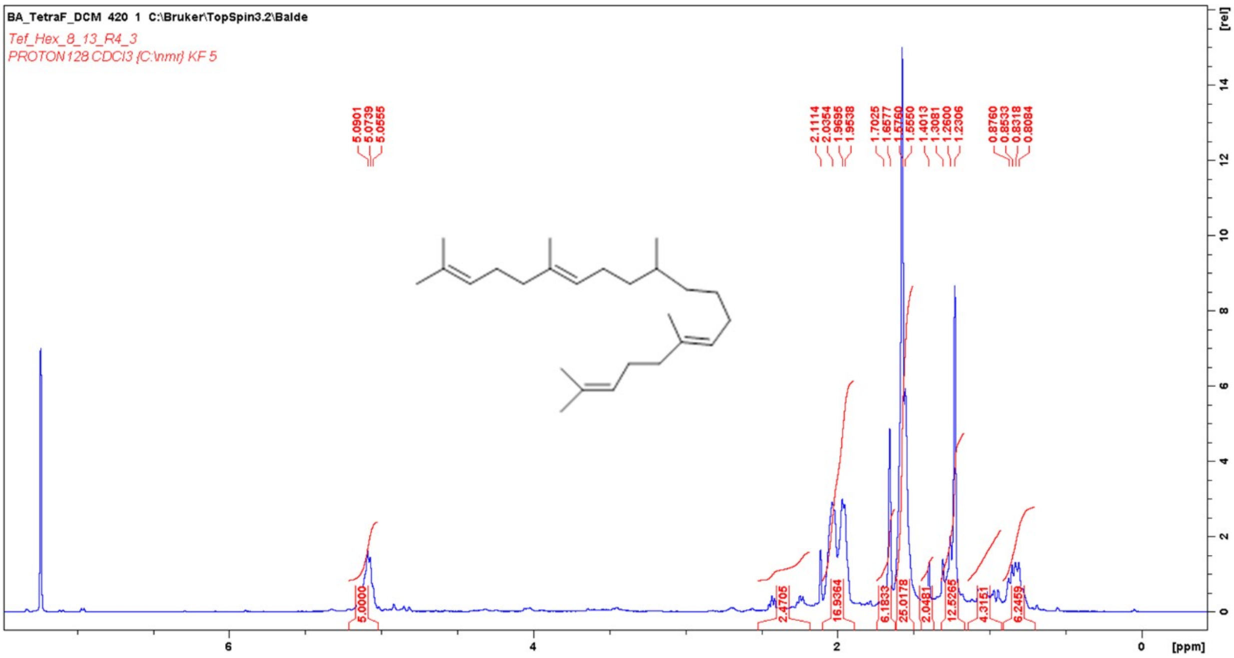

Figure S.13. ^{1}H NMR spectrum ($CDCl_3$, 400 MHz) of Trans-pentamethyl-Icosa-tetraene **(4).**

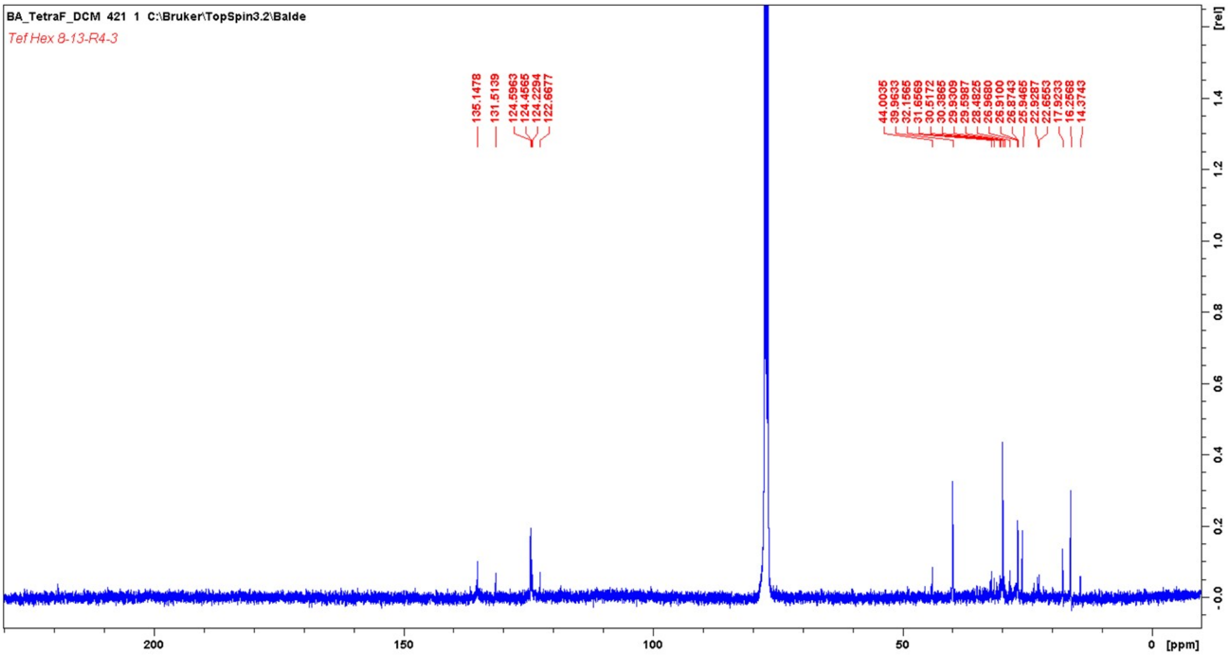

Figure S.14. ^{13}C NMR spectrum ($CDCl_3$, 100 MHz) of Trans-pentamethyl-Icosa-tetraene **(4).**

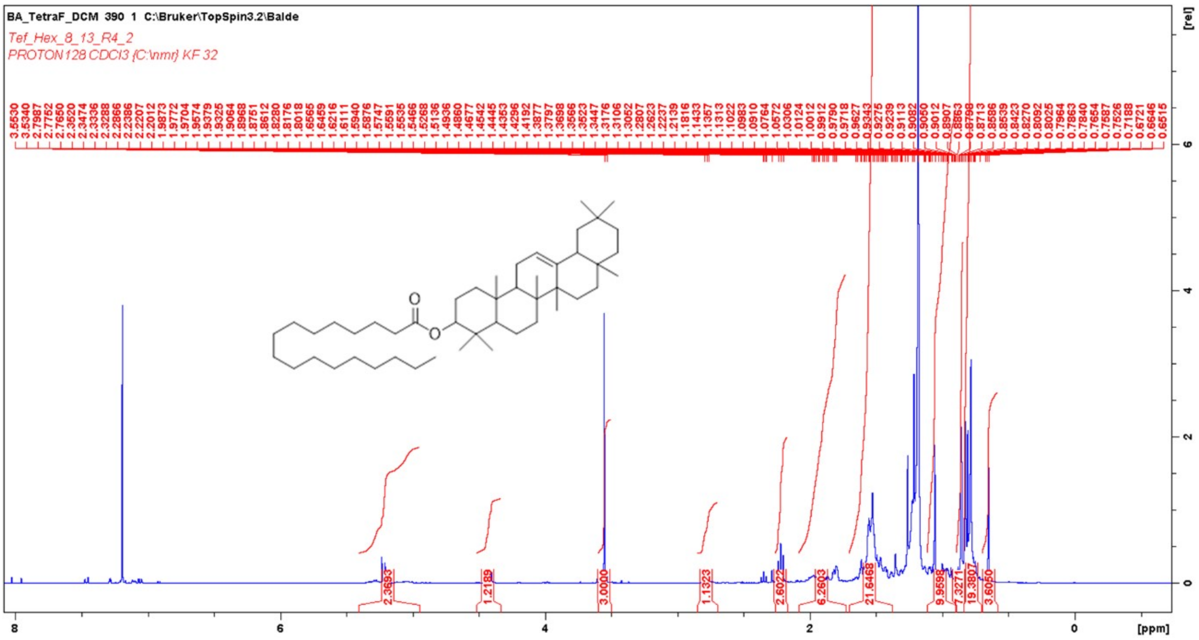

Figure S.15. ^{1}H NMR spectrum ($CDCl_3$, 400 MHz) of 3-β-hydroxy-olean-12-ene-heptadecanoate **(5).**

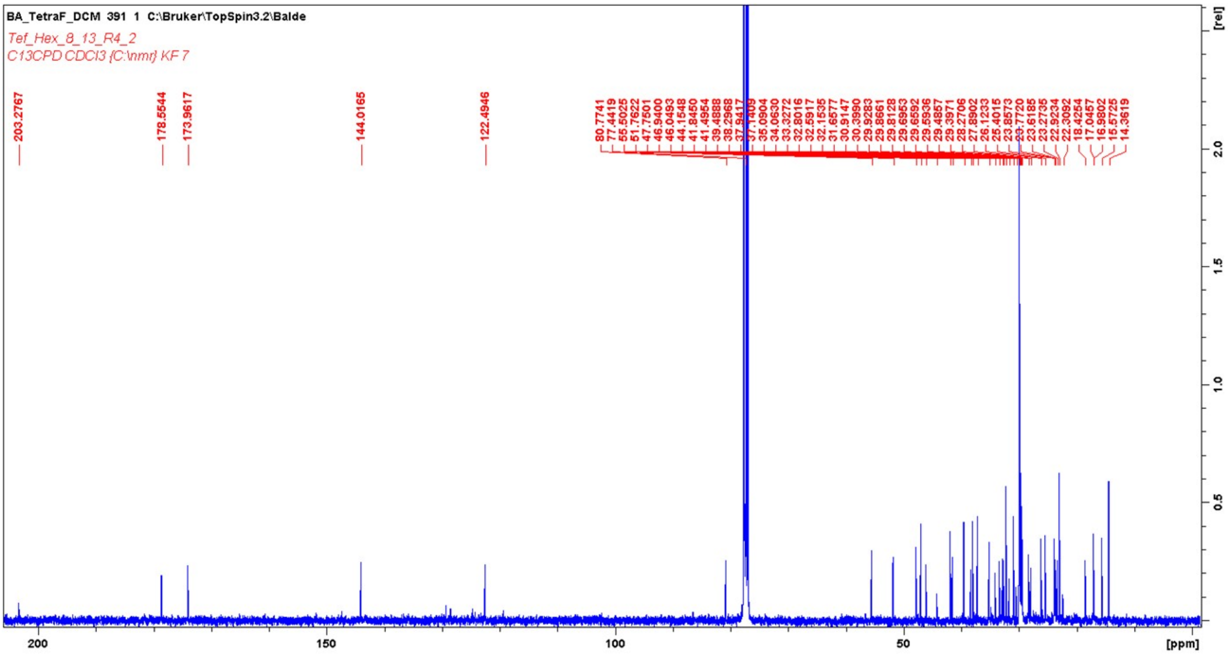

Figure S.16. ^{13}C NMR spectrum ($CDCl_3$, 100 MHz) of 3-β-hydroxy-olean-12-ene-heptadecanoate **(5).**

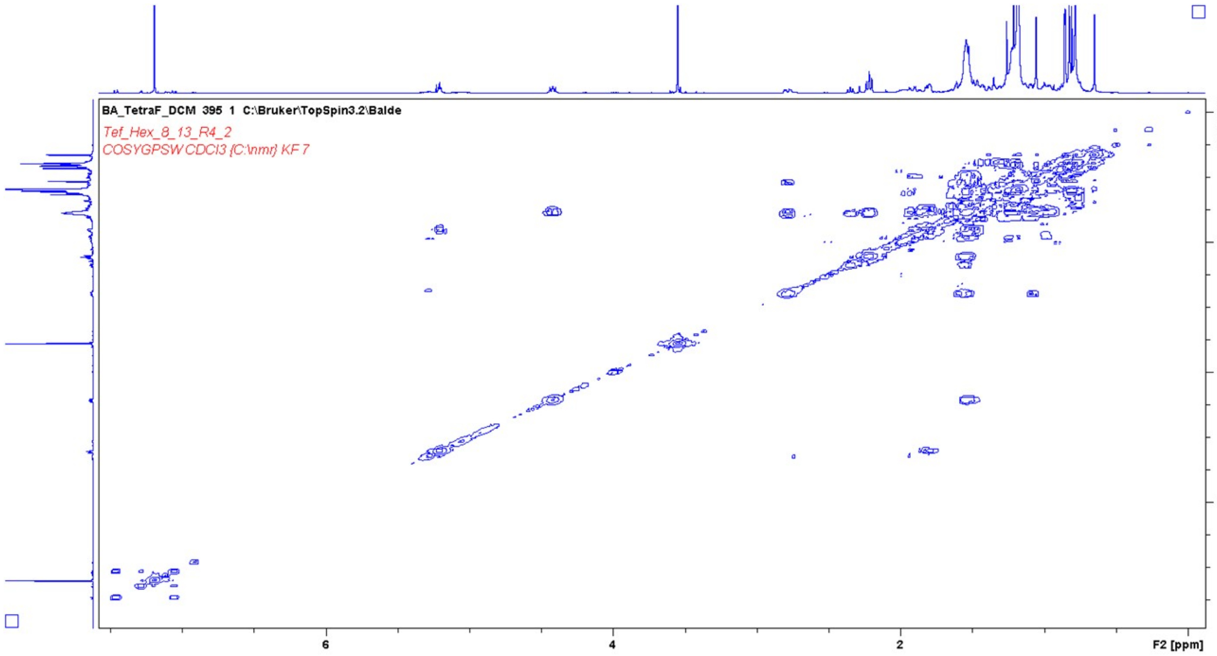

Figure S.17. COSY spectrum ($CDCl_3$) of 3-β-hydroxy-olean-12-ene-heptadecanoate **(5).**

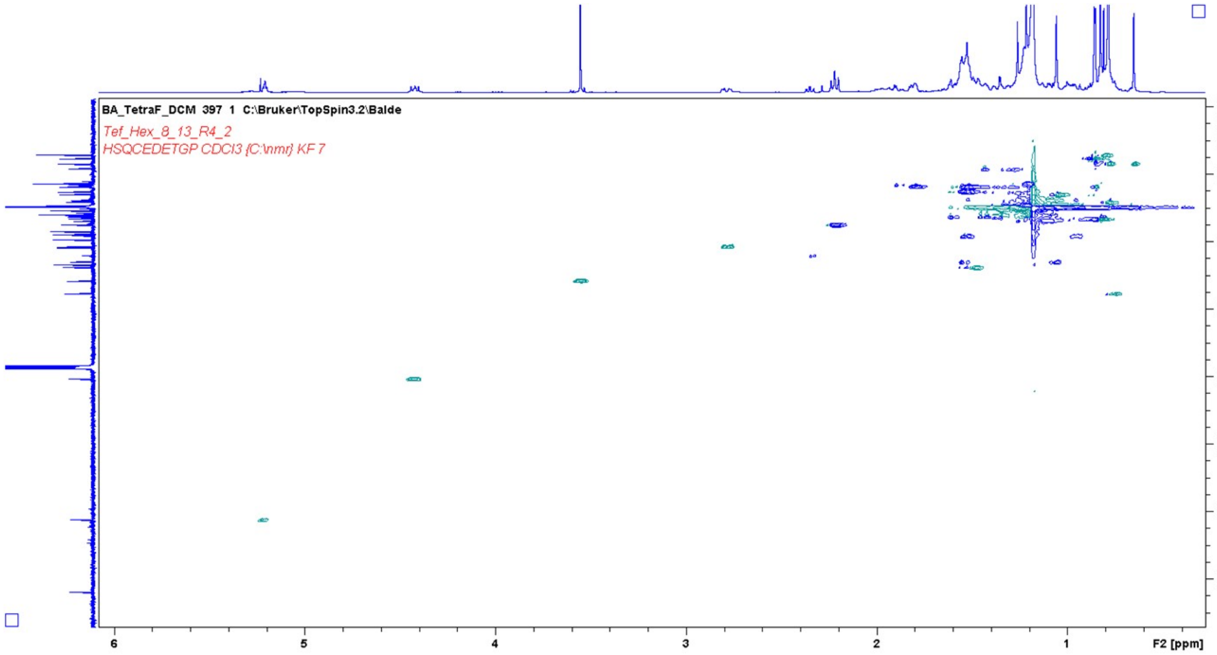

Figure S.18. HSQC spectrum ($CDCl_3$) of 3-β-hydroxy-olean-12-ene-heptadecanoate **(5).**

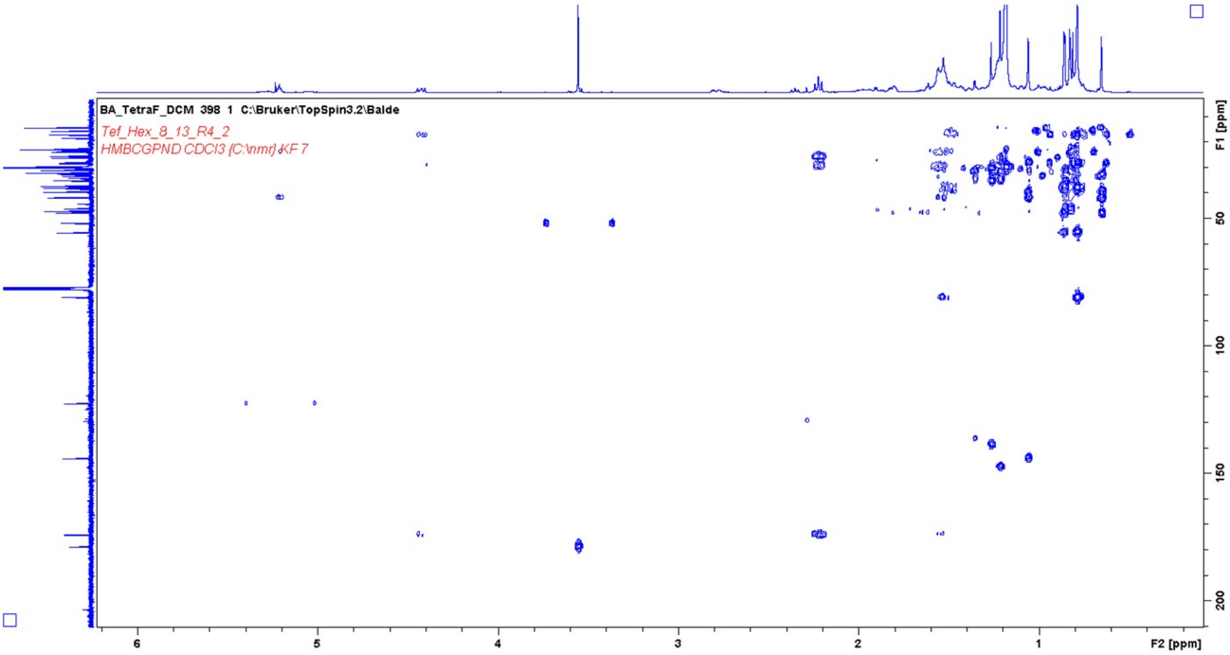

Figure S.19. HMBC spectrum ($CDCl_3$) of 3-β-hydroxy-olean-12-ene-heptadecanoate **(5)**

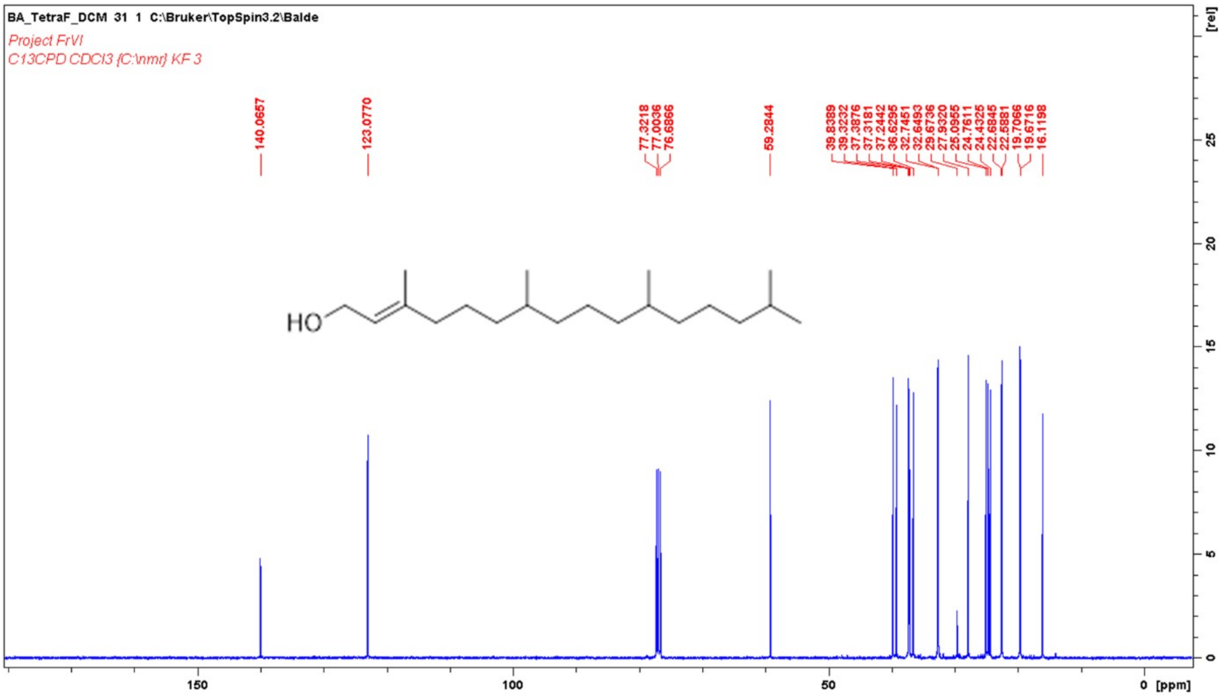

Figure S.20. ^{13}C NMR spectrum ($CDCl_3$, 100 MHz) of Phytol **(6)**

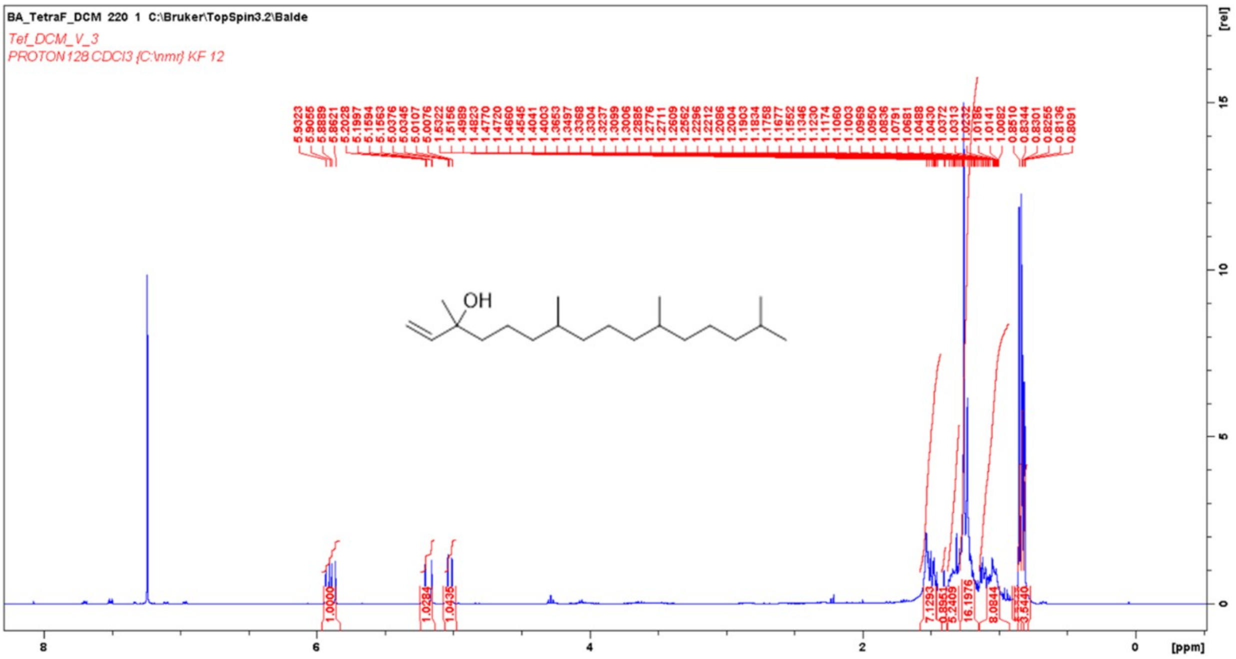

Figure S.21. ^{1}H NMR spectrum ($CDCl_3$, 400 MHz) of Isophytol **(7)**

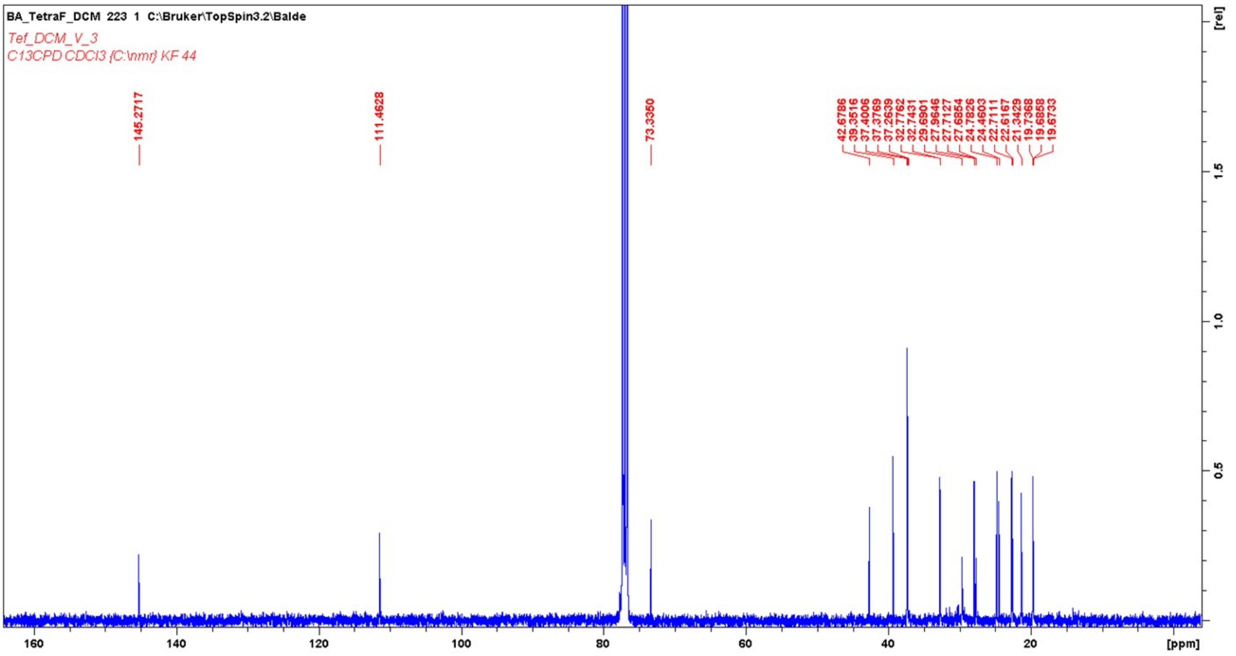

Figure S.22. ^{13}C NMR spectrum ($CDCl_3$, 100 MHz) of Isophytol **(7).**

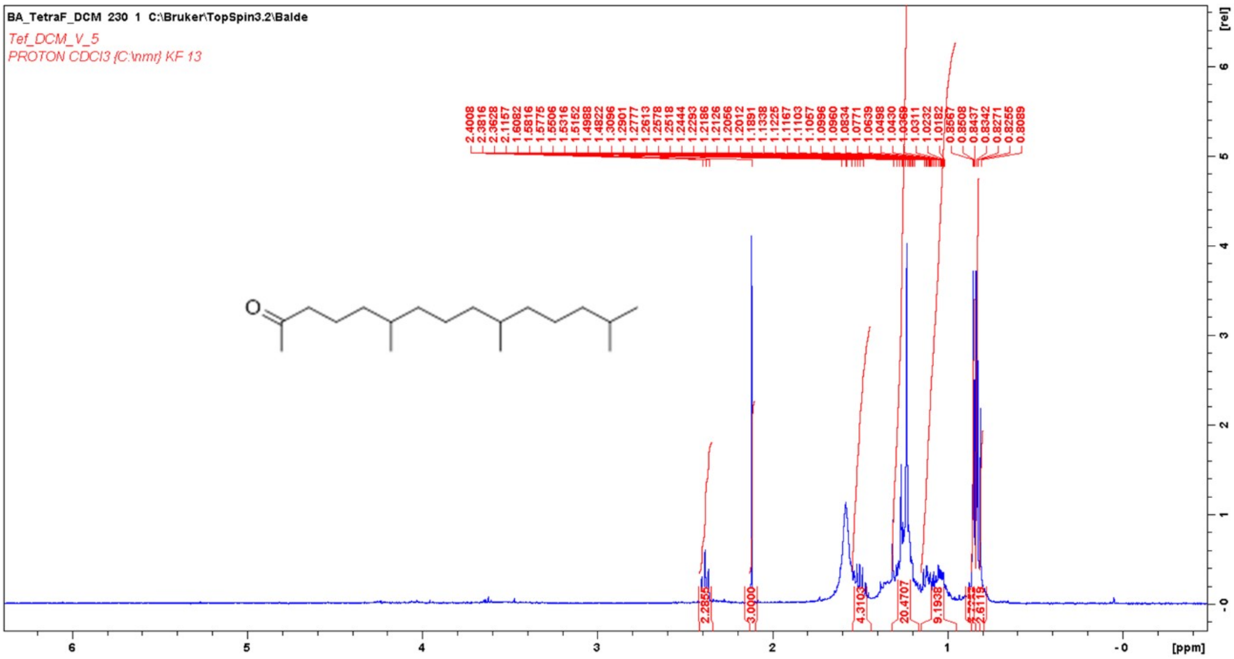

Figure S.23. ^{1}H NMR spectrum ($CDCl_3$, 400 MHz) of (1,2)-Bis-nor-phytone **(8)**

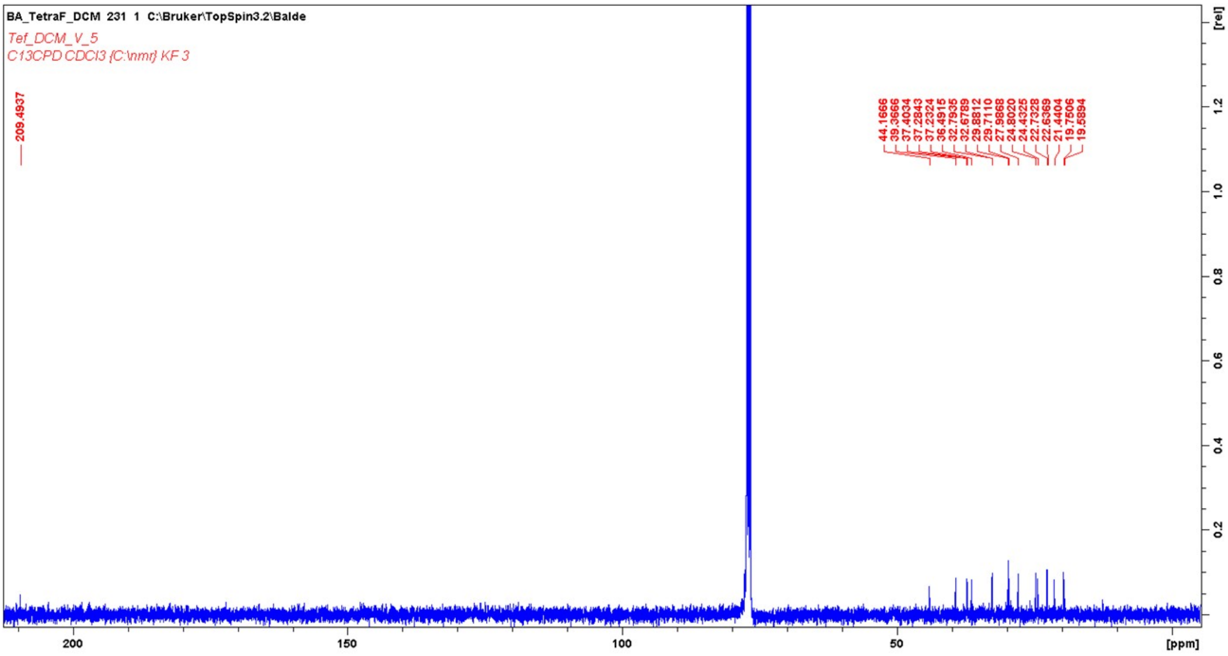

Figure S.24. ^{13}C NMR spectrum ($CDCl_3$, 100 MHz) of (1,2)-Bis-nor-phytone **(8)**

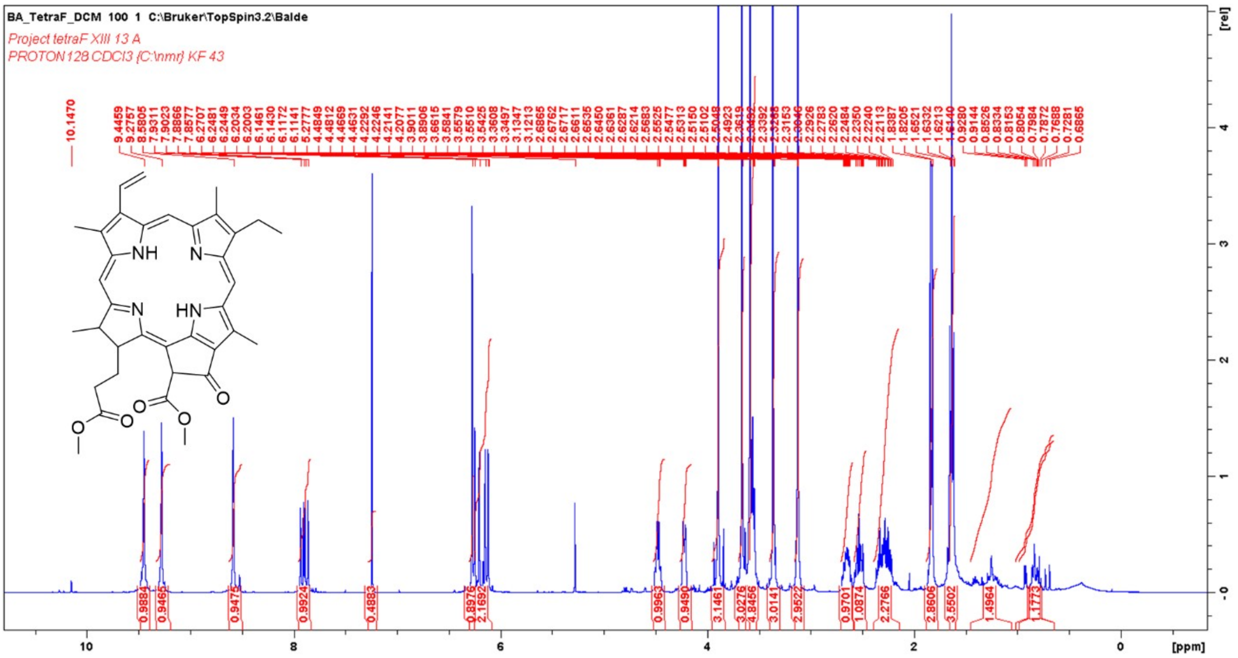

Figure S.25. ^{1}H NMR spectrum ($CDCl_3$, 400 MHz) of Pheophorbide-A methyl ester **(9)**

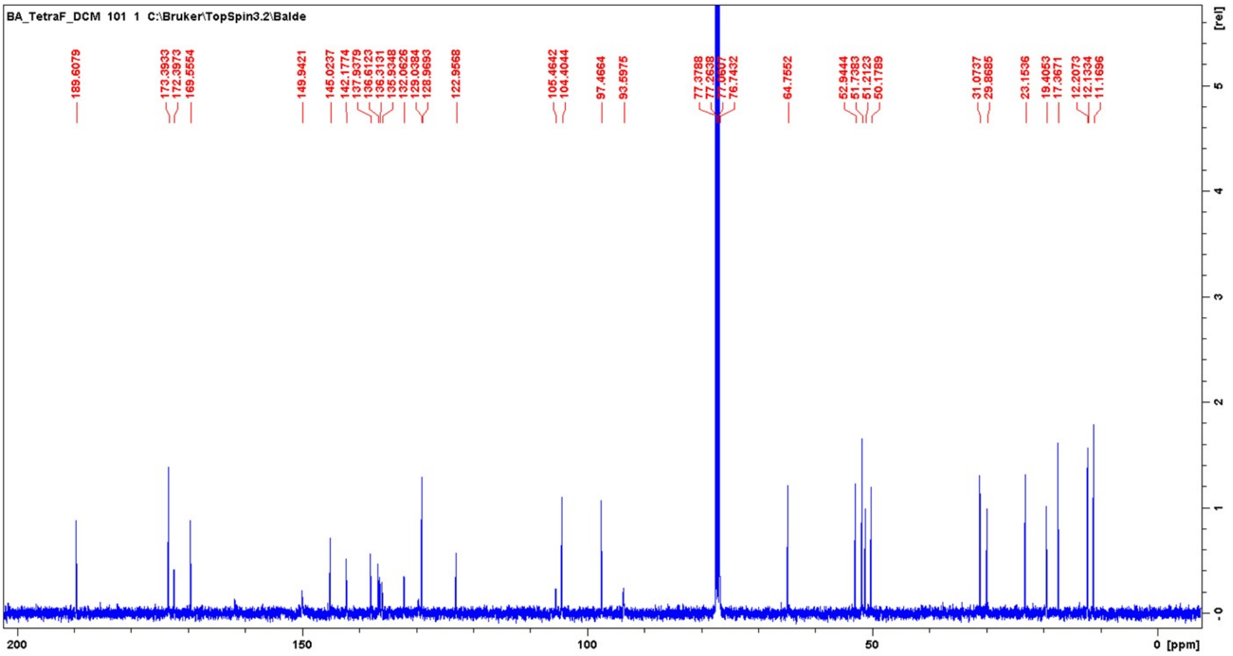

Figure S.26. ^{13}C NMR spectrum ($CDCl_3$, 100 MHz) of Pheophorbide-A methyl ester **(9)**

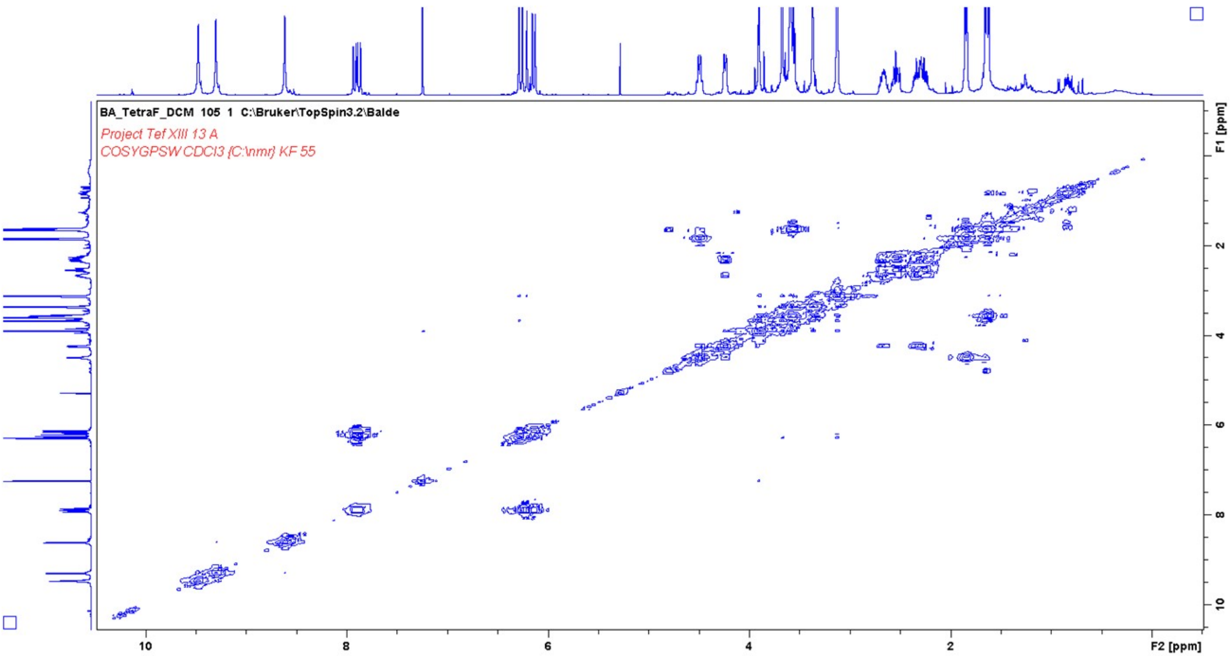

Figure S.27. COSY spectrum (CDCl3) of Pheophorbide-A methyl ester **(9)**

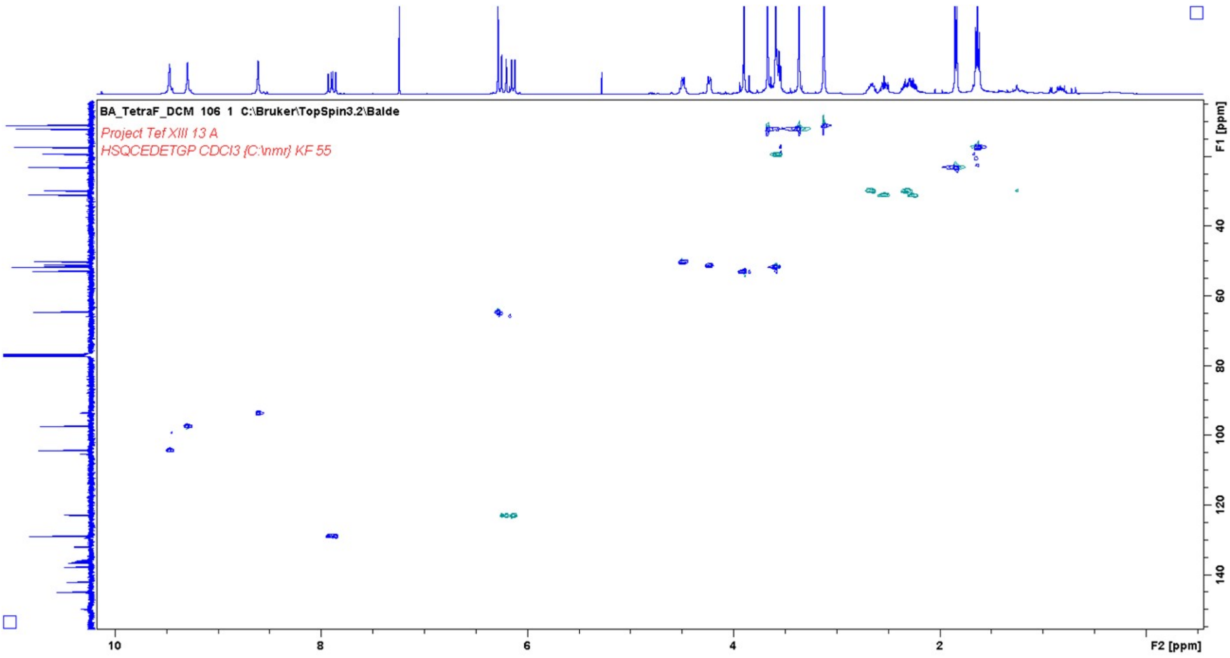

Figure S.28. HSQC spectrum (CDCl3) of Pheophorbide-A methyl ester **(9)**

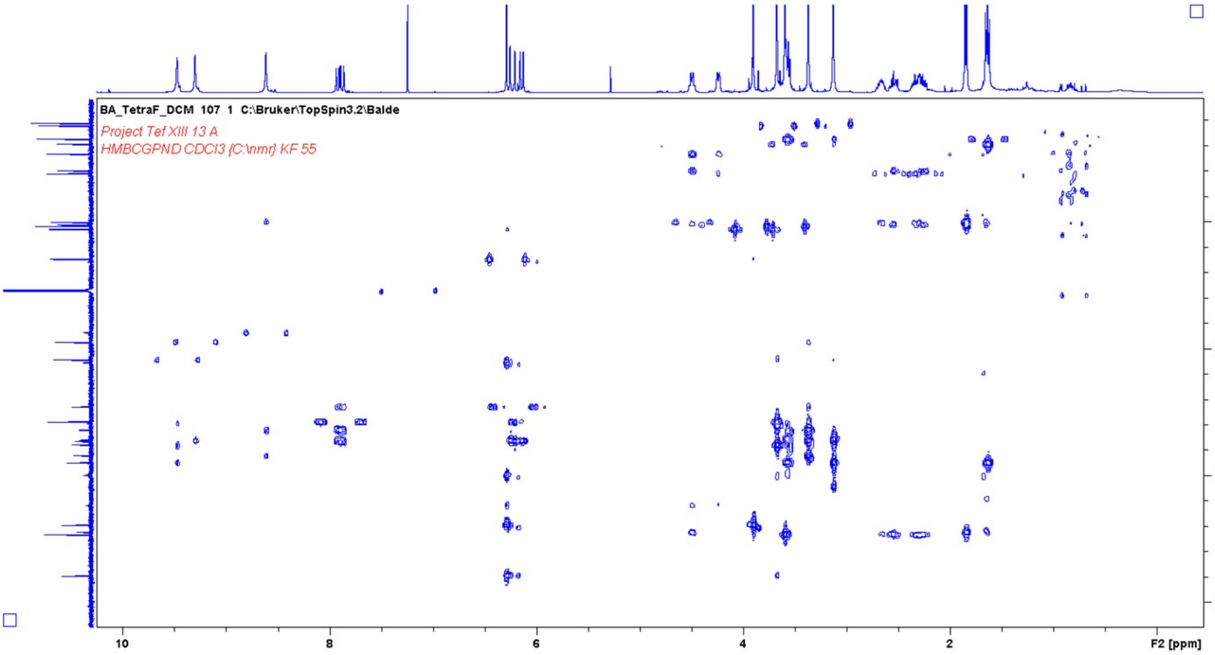

Figure S.29. HMBC spectrum ($CDCl_3$) of Pheophorbide-A methyl ester **(9)**

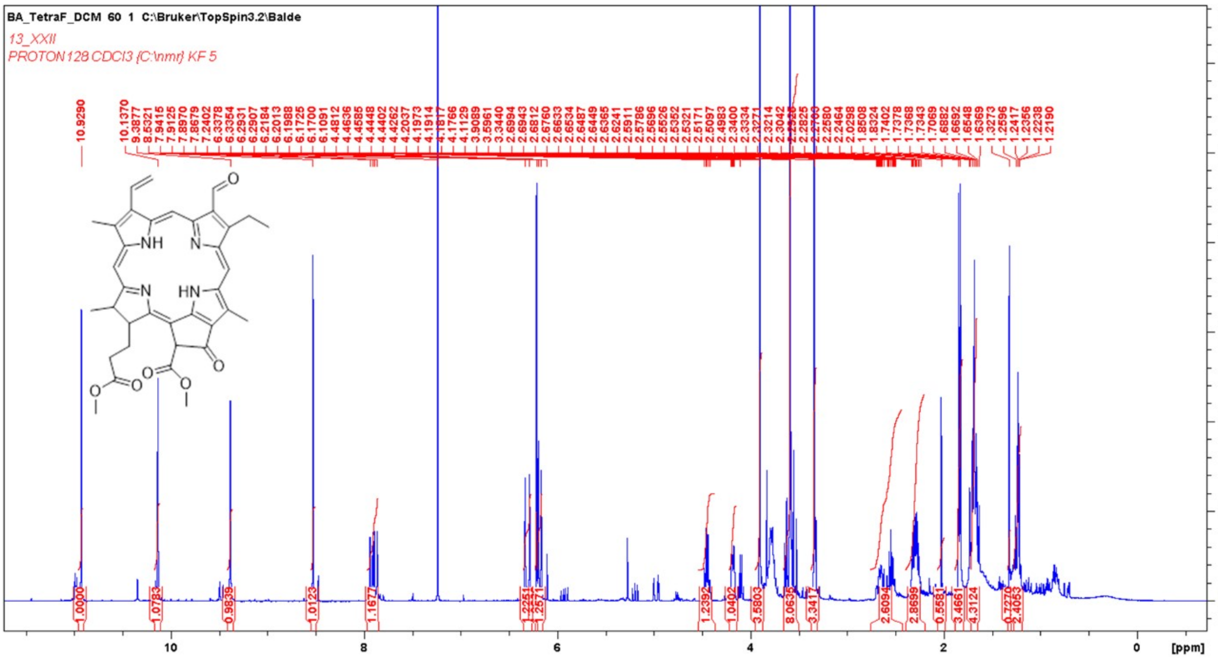

Figure S.30. ^{1}H NMR spectrum ($CDCl_3$, 400 MHz) of Pheophorbide-B methyl ester **(10)**

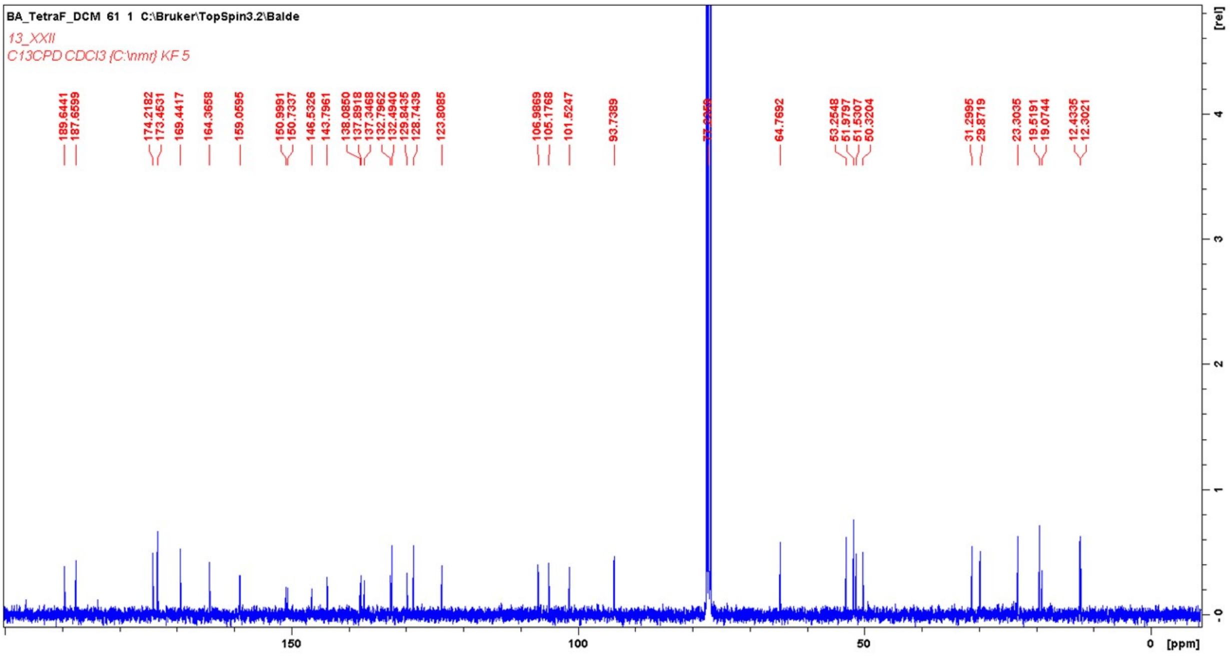

Figure S.31. ^{13}C NMR spectrum ($CDCl_3$, 100 MHz) of Pheophorbide-B methyl ester **(10)**

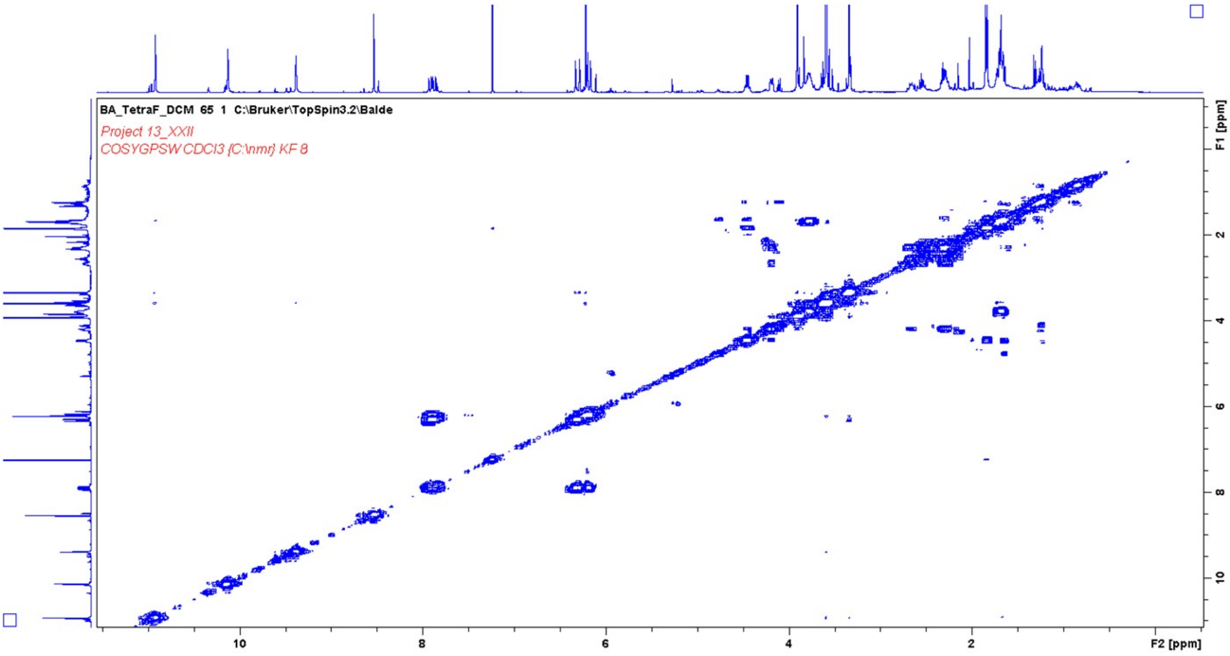

Figure S.32. COSY spectrum ($CDCl_3$) of Pheophorbide-B methyl ester **(10)**

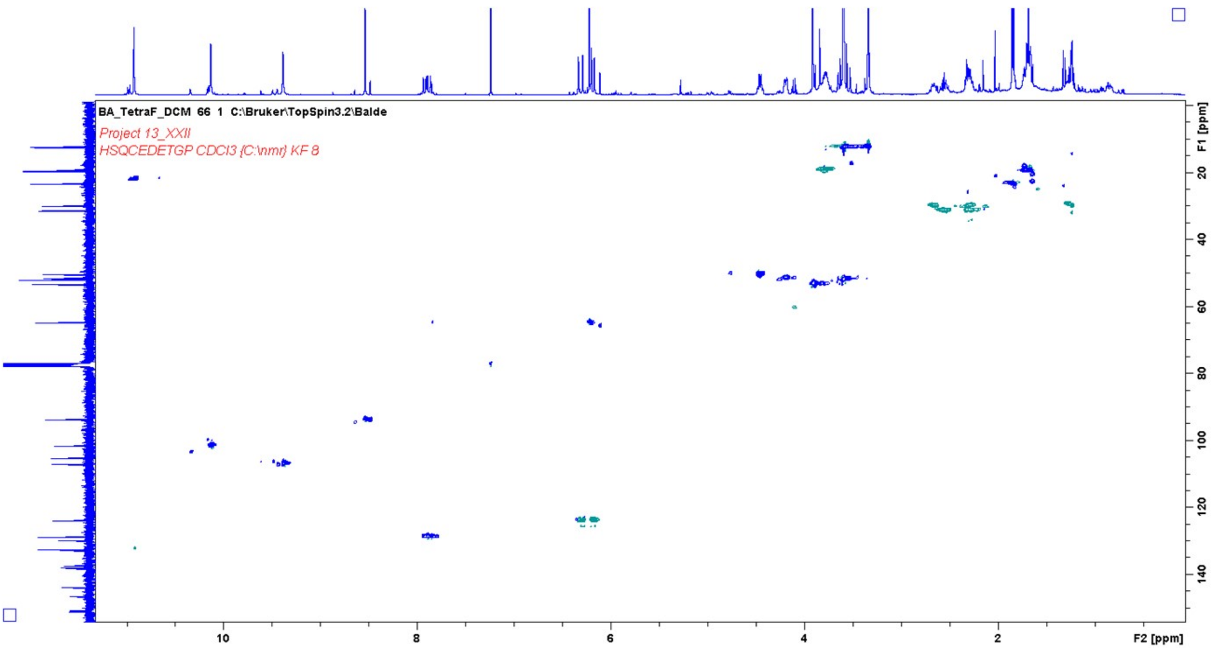

Figure S.33. HSQC spectrum ($CDCl_3$) of Pheophorbide-B methyl ester **(10)**

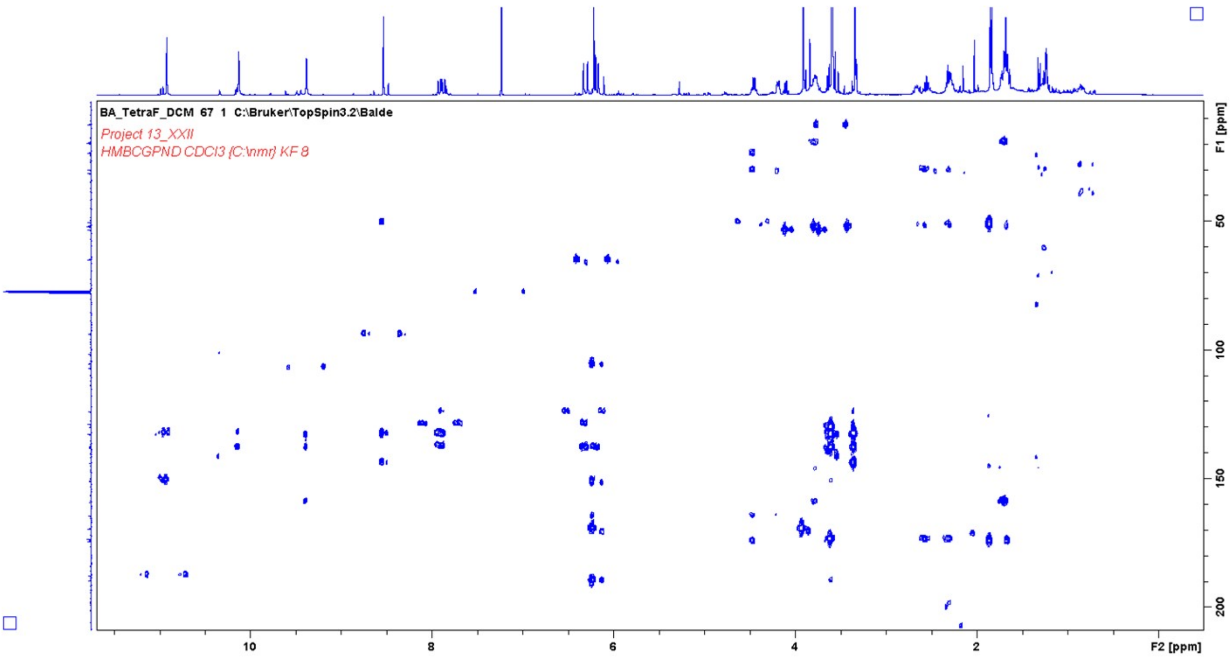

Figure S.34. HMBC spectrum ($CDCl_3$) of Pheophorbide-B methyl ester **(10)**

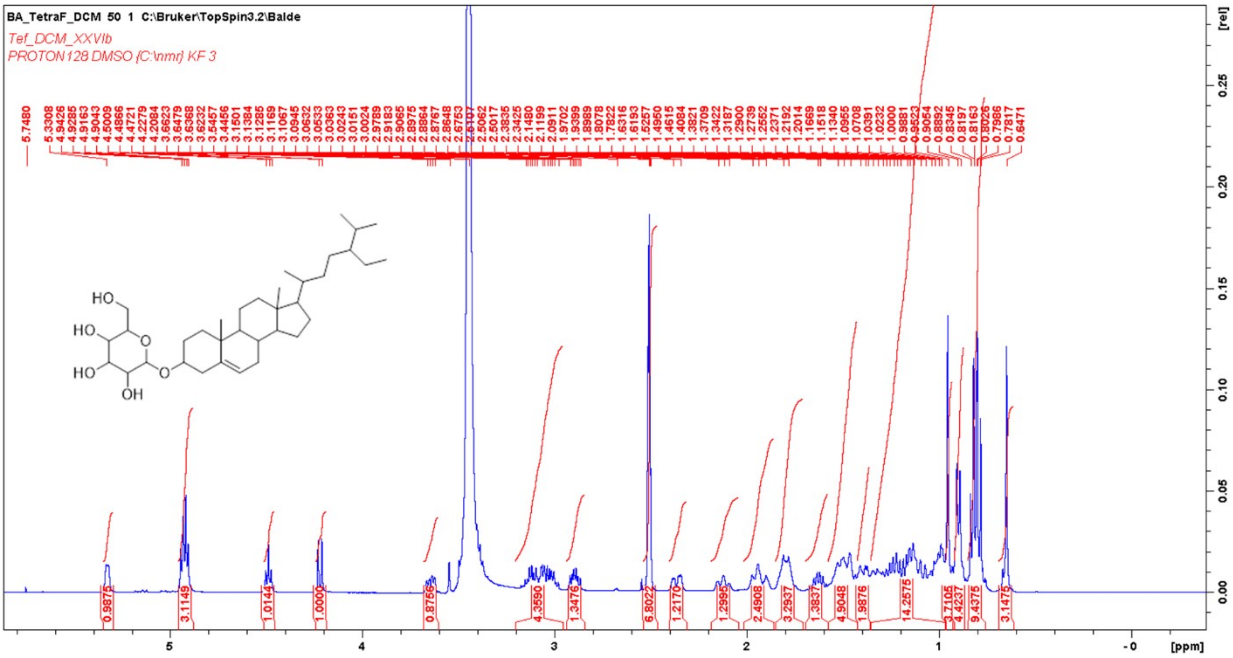

Figure S.35. ^{1}H NMR spectrum(DMSO-*d6*, 400 MHz) of Stigma-5-en-3-*O*-β-glucoside **(11)**

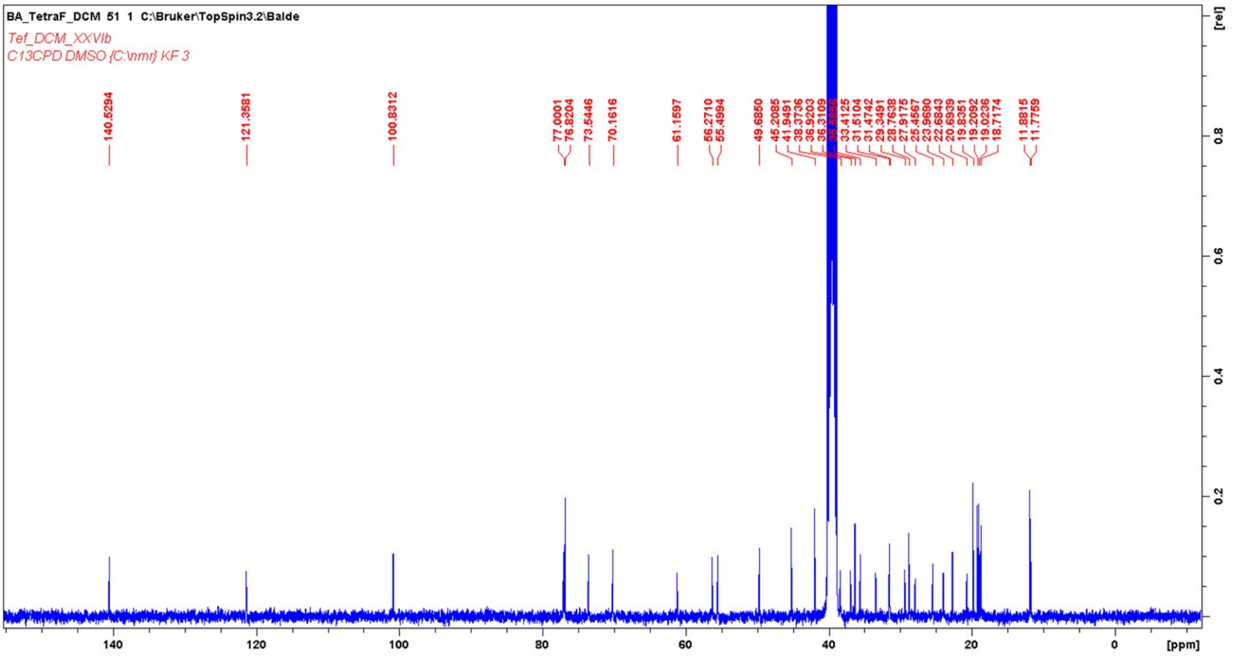

Figure S.36. ^{13}C NMR spectrum (DMSO-*d6*, 100 MHz) of Stigma-5-en-3-*O*-β-glucoside **(11)**

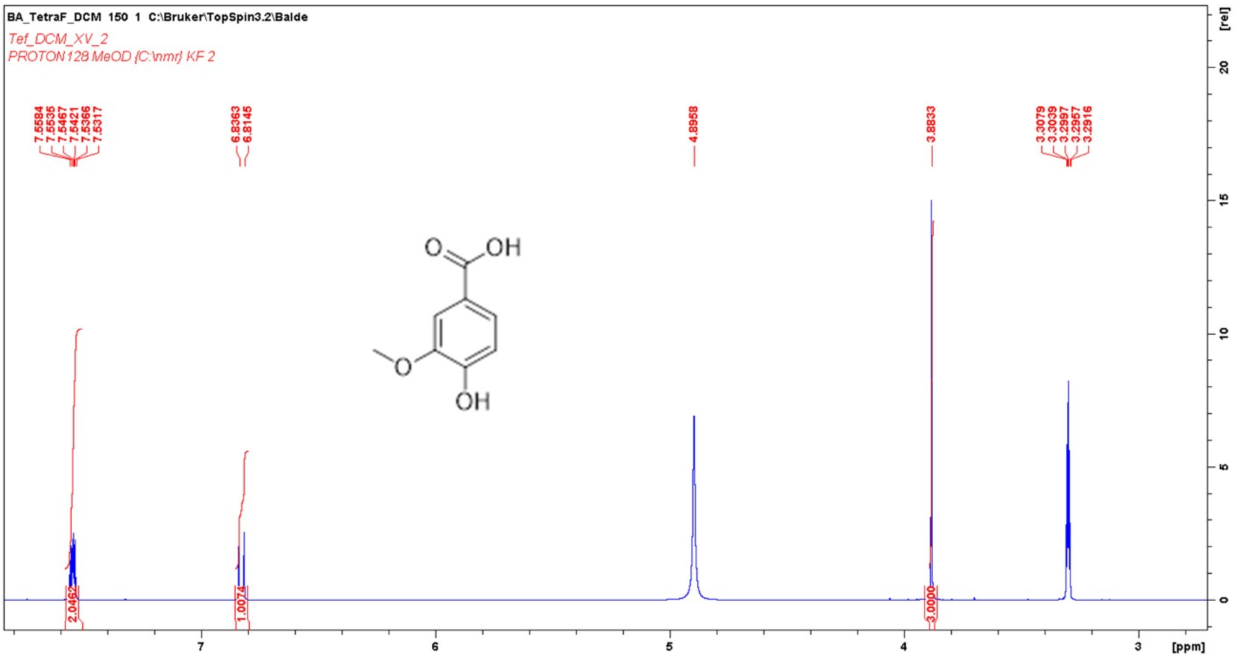

Figure S.37. ^{1}H NMR spectrum (DMSO-*d*6, 400 MHz) of Vannilic acid **(12)**

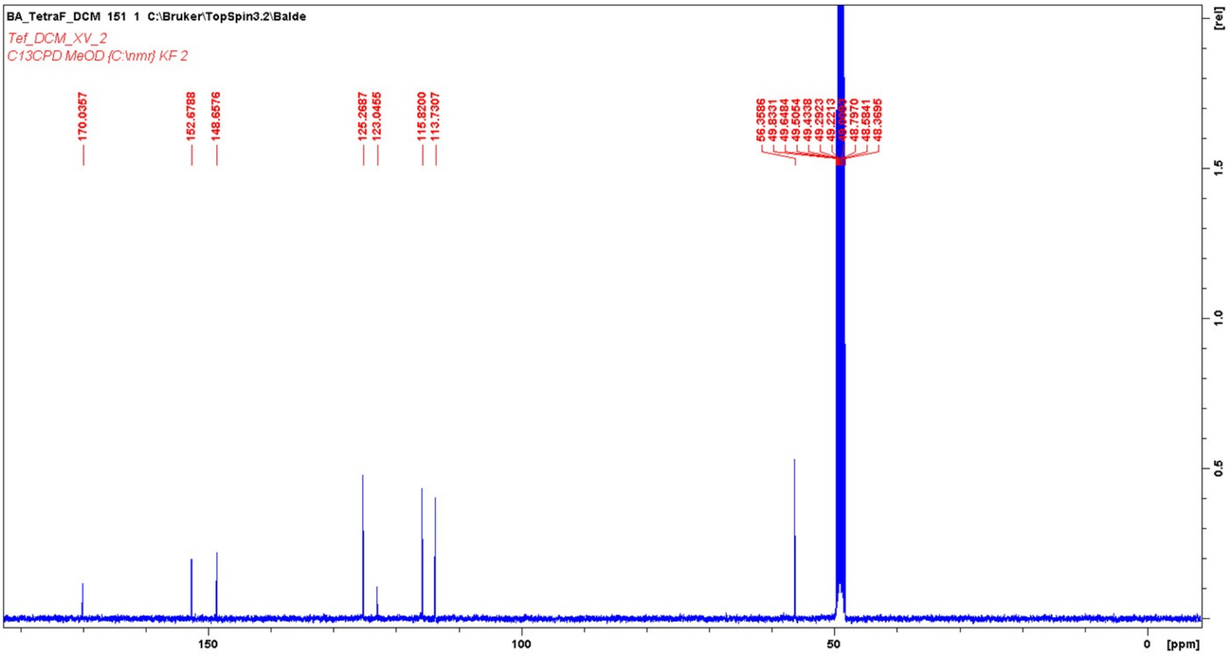

Figure S.38. ^{13}C NMR spectrum (DMSO-*d*6, 100 MHz) of Vannilic acid **(12)**

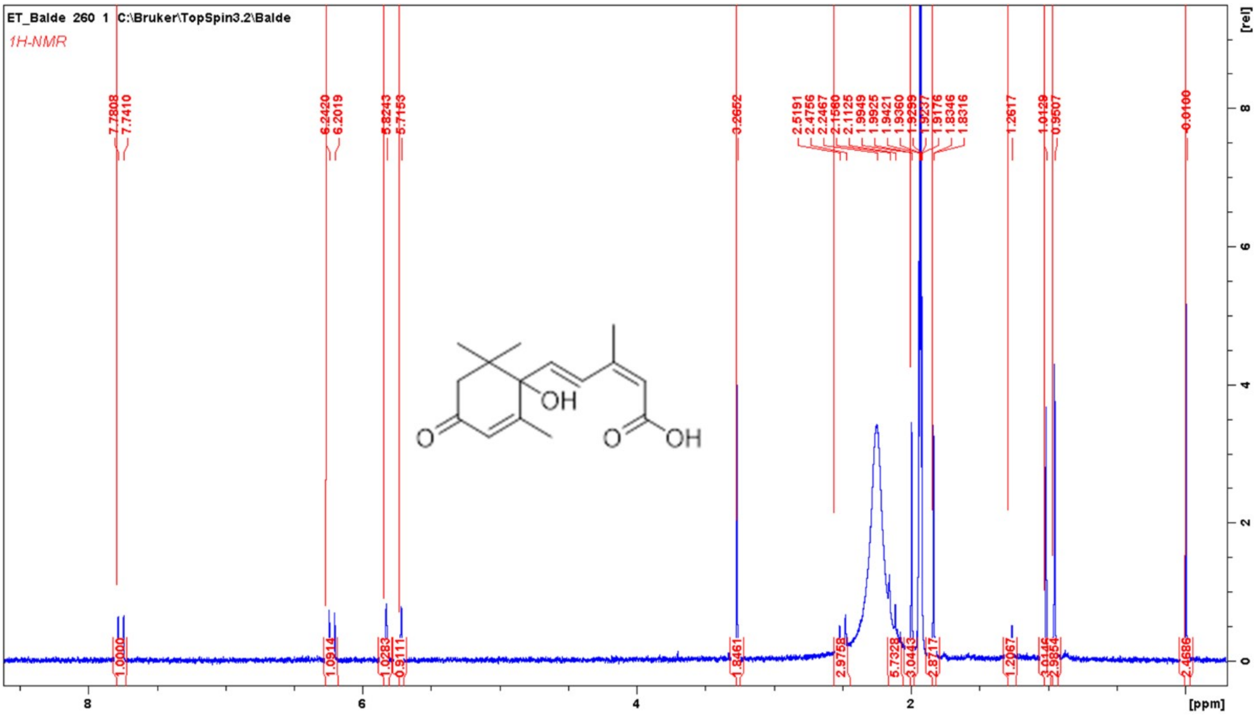

Figure S.39. ^{1}H NMR spectrum (CD_3CN, 400 MHz) of Abscisic acid **(13)**

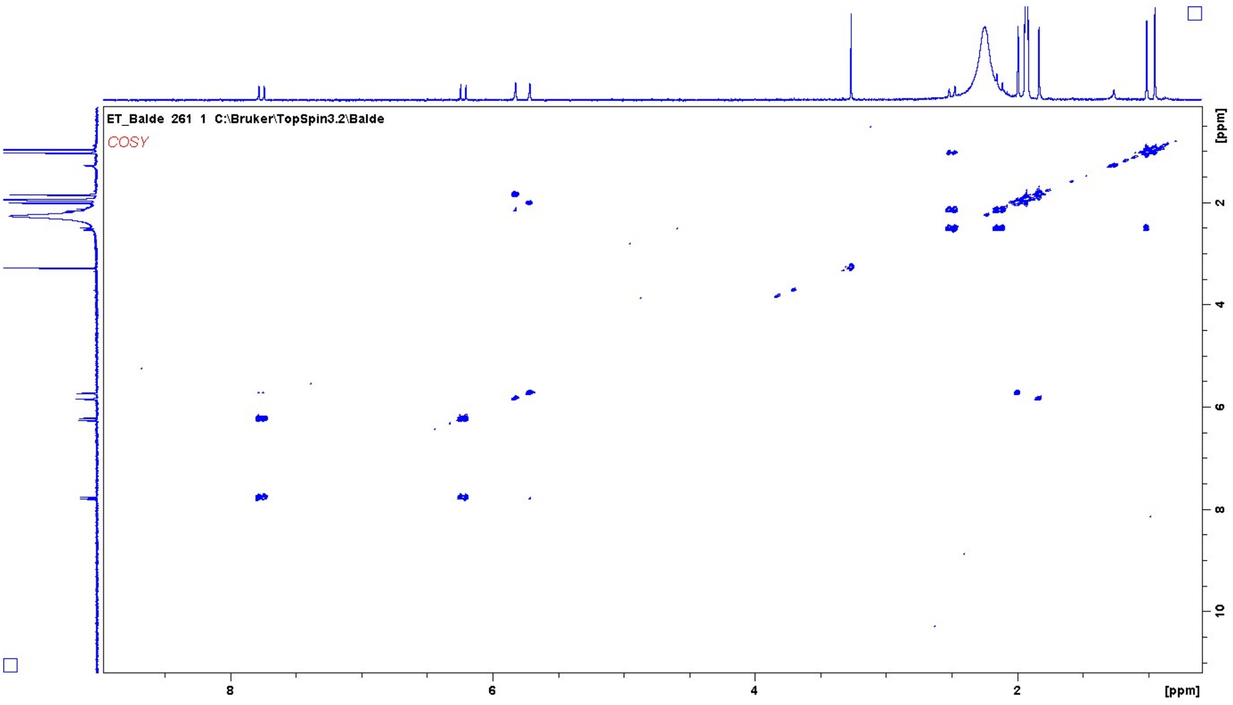

Figure S.40. COSY spectrum (CD_3CN) of Abscisic acid **(13)**

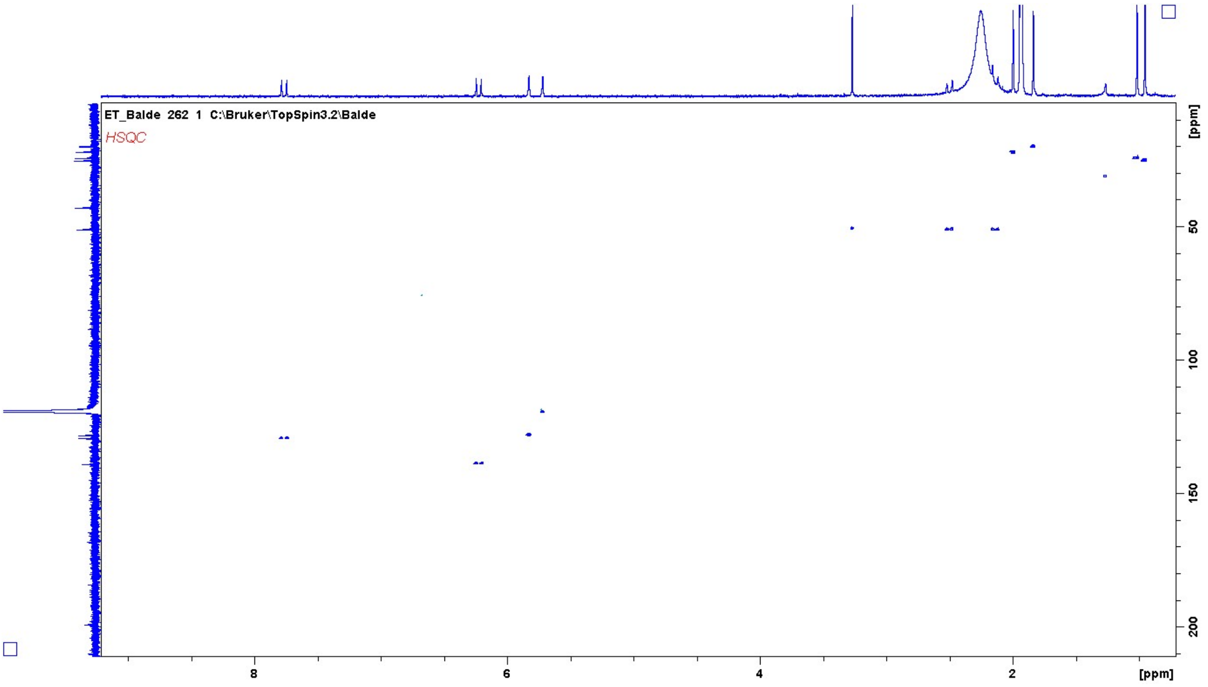

Figure S.41. HSQC spectrum (CD_3CN) of Abscisic acid **(13)**

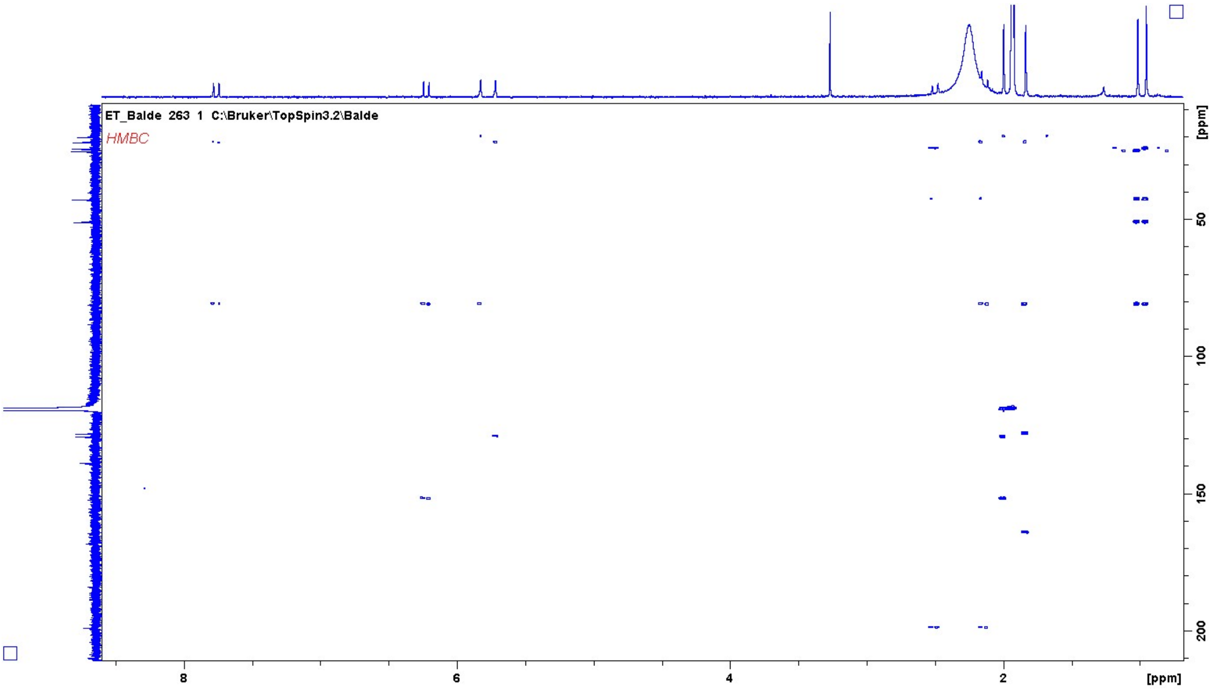

Figure S.42. HMBC spectrum (CD_3CN) of Abscisic acid **(13)**

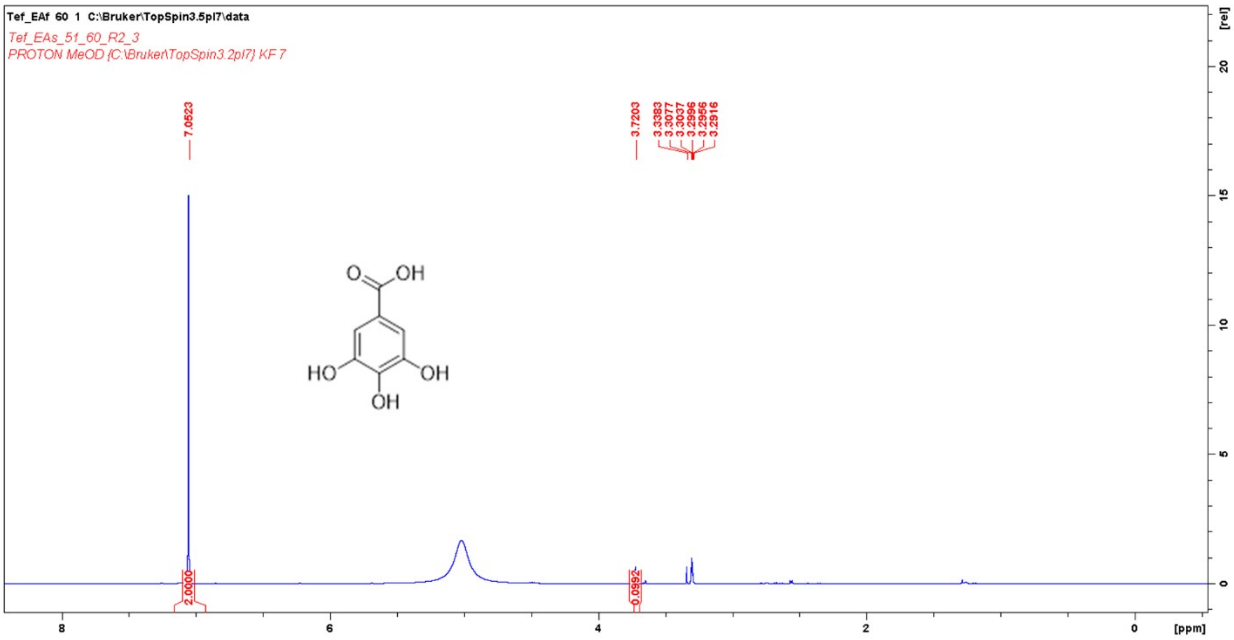

Figure S.43. ^{1}H NMR spectrum (CD_3OD, 400 MHz) of Gallic acid **(14)**

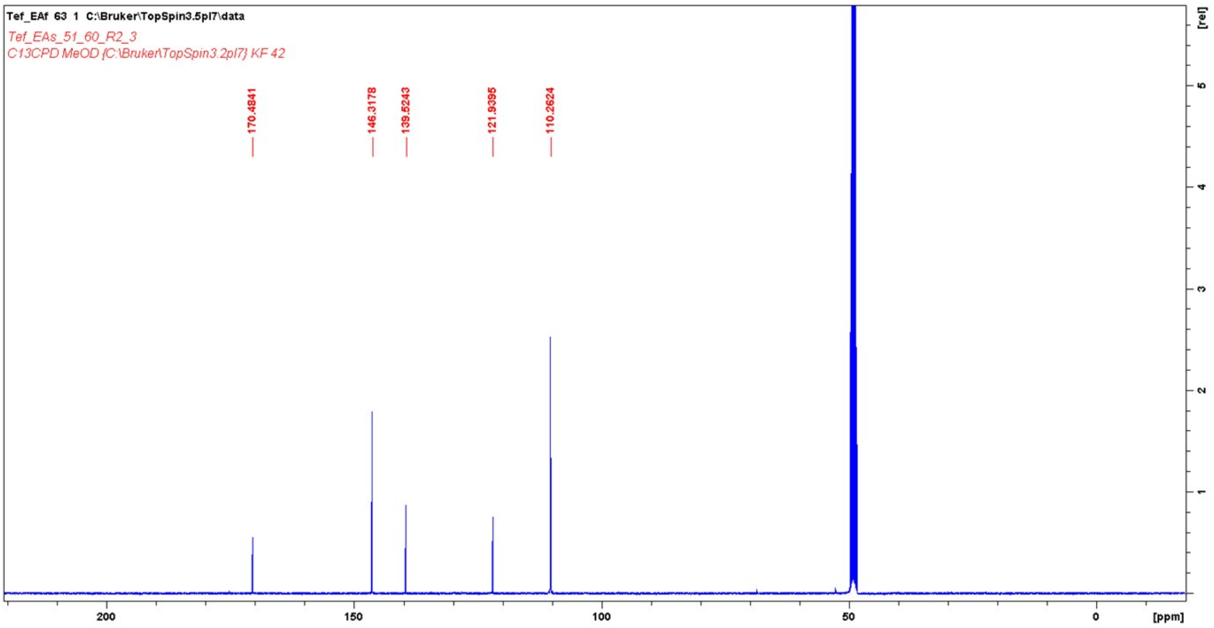

Figure S.44. ^{13}C NMR spectrum (CD_3OD, 100 MHz) of Gallic acid **(14)**

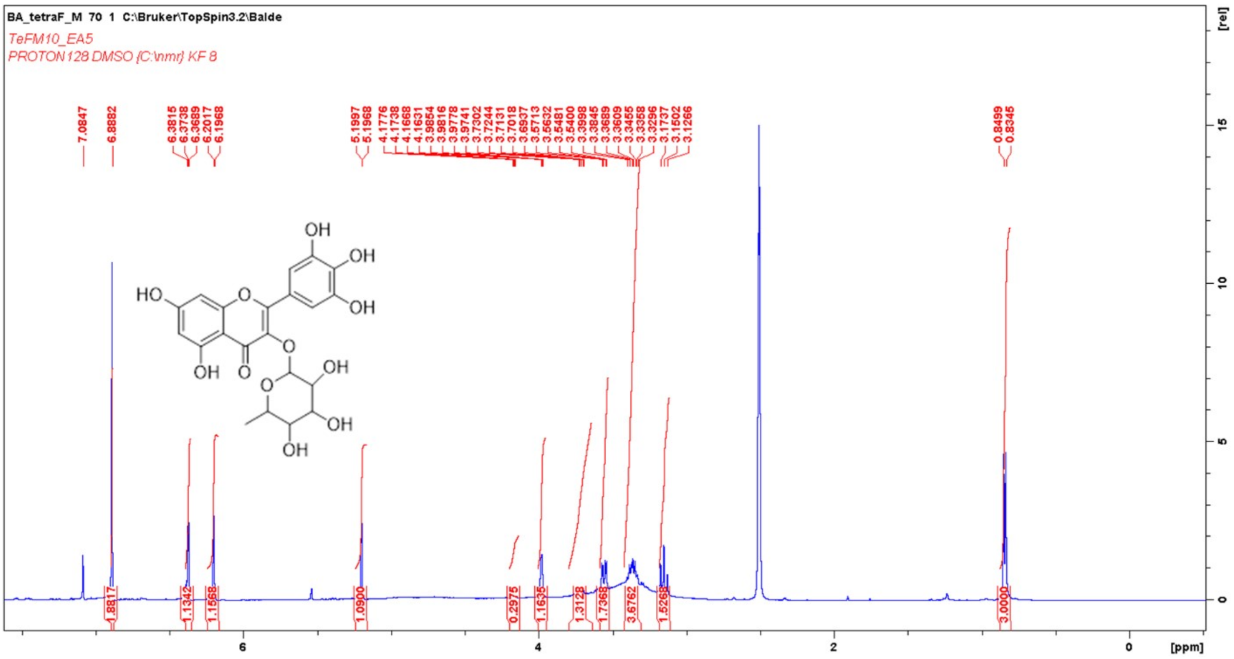

Figure S.45. ^{1}H NMR spectrum (DMSO-*d*6, 400 MHz) of Myricetin-3-*O*- rhamnoside **(15)**

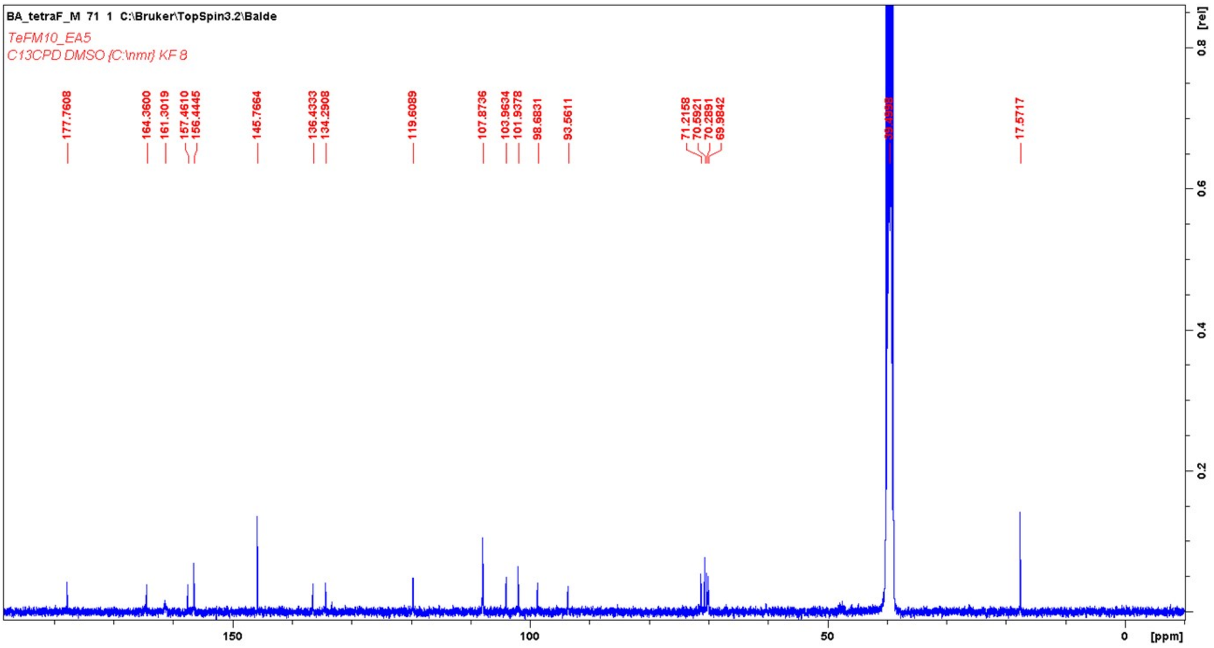

Figure S.46. ^{13}C NMR spectrum (DMSO-*d*6, 100 MHz) of Myricetin-3-*O*- rhamnoside **(15)**

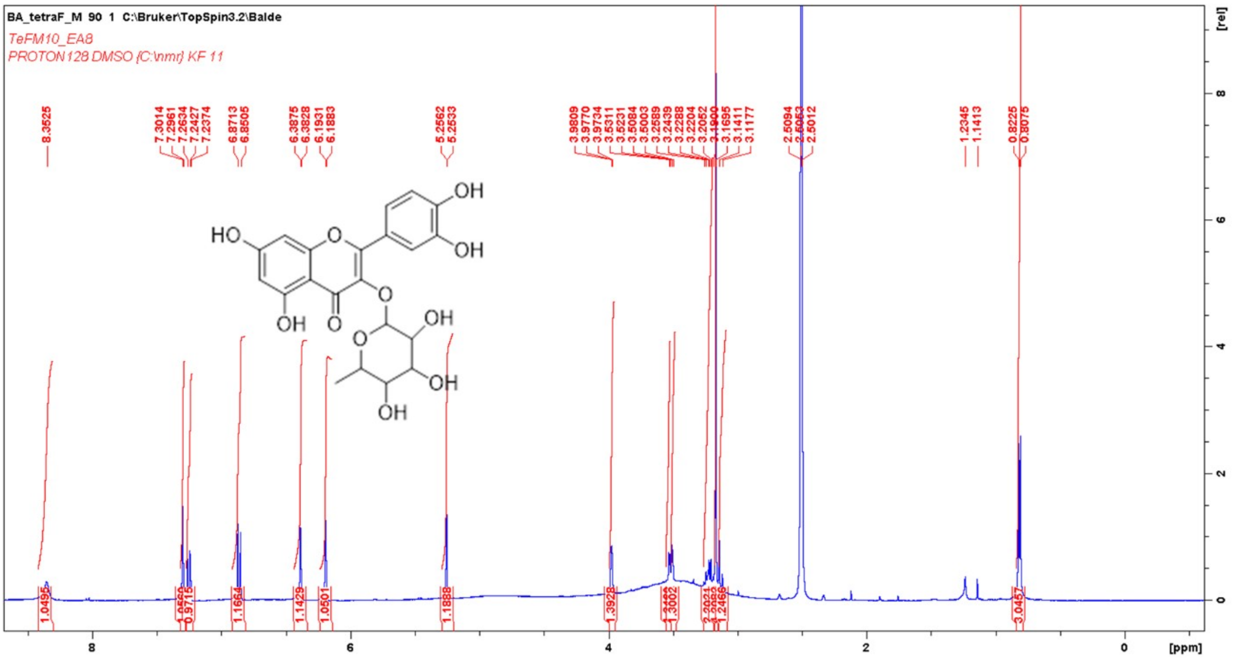

Figure S.47. ^{1}H NMR spectrum (DMSO-*d*6, 400 MHz) of Quercetin-3-*O*- rhamnoside **(16)**

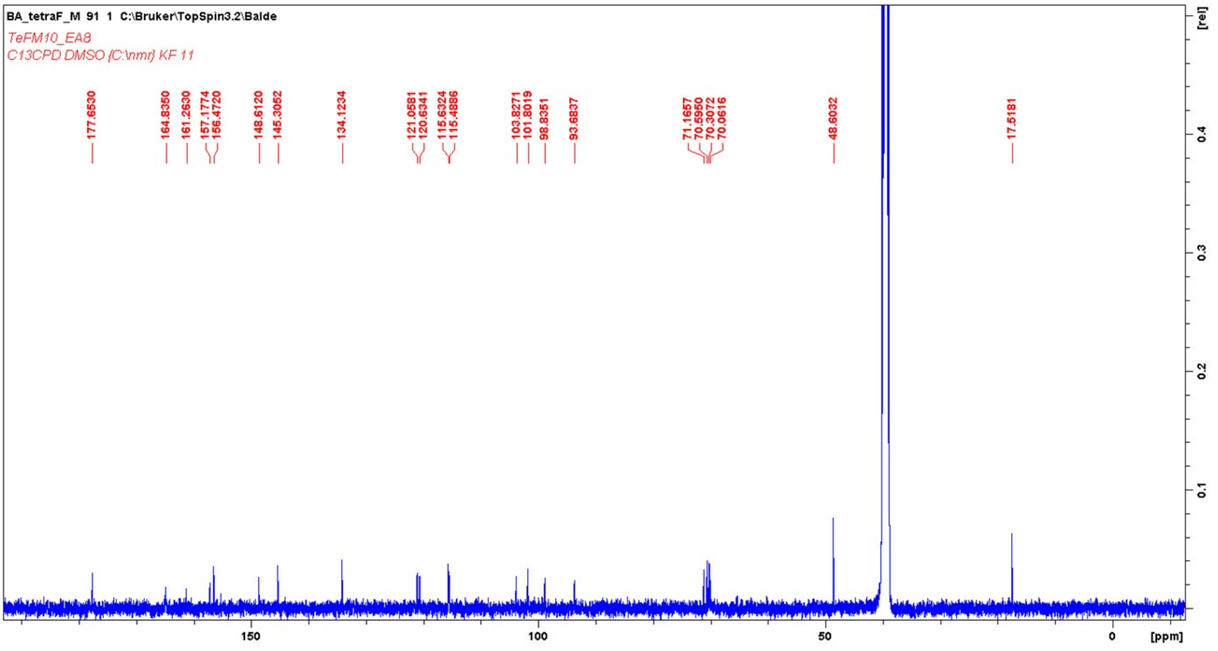

Figure S.48. ^{13}C NMR spectrum (DMSO-*d*6, 100 MHz) of Quercetin-3-*O*- rhamnoside **(16)**

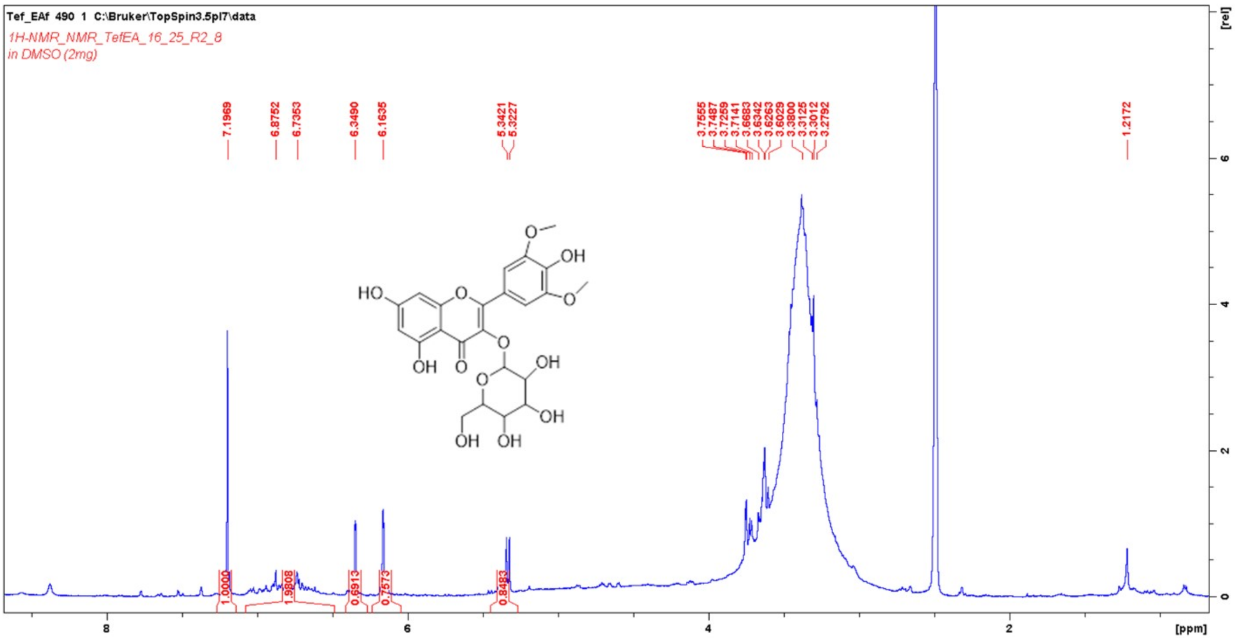

Figure S.49. ^{1}H NMR spectrum (DMSO-*d6*, 400 MHz) Myricetin-3',5'dimethylether-3*O*-Galactopyranoside **(17)**

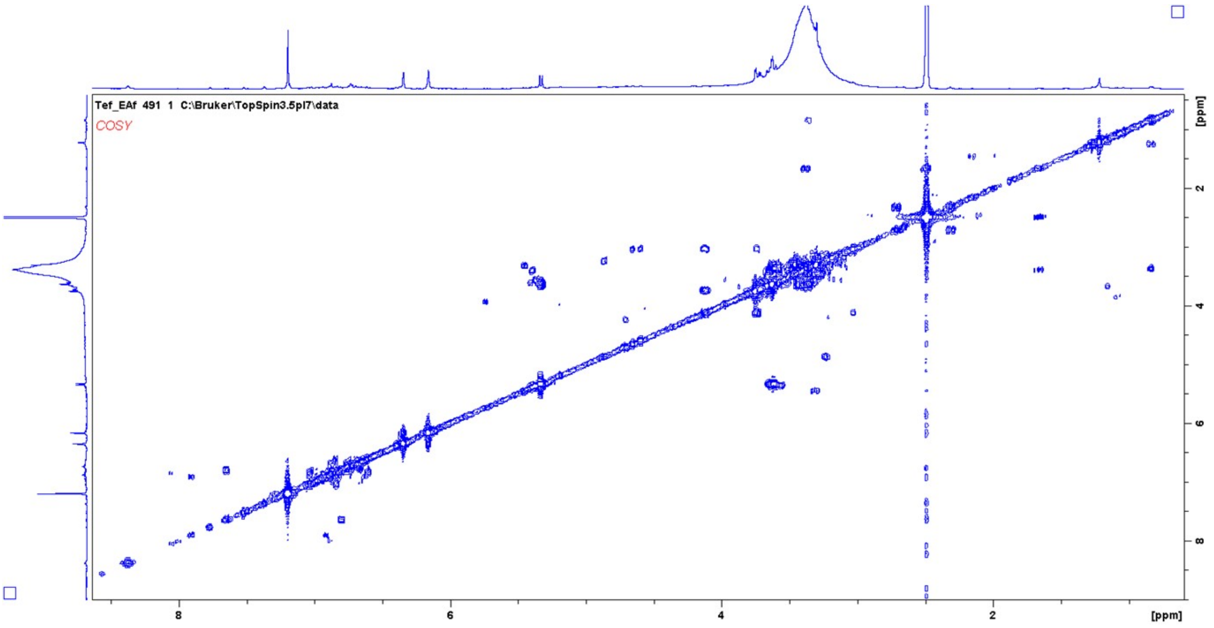

Figure S.50. COSY spectrum (DMSO-*d6*) Myricetin-3',5'dimethylether-3-*O*-

Galactopyranoside **(17)**

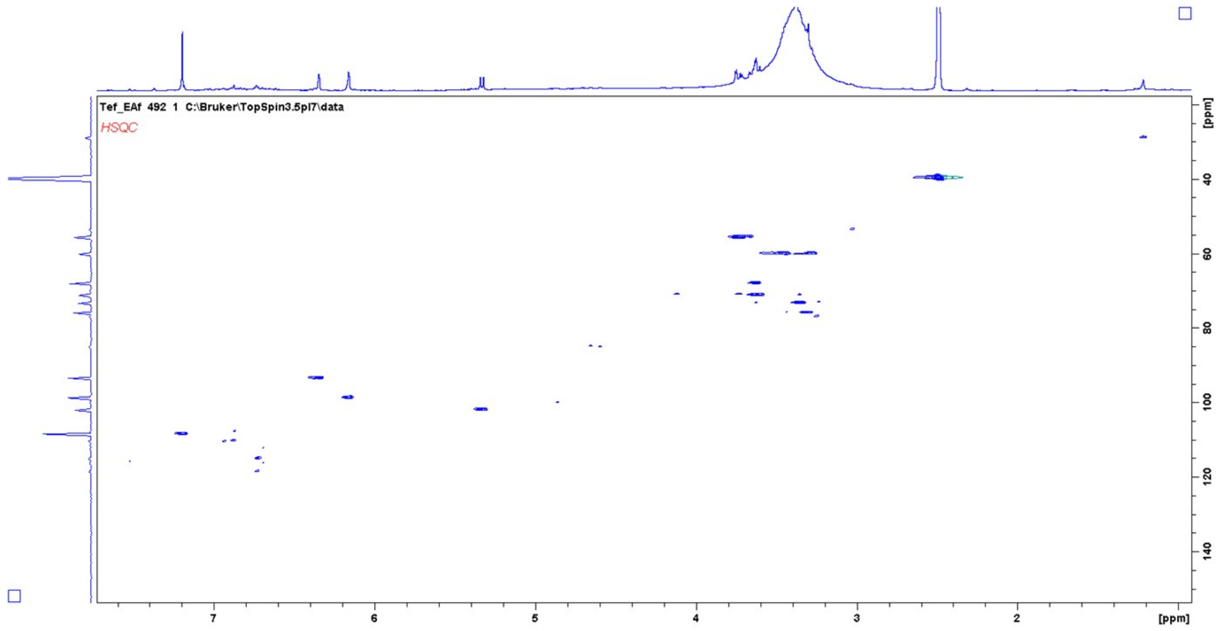

Figure S.51. HSQC spectrum (DMSO-*d6*) Myricetin-3',5'dimethyl-ether-3-*O*-Galactopyranoside **(17)**

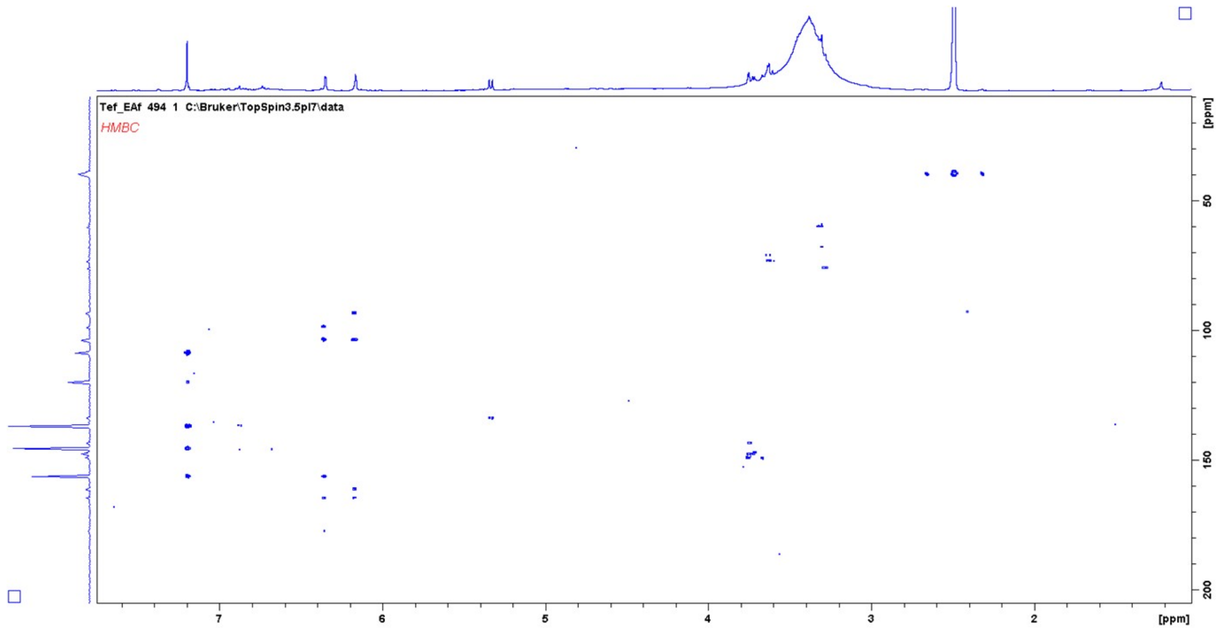

Figure 6.52. HMBC spectrum (DMSO-*d6*) Myricetin-3',5'dimethyl-ether-3-*O*-Galactopyranoside **(17)**

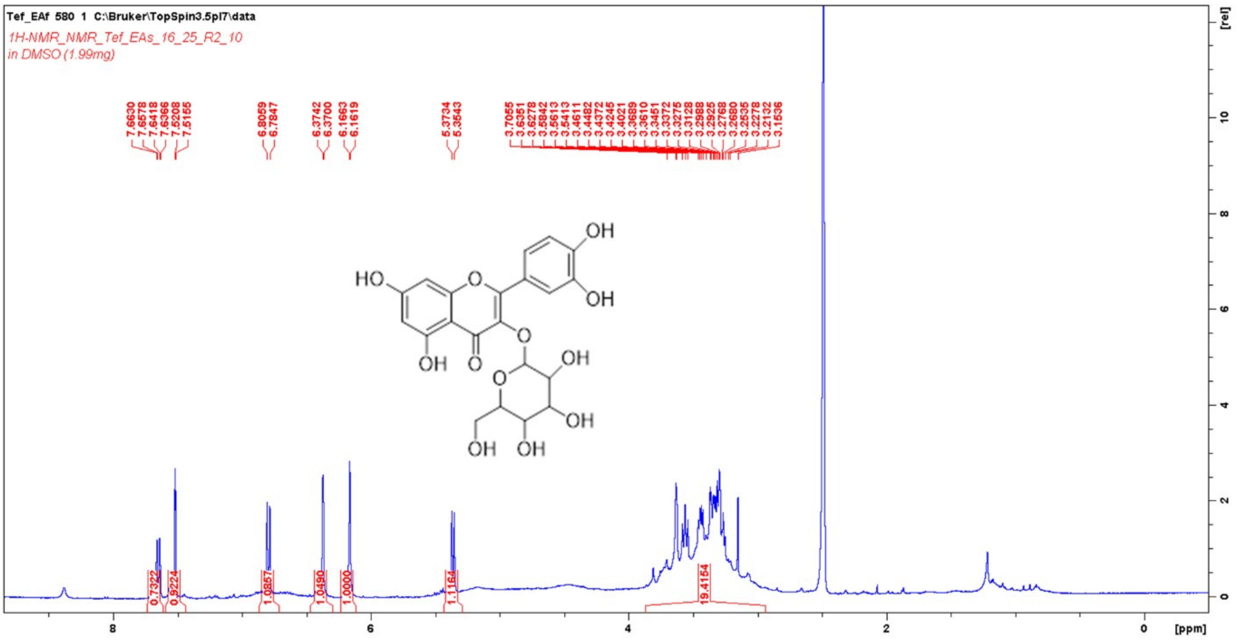

Figure S.53. ^{1}H NMR spectrum (DMSO-*d*6, 400 MHz) of Quercetin-3-*O*-galactopyranoside **(18)**

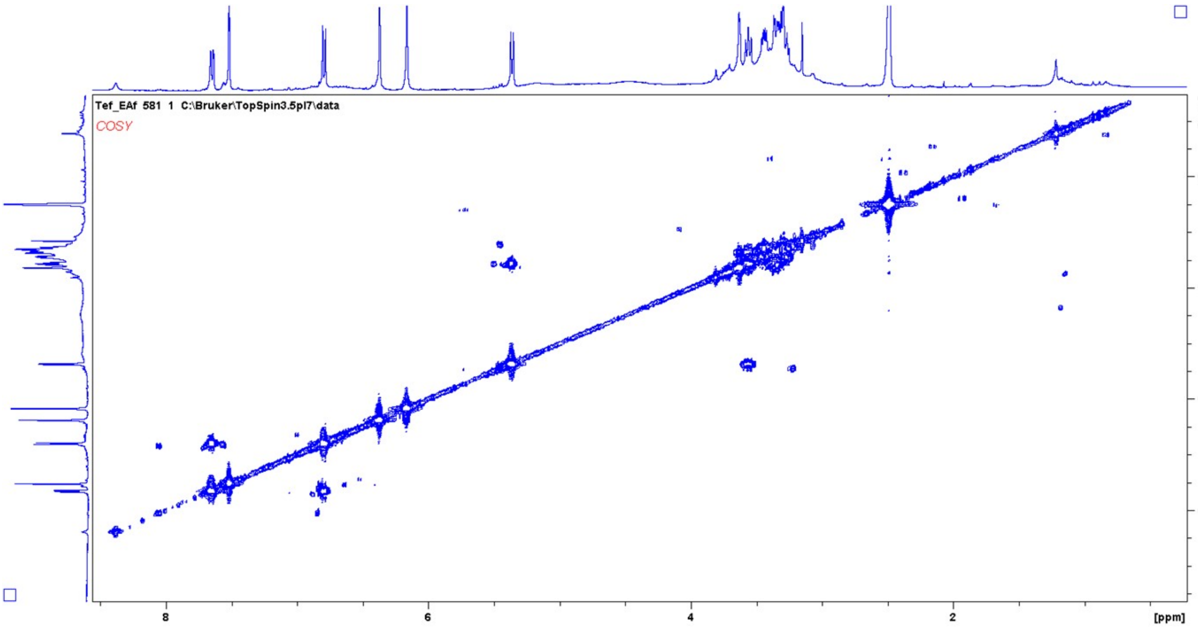

Figure S.54. COSY spectrum (DMSO-*d*6) of Quercetin-3-*O*-galactopyranoside **(18)**

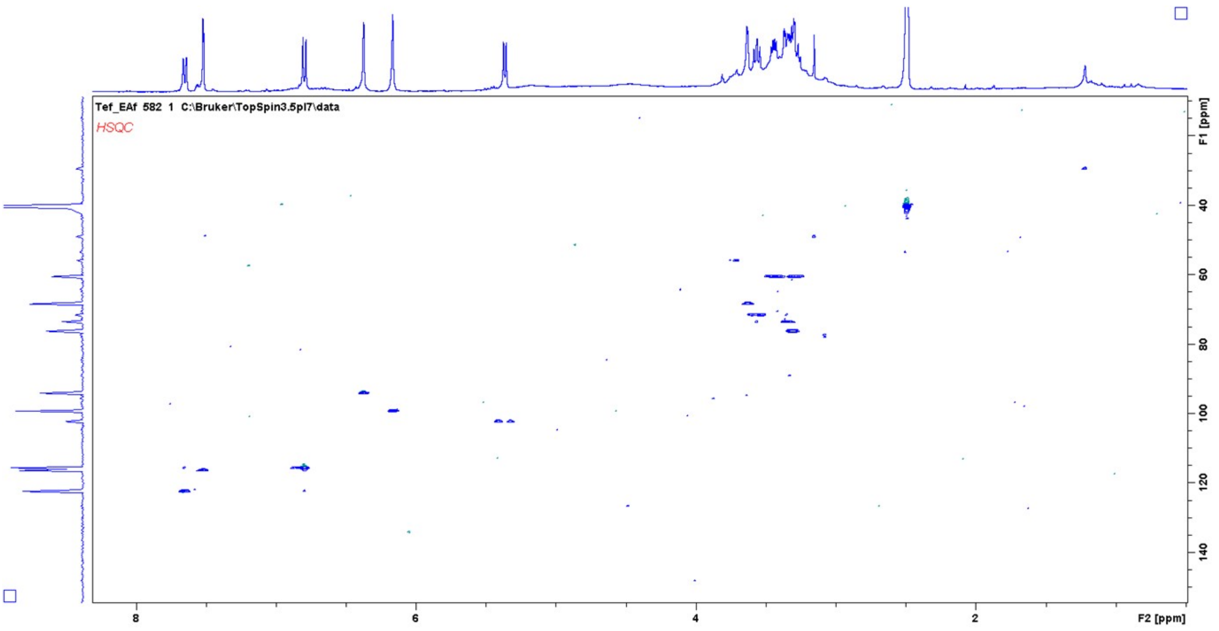

Figure S.55. HSQC spectrum (DMSO-*d*6) of Quercetin-3-*O*-galactopyranoside **(18)**

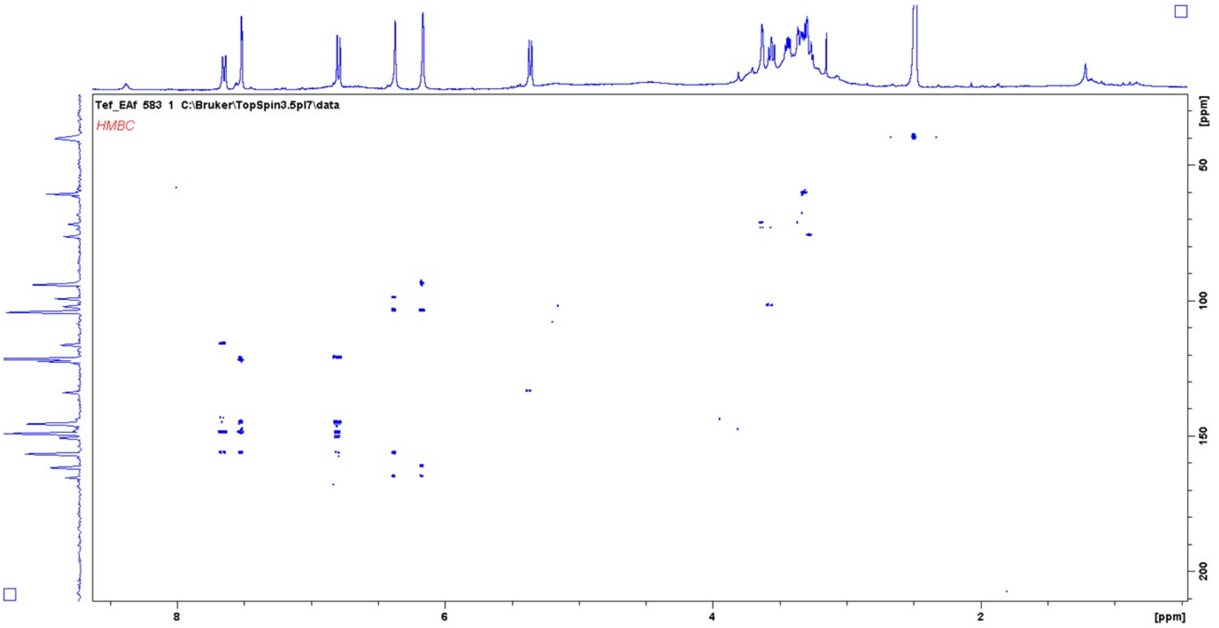

Figure S.56. HMBC spectrum (DMSO-*d*6) of Quercetin-3-*O*-galactopyranoside **(18)**

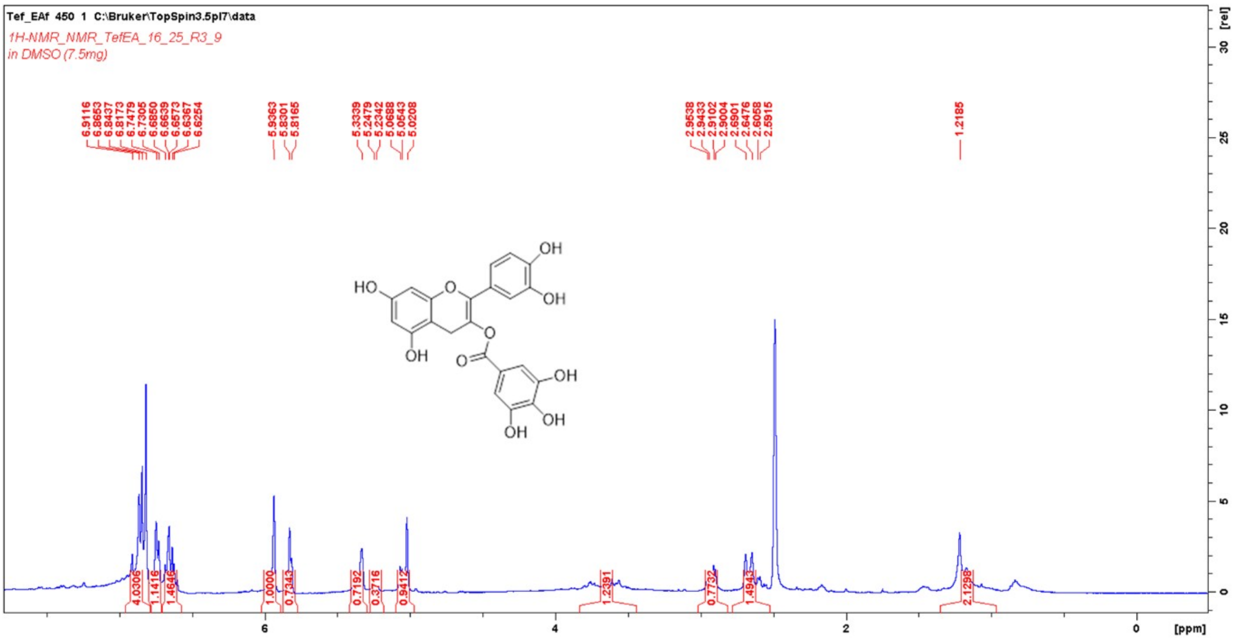

Figure S.57. ^{1}H NMR spectrum (DMSO-*d6*, 400 MHz) of Epicatechin-3-galloylester **(19)**

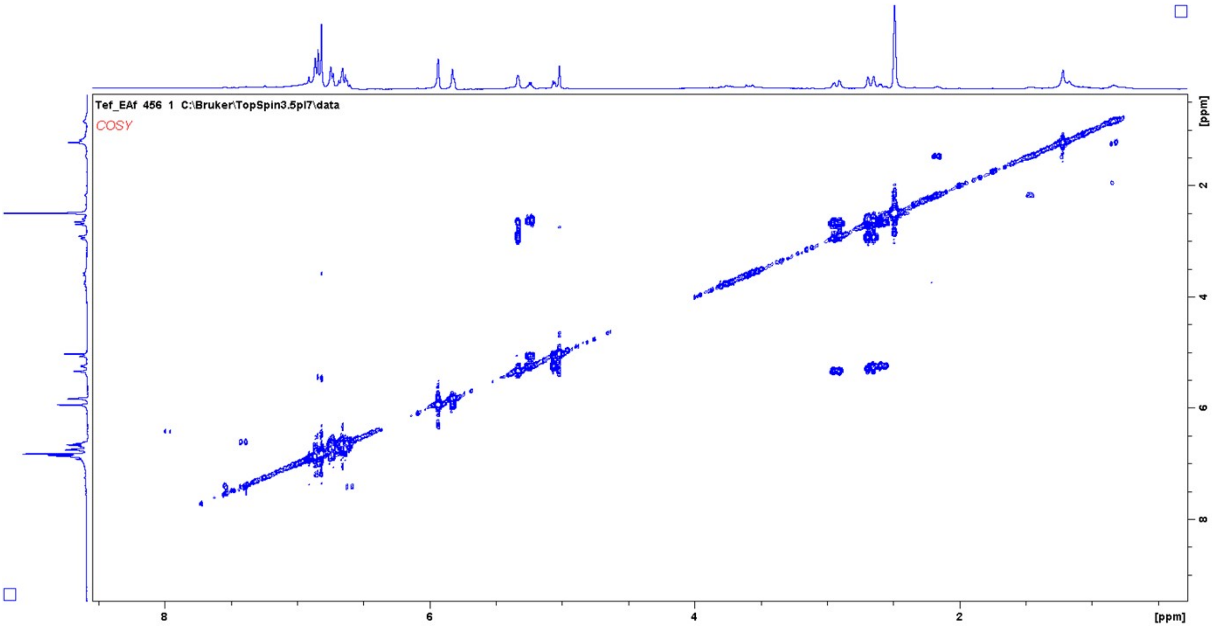

Figure S.58. COSY spectrum (DMSO-*d6*) of Epicatechin-3-galloylester **(19)**

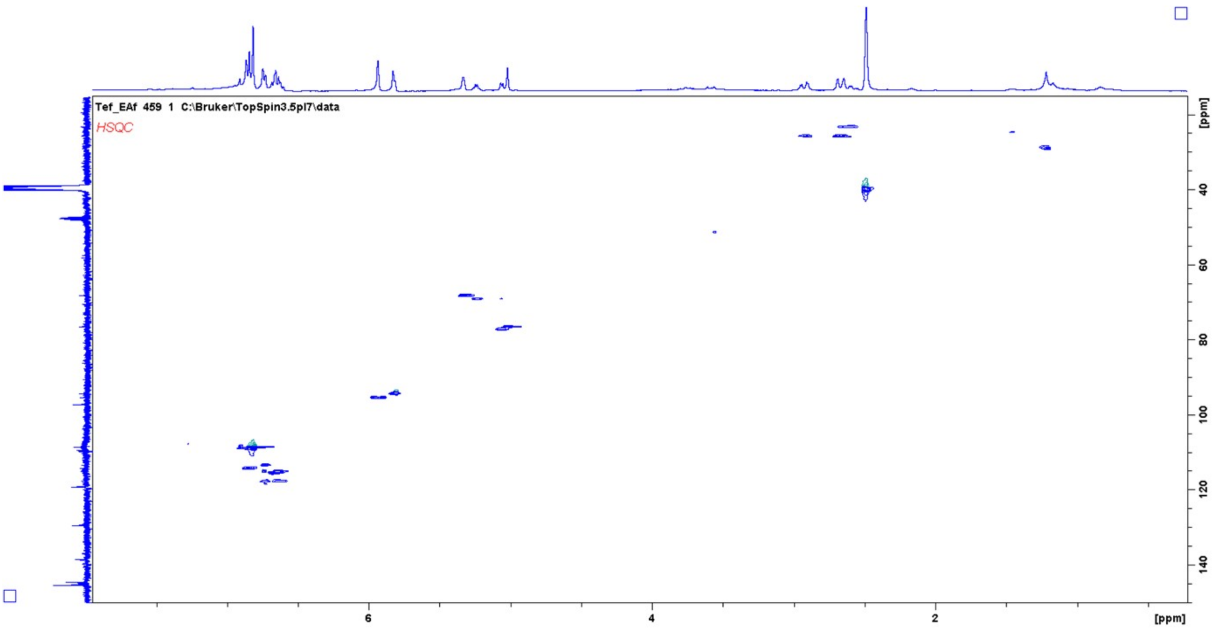

Figure S.59. HSQC spectrum (DMSO-*d6*) of Epicatechin-3-galloylester **(19)**

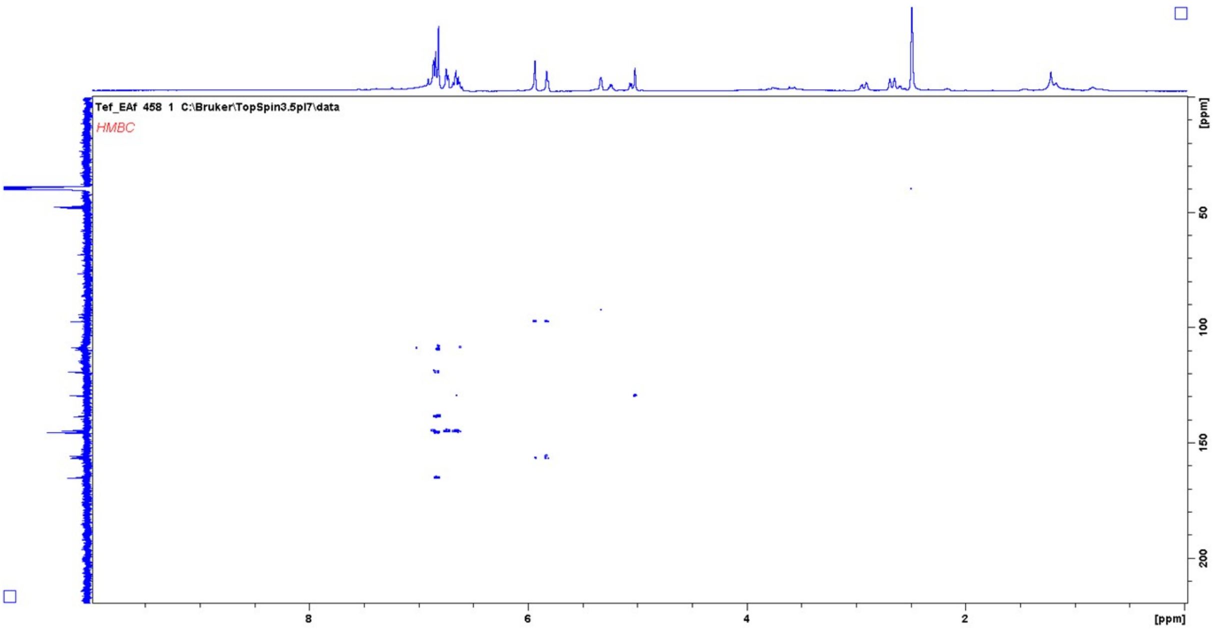

Figure S.60. HMBC spectrum (DMSO-*d6*) of Epicatechin-3-galloylester **(19)**
